# Neural mechanisms of visual quality perception and adaptability in the visual pathway

**DOI:** 10.1016/j.patter.2025.101368

**Published:** 2025-10-01

**Authors:** Yiming Zhang, Yitong Chen, Ying Hu, Xu Han, Zhenhui Xie, Xingrui Wang, Yan Zhou, Xiongkuo Min, Guangtao Zhai

**Affiliations:** 1 Institute of Image Communication and Network Engineering, Department of Electronic Engineering, Shanghai Jiao Tong University, Shanghai, China; 2 Department of Radiology, Renji Hospital, School of Medicine, Shanghai Jiao Tong University, Shanghai, China

**Keywords:** fMRI, visual quality assessment, visual perception, brain mechanisms

## Abstract

Visual quality assessment (VQA) is indispensable in multimedia for evaluating algorithm effectiveness and optimizing systems, yet its neurobiological mechanisms remain poorly understood. Using functional magnetic resonance imaging (fMRI), we investigate how the brain processes varying image qualities, revealing specialized mechanisms for handling low-quality stimuli. Results show that low quality significantly impacts semantic encoding along the visual pathway: low-level regions exhibit only 35.20% of the semantic information seen in high-quality condition, while higher-level regions compensate adaptively to maintain understanding. Visual quality is not locally encoded but emerges from inter-regional information gaps, with perception arising from this hierarchical discrepancy. Leveraging this compensatory mechanism, we decode quality from fMRI and propose a neural network feature fusion strategy, boosting ResNet’s VQA performance by 14.29% on the BID dataset (586 instances). Our findings provide neurobiological evidence for degraded visual processing, addressing a gap in perception neuroscience and offering theoretical foundations for improving VQA models.

## Highlights

• Low-level visual encoding is quality dependent; higher regions remain stable
• Visual pathways exhibit compensatory mechanisms for distorted visual inputs
• Visual quality information emerges from inter-regional information gaps
• The proposed mechanism can be validated through artificial neural networks

## The bigger picture

Visual quality assessment (VQA) plays a crucial role in real-world applications such as multimedia content distribution, video streaming, and computer vision, where ensuring the optimal quality of visual data directly impacts user experience and system performance. In this context, understanding the neural mechanisms behind visual quality perception is vital for enhancing the effectiveness of VQA models. This research bridges a gap in VQA by investigating how the human brain processes visual distortions of varying quality using functional magnetic resonance imaging (fMRI). The findings reveal that visual quality is not locally encoded in any single brain region but emerges from the interaction between lower-level and higher-level visual areas. This understanding challenges traditional VQA methods, which typically rely on static features, by highlighting the brain’s compensatory mechanisms in handling low-quality signals.

Beyond the scope of VQA, this study holds significant implications for cognitive neuroscience and the field of human visual perception. It provides new insights into how the brain processes degraded visual stimuli, shedding light on the adaptive mechanisms that allow humans to maintain semantic understanding even when image quality is compromised. These findings deepen our understanding of the hierarchical structure of the visual system and the complex cognitive processes involved in perceiving and interpreting low-quality visual information. The study’s integration of cognitive theories, such as the free energy principle (FEP), further strengthens its relevance to both neuroscience and AI. It offers a theoretical basis for the development of brain-inspired AI systems capable of mimicking human visual perception. These advancements could enhance AI’s ability to process distorted or noisy data while maintaining high performance on clean images, thus providing a more accurate and adaptive approach to VQA.

## Teaser

Visual quality assessment is crucial for both human perception and artificial vision systems. This study reveals how the human brain naturally perceives image quality through distinct neural pathways. Functional magnetic resonance imaging results show that while low-level visual areas are highly sensitive to distortions, higher-level regions compensate to maintain recognition, much like how AI systems might “fill in” missing details. These findings not only deepen our understanding of human vision but also provide biological inspiration for developing more robust image-processing algorithms.

## Introduction

Visual quality assessment (VQA), which is a crucial task in computer vision, evaluates the quality of visual content using objective algorithms that mimic and approximate the subjective quality perceived by the human visual system. Video traffic was projected to comprise 74% of total mobile data traffic by the end of 2024, driven by the expansion of internet communication and video-sharing platforms.^1^ However, distortions occur at nearly every stage of visual communication, including acquisition, compression, transmission, and display.^2^ VQA serves as a cornerstone for evaluating and optimizing the performance of visual communication systems and generative models.^3^^,^^4^ As a bridge between computer vision and human perception, accurate visual-quality metrics are essential for ensuring high-quality visual content that aligns with human perception.

VQA metrics are divided into subjective and objective types. Objective metrics, such as mean square error, peak signal-to-noise ratio, and structural similarity index measure,^5^ automatically quantify visual quality by comparing to a reference image. These widely used methods rely on signal fidelity or structural similarity due to their simplicity and repeatability but often do not fully align with human perception, especially in complex distortions.^5^ Subjective assessment is the gold standard because it involves human perception rather than algorithmic estimation. In contrast, full-reference and no-reference assessments are typically objective metrics computed by algorithms. Full-reference metrics require access to an original reference image. In contrast, no-reference metrics estimate quality without any reference, which is especially relevant for user-generated content (e.g., on social media) that may lack a version without distortions.^6^^,^^7^

Given the interdisciplinary nature of VQA, numerous studies in this field have focused on integrating theories of human visual perception into the development of quality evaluation metrics and algorithms.^8^^,^^9^ In particular, psychovisual studies on image and video perception^10^^,^^11^^,^^12^^,^^13^^,^^14^ demonstrate that current research actively explores the perceptual mechanisms of the human visual system that influence the design of quality assessment metrics and algorithms. Despite rapid algorithmic developments,^15^^,^^16^^,^^17^^,^^18^ VQA lacks the research on perceptual processes and neuropsychology principles, with limited neuroscience studies specifically addressing visual quality perception and the processing mechanisms of low-quality visual signals. In contrast, studies on semantic information processing in visual signals are abundant,^19^^,^^20^^,^^21^^,^^22^^,^^23^^,^^24^ and the similarity between deep neural networks used in semantic recognition tasks and the human visual system has also been extensively discussed.^25^^,^^26^^,^^27^

Due to the lack of a solid neuroscience foundation, existing VQA models, largely based on deep learning, rely heavily on training data and exhibit limited generalizability across datasets with different formats or sources. New media signals, such as user-generated content,^28^^,^^29^ omnidirectional images,^30^^,^^31^^,^^32^ and AI-generated content,^33^^,^^34^^,^^35^ require separate datasets and models for effective evaluation, posing practical challenges. Exploring human brain mechanisms for visual quality perception could enhance the generalizability and accuracy of these algorithms. Additionally, humans can often identify semantic information in noisy or degraded images, while current prediction models still lack similar robustness.^36^ Investigating how the brain processes low-quality visual information may inspire improvements in semantic recognition algorithms. This study aims to use functional magnetic resonance imaging (fMRI) to explore brain states related to human perception of visual signals at varying quality levels, filling in the gap of neuroscience in VQA.

Traditional VQAs collect subjective opinions on image or video quality through scales and compute the mean opinion score (MOS) to represent the visual quality. Due to their ease of implementation, these scale experiments are widely used in quality assessments. However, some researchers argue that psychophysiological tests relying on rating scales inherently relate to conscious responses and may not effectively reveal the underlying perceptual and cognitive processes. Consequently, researchers have extracted features from physiological signals, such as electroencephalography (EEG), as objective indicators of human perceptual quality, thereby avoiding potential biases caused by high-level cognitive involvement.^37^ Notably, various studies^38^^,^^39^^,^^40^^,^^41^ report that event-related potential (ERP) reactions of EEG reflect the human brain’s response to image quality degradation. However, EEG-based studies on visual quality often use a full-reference design, presenting participants with a high-quality reference image or video followed by a synthetically distorted version, where their differences strongly correlate with image distortion levels. Consequently, it is challenging to discern whether ERP reactions are due to differences between reference and distorted images or visual quality alone in such designs. Therefore, the existing measurement methods based on scales and EEG are insufficient to explore the neuroscientific principles of visual quality perception.

Unlike the aforementioned EEG-related studies, this research employs fMRI in a no-reference experimental design, which more accurately reflects normal perceptual processes without access to the original signal. The no-reference experimental design also avoids the confounding effects associated with the full-reference paradigm, ensuring that the focus of the experiment is on visual quality itself rather than other factors. To better align with everyday perceptual scenarios, authentically distorted images are used to deeply explore the principles of visual quality perception in the human brain and the mechanisms involved in processing images of varying quality levels. We employ univariate analysis, seed-based functional connectivity analysis, and representational similarity analysis (RSA), build the prediction models between regions of interest (ROIs), and decode visual quality from fMRI data to comprehensively analyze the collected fMRI data.

Our experiments reveal that visual quality information is not explicitly encoded within any single ROI. However, it significantly impacts semantic encoding in primary visual areas while having minimal influence on higher-level visual regions. Although visual quality information is not directly encoded in individual ROIs, it is reflected in the predictive models of patterns between ROIs, as significant differences are observed in these models under varying quality conditions. When perceiving low-quality visual signals, an adaptive compensation mechanism within the visual pathway offsets information loss to ensure an accurate interpretation of the visual input. Leveraging the principle that low-quality signals evoke the compensatory mechanism, we decode visual quality information from fMRI signals and propose an artificial neural network multi-layer feature fusion strategy, which effectively enhances the performance in visual quality prediction.

The compensatory mechanism in the visual path proves that the brain leverages complex cognitive processes to adapt degraded visual inputs, ensuring that perception remains effective even in suboptimal conditions. To our knowledge, this study is the first to explore the mechanism of visual quality perception using fMRI. This study enriches our understanding of the human visual system and provides a physiological and psychological theoretical basis for the design of brain-inspired artificial visual systems and quality assessment models based on physiological data.

## Results

### Overview

Our goal is to explore the perception mechanism of visual quality and to identify the differences in activation levels and response patterns of various visual regions in the human brain when processing low-quality and high-quality images. To this end, we conduct an fMRI experiment in which 14 participants view natural images of three visual quality levels (low, neutral, and high quality) and three content categories (face, object, and scene) and perform quality assessment (QA) and content classification (CC) tasks. Each task condition includes 144 unique images (16 per category across nine categories), with each image presented twice for each participant.

All images are selected from KonIQ-10k, an image quality assessment (IQA) database including images with authentic distortion and MOS for their visual quality.^42^ We systematically categorized visual stimuli into three quality levels based on perceptual and semantic discriminability: low-quality images containing prominent global distortions that substantially impair semantic interpretation; neutral-quality images exhibiting localized distortions that preserve overall semantic recognition while compromising fine details; and high-quality images maintaining minimal perceptible distortions with sharp details, vivid colors, and unambiguous semantic clarity to serve as the perceptual reference standard. For details, see methods and Figure S1.

**Figure 1:**
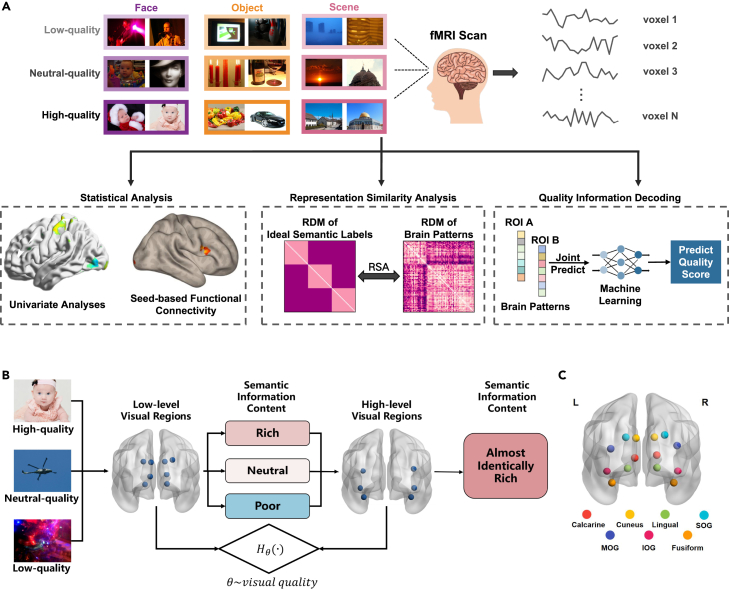
Overview (A) fMRI signals are collected from participants observing images of varying quality (see also Figure S1). Statistical analyses, including univariate and functional connectivity analyses, are first used to identify brain regions associated with quality perception. RSA is then applied to examine the quality information and semantic information within ROIs, and visual quality information is subsequently decoded from the fMRI data. (B) The brain’s processing mechanisms for images of varying visual quality. The semantic information content within the response patterns of low-level visual cortices (as marked in the figure, including bilateral calcarine, cuneus, lingual, and superior occipital gyrus) varies with image quality, decreasing as quality diminishes. Conversely, in high-level visual cortices (including bilateral middle occipital gyrus, inferior occipital gyrus, and fusiform gyrus), the semantic information content remains comparably rich across images of different qualities. This observation leads to the hypothesis that the information processing and transfer mechanisms between low-level and high-level visual regions are correlated with visual quality. (C) Visual depiction of the ROIs considered for analysis in the brain.

Blood oxygenation level dependent (BOLD) activity refers to changes in MRI signal caused by variations in blood oxygen levels within the brain. It reflects neural activity indirectly, as increased neuronal firing leads to localized increases in oxygenated blood, which alter the magnetic properties detected by fMRI.^43^ We analyze fMRI BOLD data using multiple methods, as shown in Figure 1A. First, we employ univariate analysis and functional connectivity analysis to demonstrate that quality assessment is more complex than content classification and prove differences in the activation patterns of visual regions when understanding low-quality and high-quality images. The RSA then shows that visual regions of the brain do not encode visual quality directly, but visual quality significantly impacts the semantic encoding in related brain regions. Moreover, when understanding low-quality images, visual-related brain regions compensate for the loss of semantic information in the images. Subsequently, prediction models between the response patterns of ROIs are established, proving that the mapping relationship between ROI response patterns varies with visual quality and identifying brain regions most significantly affected by image distortion and those compensating for semantic information loss in low-quality images. To validate that low-quality signals trigger compensatory mechanisms and lead to differences in pattern prediction models between ROIs, we decode visual quality using combinations of different ROIs, demonstrating the feasibility of decoding visual quality from fMRI data and identifying the optimal brain region combinations for representing visual quality. Finally, based on the conclusions drawn from the fMRI experiment, we propose an artificial neural network multi-layer feature fusion strategy that significantly improves the performance in visual quality prediction.

### Statistical analyses

**Figure 2:**
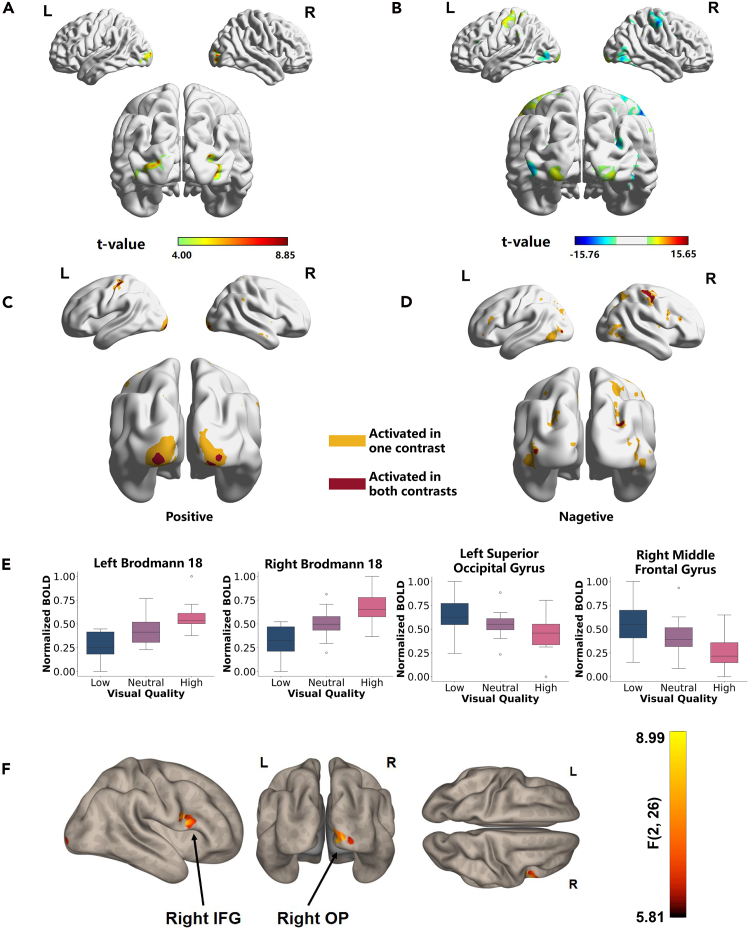
Statistical analysis results (A) The effects of task condition on BOLD activity. The brain regions showing increased and decreased activities during the QA task compared with the CC task. (B) The effects of task and quality condition on BOLD activity. Brain regions show increased and decreased activity when viewing high-quality images compared to low-quality images. Significance threshold p<0.001, FDR corrected at voxel level (p<0.05). (C and D) Brain regions demonstrating significant activation across both “high-quality vs. neutral-quality” and “neutral-quality vs. low-quality” contrasts. Regions of positive and negative activations under the two distinct contrasts are shown in (C) and (D), respectively. Yellow highlights brain regions that exhibit activation solely within one contrast, whereas magenta represents regions showing consistent activation across both contrasts. The criterion for statistical significance, denoting activation, has been conservatively established at a threshold level of p<0.001, FDR corrected at voxel level (p<0.05). (E) The mean of BOLD signal intensity within subregions of the bilateral BA18, right superior occipital gyrus, and right middle frontal gyrus across different image-quality levels for each participant. Error bars represent the standard deviation of BOLD signal intensity across participants. The vertical axis in these plots has been normalized to allow comparison, with the specific boundary of these subregions derived from the common activation regions identified in (C). (F) Parametric seed-based functional connectivity analysis results. Bilateral superior occipital gyrus serves as the seed, with significance thresholds set at voxel-level p<0.01 and cluster level p<0.05 for “low-quality < high-quality” conditions. Significant positive correlations are evident in two clusters located in the right Inferior frontal gyrus (IFG) and right occipital pole (OP), marked by arrows. *F* values range from 5.81 to 8.99, denoting the strength of connectivity.

To unravel the brain mechanisms underlying IQA, we first perform univariate analyses to explore the brain regions activated in the quality-assessment task. We focus on which brain regions are more sensitive in the quality-assessment task in contrast to the usual high-level task focusing on image semantics, so the contrast “QA vs. CC” is conducted, as shown in Figure 2A. We address the statistics and results in Table S1. In particular, we observe significantly increased BOLD activity in the visual pathway, such as the middle occipital gyrus, lingual, fusiform, and cuneus in the QA task compared to the CC task. However, increased activity in the CC task compared to the QA task is not found. To explore the physiological basis of quality of experience, we focus on whether images of varying quality would activate distinct brain regions. The contrast “high quality vs. low quality” is conducted, and its statistical results are detailed in Table S2. Both quality levels activate the precentral gyrus (left for low quality, right for high quality) according to the button placement for ratings. Similar activation patterns are observed in the insula, with high- and low-quality images activating the right and left insula.

In contrast “high-quality > low-quality” subregions of the lingual gyrus, inferior occipital gyrus, and middle occipital gyrus respond at a higher level to high-quality images, mainly involving regions Brodmann area 17 (BA17) and BA18. In addition, significant activation can also be observed in the bilateral inferior parietal lobule and bilateral cingulate gyrus, as shown in Figure 2B. In contrast “low quality > high quality,” significant activations can be observed in the subregion of bilateral fusiform, middle occipital gyrus, and superior occipital gyrus, mainly involving regions BA19 and BA37. Other activations are found in the bilateral middle temporal gyrus, inferior temporal gyrus, inferior frontal gyrus, middle frontal gyrus, left superior parietal lobule, and inferior parietal lobule.

Given the responsiveness of specific brain regions to stimulus quality, our study investigates regions showing positive or negative correlations with BOLD signal intensity related to quality. We conduct the contrasts “high quality vs. neutral quality” and “neutral quality vs. low quality,” identifying overlapping regions consistently activated across both contrasts. We scrutinize the beta parameters estimated by the general linear model (GLM) within these overlapping regions across high-, neutral-, and low-quality conditions. Results shown in Figure 2C indicate increased BOLD responses in bilateral BA18 subregions with higher image quality. By contrast, regions within the superior occipital gyrus and middle frontal gyrus show the opposite trend, with lower quality enhancing BOLD signal responsiveness, as shown in Figures 2D and 2E.

The univariate analysis above shows that the response amplitude in the superior occipital gyrus increases as the image quality degrades, which underscores the crucial role of the superior occipital gyrus in understanding low-quality images.

To further investigate the activity of the superior occipital gyrus under low-quality conditions, we conduct a seed-based functional connectivity analysis using the superior occipital gyrus as the seed. The results, as illustrated in Figure 2F, indicate that in the comparison of “low quality < high quality,” two clusters show significant positive connectivity with the superior occipital gyrus, located at the right occipital pole and the right inferior frontal gyrus (voxel threshold p<0.01, cluster threshold p<0.05). This suggests that under low-quality conditions, the superior occipital gyrus enhances cooperation with brain regions associated with advanced visual cognition, memory, and scene construction to better parse and understand the content of low-quality images.

### Representation similarity analysis

To analyze the response patterns of each visual region under different quality conditions, RSA and t-distributed stochastic neighbor embedding (t-SNE) are employed, which are generally accepted in neuroscience for multi-voxel pattern analysis.^44^ We aim to identify brain regions that encode visual quality, similar to how the fusiform face region responds to faces.^45^^,^^46^ If no such region is found, we will examine differences in response patterns across visual regions under varying quality conditions.

### Representations in visual regions for images of different qualities

Voxel-level BOLD activity patterns are extracted from each ROI for each presented image, with beta maps averaged across multiple presentations. This analysis combines data from all tasks and images without distinguishing between tasks. For each participant (number of participants *N* = 14), representation vectors are constructed using beta values from the 100 most reliable voxels in each ROI, including the calcarine, cuneus, lingual gyrus, fusiform gyrus, and superior, middle, and inferior occipital gyrus in both hemispheres. Vectors from the left and right hemispheres are concatenated for unified representation. The correlation distance between vectors for all images is calculated to create a representation dissimilarity matrix (RDM) for each ROI.

The t-SNE algorithm embeds high-dimensional data into 2D space, modeling similar data as near points and divergent data as far points, thus preserving similarity relationships. To visualize the similarity between representation vectors of images with different semantics under varying quality conditions, we calculate average RDMs across all participants for each quality level and use t-SNE to visualize these RDMs. See the methods section for details about the experiment setup.

**Figure 3:**
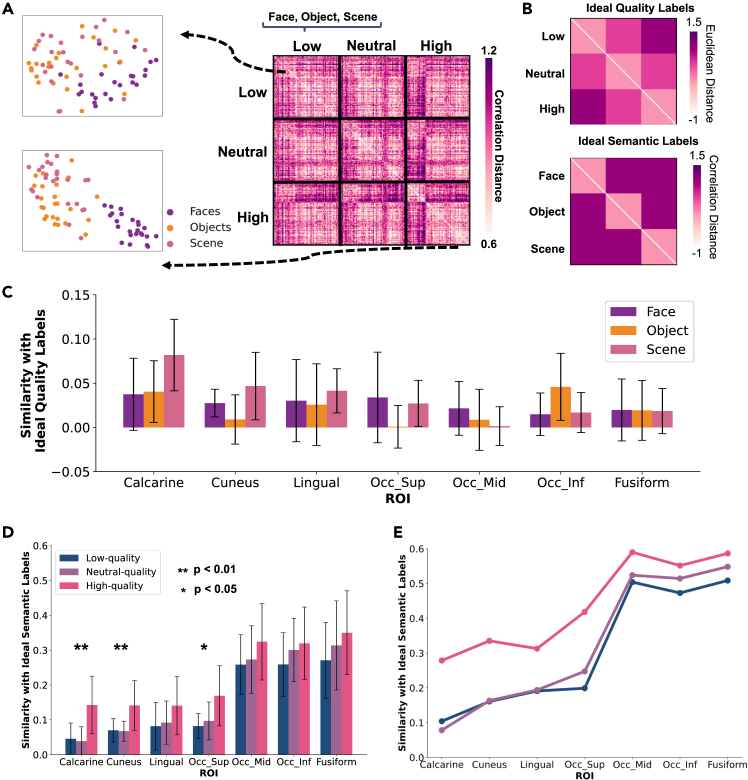
Representational similarity analysis: quantifying the semantic information content within each ROI (A) RDM and t-SNE in superior occipital gyrus. On the right, the RDM of the superior occipital gyrus is displayed, organized by quality levels (low, neutral, high) and within each quality level by semantic category (face, object, scene). All RDMs in this approach are constructed by calculating the pairwise correlation distances between fMRI response patterns across all trials. On the left, the results of dimensionality reduction via the t-SNE algorithm for RDMs under low- and high-quality conditions are visualized, with image content categories used as labels. (B) Ideal quality RDM and ideal semantic RDM. Here, it is assumed that the label for neutral quality is equidistant from those of low quality and high quality, and semantic labels are orthogonal to each other, represented by one-hot vectors. (C) The mean of quality information content within each ROI across participants. Error bars represent the standard deviation across participants. No single ROI contains a rich and explicitly encoded representation of visual quality information. (D) The mean of semantic information content within each ROI across low-, neutral-, and high-quality conditions across participants. Error bars represent the standard deviation across participants. Significant differences in the distribution of semantic information content across different quality conditions within each ROI are denoted with asterisks: ∗∗p<0.01, ∗p<0.05, and no asterisk indicates no significant difference in semantic information content across quality conditions within that ROI. (E) The average similarity between the RDMs of all participants and the standard semantic RDM. The average result reflects the overall consistency in representing semantic content across the sample.

Figure 3A shows the average RDM and the t-SNE results for the superior occipital gyrus under high- and low-quality conditions. The RDM demonstrates that representation vectors of semantically congruent images form discernible clusters in the t-SNE-derived 2D space, particularly for facial stimuli within the superior occipital gyrus. However, no analogous spatial clustering is observed for vectors representing quality-matched images in any examined brain region. This suggests that, unlike semantic information, visual quality attributes are not robustly encoded in the spatial patterning of neural responses within these regions. For full t-SNE results across quality conditions, see Figure S4.

Figure 3A also reveals that in low-quality conditions, the clustering of representation vectors of the images with similar semantics in the superior occipital gyrus is less distinct than in high-quality conditions, suggesting more disorganized semantic encoding. RDMs and t-SNE results for other ROIs are available in Figure S3.

### Quantifying the content of semantic information in the representation vectors of each visual region

To quantitatively analyze the impact of visual quality on response patterns in visual brain regions, we quantify the quality and semantic information content in representation vectors of each ROI. RDMs are computed for each brain region of each participant in three quality conditions, and their similarities to the ideal quality and semantic RDMs are measured using Pearson correlation coefficients, as shown in Figure 3B.

We first analyze the information content of visual quality within each brain region, with results shown in Figure 3C. The analysis reveals low-quality-related information content across all examined brain regions, with no regions explicitly encoding quality information. In contrast, semantic information content is more substantial across brain regions and varies under different quality conditions. As shown in Figure 3D, the content of semantic information is richer in high-level visual regions, consistent with the human brain’s ventral visual stream. Specifically, the semantic content in the calcarine, cuneus, and superior occipital gyrus shows a significant correlation with visual quality, with lower quality leading to reduced semantic content (Welch’s ANOVA: calcarine F(2,24.65)=8.32,p=0.0017; cuneus F(2,24.14)=6.01,p=0.0076; superior occipital gyrus F(2,23.68)=5.44,p=0.0114). As information moves to higher visual regions, these quality-induced differences become non-significant statistically. In the middle occipital gyrus, inferior occipital gyrus, and fusiform gyrus, semantic content remains statistically unaffected by quality. The power analysis is shown in Table S4 and the section “supplemental statistical power analysis” in supplemental notes.

The average RDMs of each ROI across participants in each quality condition are computed and their similarities to ideal semantic labels measured. As shown in Figure 3E, averaging reduces random noise and individual differences, leading to more robust results. All ROIs show higher semantic content across all quality conditions than individual analyses. The difference in semantic information content is largest between high- and low-quality conditions in the calcarine, decreases between the superior and middle occipital gyrus, and remains low in the middle occipital gyrus, inferior occipital gyrus, and fusiform.

Noise and information loss in low-quality images impact response patterns in primary and secondary visual regions, weakening cognitive functions in image recognition, while high-level visual regions continue to encode semantic information effectively from low-quality images with information loss. This indicates that there are compensatory mechanisms within the visual pathways that address the loss of information in low-quality visual signals, correctly encoding semantic information to ensure proper image understanding.

### Building the response pattern prediction model between ROIs

As illustrated in the section “representation similarity analysis,” the semantic content in primary visual regions decreases as visual quality degrades, while it remains largely unaffected in high-level visual regions. Based on the above observations, we hypothesize that the intermediate part of the ventral visual pathway is actively adaptive to low-quality conditions, leading to changes in the way information is processed and transmitted between the primary visual regions and high-level visual regions. Consequently, the mappings between response patterns in different ROIs of visual pathways exhibit differences in response to varying qualities.

To validate the hypothesis and identify brain regions with adaptability to low-quality conditions, we implement a system to model the predictive relationship between response patterns from different brain regions under three quality conditions: high quality, neutral quality, and low quality. Specifically, each ROI is used to predict the response patterns of the ROIs with higher levels in the visual processing pathway, learning the mapping relationships and analyzing their dependency on visual quality.

Let X be the representation vector of images in ROI A, and Y be the representation vector in ROI B. There exists a mapping system H(·) such that Y=H(X). Should our hypothesis be supported, we expect to identify the ROI pairs [A,B] for which the mapping system H(·) is not invariant to visual quality, i.e.,

$$Y=H_{\theta }\left(X\right)\text{,}$$

where θ∼visualquality.

Initially, images and their corresponding representation vectors under high-quality, neutral-quality, and low-quality conditions are divided into training and testing sets. A support vector regression (SVR) model is then trained using the high-quality training set to establish a mapping model between the representation vectors of ROIs A and B. This model’s performance is subsequently tested on the test sets of the three different quality conditions. Performance is quantified by the linear correlation between the predicted and the true representation vectors of ROI B. To mitigate the impact of shared signals between brain regions, we employ cross-prediction using data from two repeated measurements. The noise ceilings are estimated by calculating the linear correlation between the response patterns of each ROI across the two measurements.

Significant performance discrepancies should emerge across test sets of different qualities if the mapping between ROIs A and B varies with quality. Furthermore, the system’s performance on the test set corresponding to the low-quality training condition may exceed that under other conditions, suggesting a particular adaptability to low-quality images.

**Figure 4:**
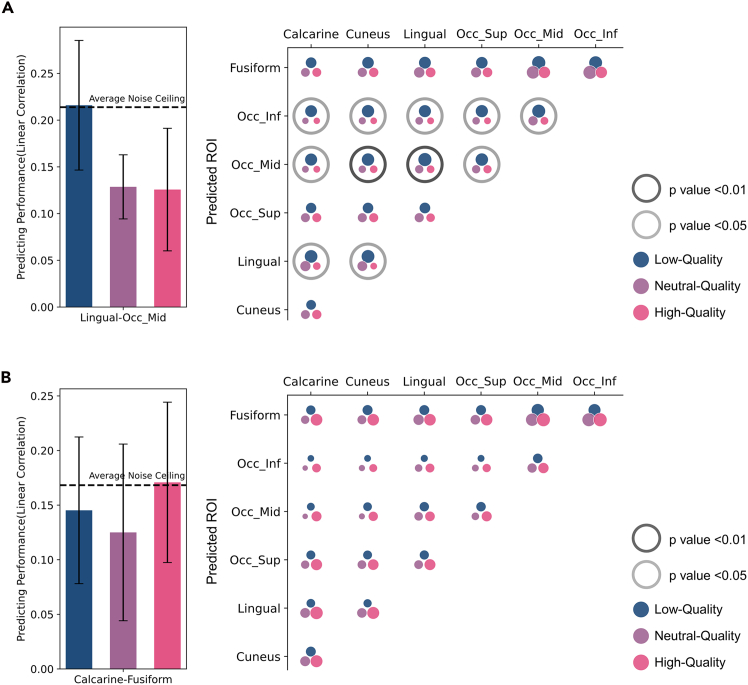
Building the response pattern prediction model between ROIs (A) Test results of high-, neutral-, and low-quality data on SVR models trained using low-quality response patterns. On the left, the mean of predictive performance of the model predicting middle occipital gyrus from lingual among participants is displayed. Error bars represent the standard deviation across participants. On the right, the prediction results between any two ROIs (predicting the pattern of the ROI on the *y* axis from the patterns of ROI on the *x* axis) are shown, where bubble size indicates the mean test performance among participants. Highlighted clusters indicate significant variability in model performance across different quality conditions on the corresponding test sets (Welch’s ANOVA, significance threshold p<0.05, with specific levels of significance indicated in the legend). (B) Test results of high-, neutral-, and low-quality data on SVR models trained using high-quality response patterns. On the left, the mean of predictive performance of the model predicting fusiform from the calcarine among participants is presented. Error bars represent the standard deviation across participants. The cluster shows that significant variability in model test performance across different quality conditions is not found.

The experiment is conducted independently for each participant, using ROI representation vectors consistent with the previous RSA. Seven ROIs (calcarine, cuneus, lingual, fusiform gyrus, and superior, middle, and inferior occipital gyrus) are paired to make predictions from *A* to *B*. Low-quality training datasets are used to train the SVR model, mapping representation vectors between ROIs *A* and *B* using an approach similar to that described above. A model adapted to low-quality images would perform best on low-quality tests. As shown in Figure 4A, the predictive performance of the model predicting the middle occipital gyrus and the inferior occipital gyrus from the other ROIs, as well as the model predicting the lingual from the cuneus, is significantly correlated with quality conditions, with significantly better performance on low-quality tests than on neutral or high-quality ones. However, after training under high-quality conditions, we did not identify any ROI pairs with significantly higher performance on the high-quality test set, as shown in Figure 4B. Detailed data for each ROI pair can be found in Figures S6 and S7. The power analysis is shown in Table S5 and the section “supplemental statistical power analysis” in supplemental notes.

This result is consistent with the well-known free energy principle (FEP), which posits that the brain processes information by generating predictions about sensory inputs and minimizing prediction errors.^47^^,^^48^ Our research provides a concrete example supporting this theory. The findings presented in “representation similarity analysis” demonstrate that the brain can ensure semantic understanding even under conditions of low-quality visual signals. The phenomenon in this section indicates that the brain continuously refines its predictions to reduce uncertainty, in line with the error minimization principle proposed by the FEP. Furthermore, this experiment illustrates how the brain adjusts its internal models to adapt to changing environmental conditions, a behavior anticipated by the FEP.

### Predicting visual quality using response patterns of ROI pairs

According to the results in “representation similarity analysis,” we find that individual ROI response patterns contain minimal visual quality information. However, a compensatory mechanism within the visual processing pathway ensures that low-quality visual signals are adequately interpreted, and the prediction models between ROIs exhibit variations depending on visual quality, as illustrated in the section “building the response pattern prediction model between ROIs.” Therefore, we have reason to hypothesize that, although no brain region explicitly encodes visual quality information, it is feasible to decode visual quality by combining information from multiple ROIs. To validate this, we predict visual quality from fMRI signals using representation vectors of individual ROIs and concatenate vectors from pairs of seven vision-related ROIs, as shown in Figure 4B. We also concatenate vectors from all seven ROIs, reflecting the joint predictive performance of all ROIs. Randomly generated predictions for visual quality demonstrate the difference between predictions from brain regions and random results. Experiments use the same vectors as in RSA analysis, conducted independently on each participant’s data, maintaining consistent visual quality labels. SVR models are used with four-fold cross-validation and repeated ten times to minimize random effects on conclusions.

Predictive performance is measured using Spearman’s rank correlation coefficient (SRCC) and Pearson’s linear correlation coefficient (PLCC). SRCC primarily reflects the consistency and monotonic relationship between the predicted visual quality ranking and the ground truth, while PLCC primarily reflects the linear correlation between predicted visual quality and ground truth. Higher SRCC and PLCC indicate greater consistency with human subjective perception of visual quality. The mean and variance of performance across all participants are reported. We use a two-tailed independent-samples t test to analyze whether there is a significant difference in the mean predictive performance of each brain region or ROI pair compared to the joint predictive performance of all ROIs across all participants (threshold p<0.1, false discovery rate [FDR] corrected).

**Figure 5:**
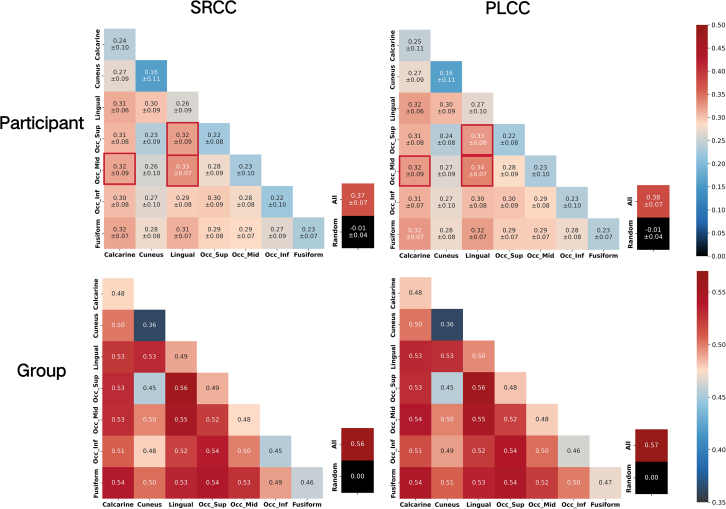
Predicting visual quality using response patterns of ROI pairs Single-participant quality information decoding results include the mean and standard deviation among participants of the SRCC and PLCC between the regression-predicted quality scores from the combined representation vectors of the ROIs (as indicated on the *x* axis and *y* axis) and the ground truth. Higher SRCC and PLCC values indicate greater consistency between the predicted quality information and the quality labels. The term “All” refers to the performance of combined predictive models using representation vectors from all seven ROIs. “Random” represents the SRCC or PLCC between randomly generated quality scores and the ground truth. A red box indicates that there is no significant difference in the mean performance among participants between the combined prediction of that particular ROI pair and the combined prediction using “All” seven ROIs (analyzed using a two-tailed independent samples t test, threshold p<0.1, FDR corrected). Group-level quality information decoding results include the mean of all participants’ predicted scores, which is used as the group prediction outcome.

The experimental results, as illustrated in Figure 5, show that joint prediction using all ROIs achieves the best performance. Among them, the prediction results of a single ROI are generally lower than those of the joint prediction using two ROIs. The ROI pair lingual-middle occipital gyrus achieves the best performance among all ROI pairs, and ROI pairs including calcarine and lingual generally have high prediction performance. There are some ROI pairs, such as calcarine-middle occipital gyrus, lingual-superior occipital gyrus, and lingual-middle occipital gyrus, whose joint prediction performance is not significantly different from using all ROIs for joint prediction.

We calculate the SRCC and PLCC between the ground truth and the average of all participants’ predictive results, designated as the group prediction performance. The results, illustrated in Figure 5, demonstrate that the group prediction significantly surpasses individual predictions. This improvement is likely due to the attenuation of random noise effects. Furthermore, the discrepancy between the predictive performance of individual ROI pairs and the combined predictions using all ROIs is notably reduced. The best-performing ROI pair in the group prediction is the lingual-superior occipital gyrus and lingual-middle occipital gyrus, which coincides with the dominant ROI pair identified in the above participant-level predictions.

The results in Figure 5, both for individual and group predictions, show that decoding MOS from participants’ fMRI signals performs worse than existing deep-learning-based IQA algorithms, which predict visual quality from images. We attribute this phenomenon to two main factors. First, the fMRI signal inherently contains considerable noise and physiological variability, whose substantial influence is widely acknowledged in many fMRI decoding studies.^23^^,^^49^^,^^50^ Second, while MOS is an explicit quantification of human subjective experience, it may not fully capture variations in user perception. This issue is also commonly observed in studies using EEG to measure visual quality perception.^38^^,^^41^ The decoding performance of each participant can be found in Figure S14 and Tables S6 and S7.

### Brain-inspired multi-layer feature fusion strategy for artificial neural networks

It has become common practice in recent human fMRI research to consider convolutional neural networks (CNNs) as computational models of the human visual system. This view is supported by fMRI studies demonstrating that representations in the lower and higher layers of CNNs correspond to neural activity patterns in the early and higher-level visual processing regions of the brain, respectively.^25^^,^^26^^,^^51^^,^^52^ In particular, object category encoding learned by deep convolutional networks trained on object classification tasks serves as a robust proxy for neural representations.^26^

As demonstrated in “representation similarity analysis,” visual quality information is not directly encoded within a single ROI. In “building the response pattern prediction model between ROIs,” we prove that the prediction models between ROIs vary with visual quality, indicating that quality information is reflected in the relationships among ROIs. Based on this discovery, we successfully decode visual quality information from fMRI signals in the section “predicting visual quality using response patterns of ROI pairs.” The results indicate that the combined prediction performance of two or more ROIs is better than that of a single ROI. This implies that different visual regions interact and collaborate during the processing of visual quality. Consequently, we propose a multi-layer fusion strategy to mimic this process in the CNN pre-trained on object classification tasks, aiming to explore whether the findings from fMRI experiments can be validated in CNNs.

**Figure 6:**
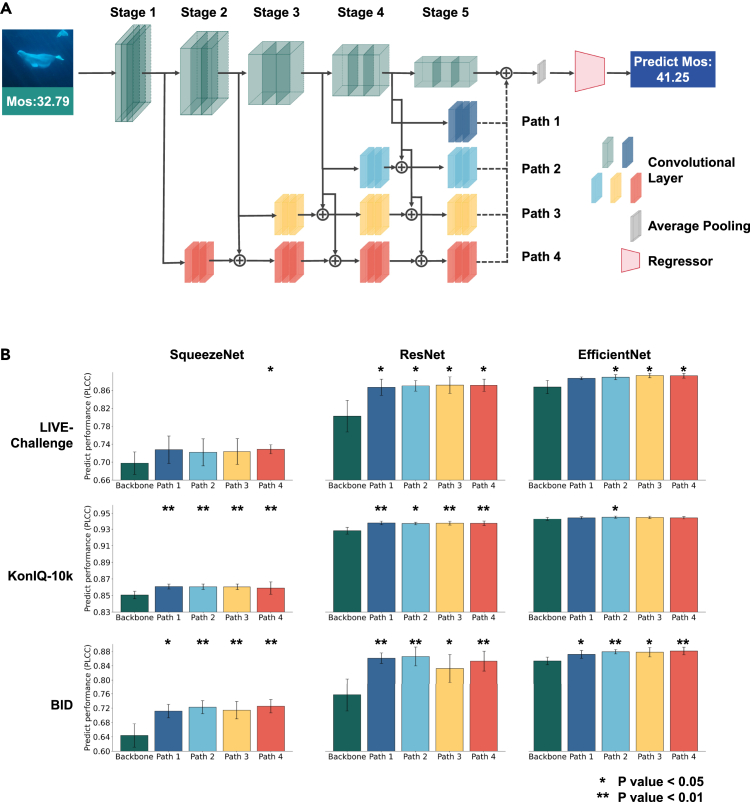
Brain-inspired multi-layer feature fusion strategy for artificial neural networks (A) Diagram of the proposed artificial neural network multi-layer feature fusion strategy for visual quality prediction. The model uses a convolutional neural network (CNN) backbone to extract features across five stages (stages 1–5). Four independent paths (paths 1–4) branch out to capture multi-scale features at different stages. Each path uses convolutional layers to achieve dimension matching with the next stage, with the outputs fused for predicting the MOS. This architecture leverages both low-level and high-level features to enhance the accuracy of quality prediction. (B) Performance comparison of the backbone and multi-path configurations (paths 1–4) across three benchmark datasets, namely LIVE-Challenge, KonIQ-10k, and BID, using different backbone architectures (SqueezeNet, ResNet, and EfficientNet). The PLCC values demonstrate the consistent improvement achieved by integrating features from paths 1–4, with significant performance gains over the backbone alone, indicating the effectiveness of multi-scale feature integration in visual quality prediction. Statistical significance is marked as ∗*p* < 0.05 and ∗∗*p* < 0.01. The error bars represent the standard deviation from five-fold cross-validation.

The architecture shown in Figure 6A incorporates a backbone network for feature extraction and additional paths (paths 1–4) that branch out from intermediate layers to capture multi-scale features and fuse them at the regression head. It aims to integrate information from different levels, similar to how the brain regions work together in the visual pathway. This strategy is expected to improve the performance of visual quality prediction by leveraging the unique contributions of features at different levels, just as the brain utilizes the cooperation of different visual regions for effective VQA. We selected SqueezeNet,^53^ ResNet,^54^ and EfficientNet^55^ as backbone networks to evaluate the generalizability and adaptability of our proposed feature fusion method across architectures with varying complexity and design principles. SqueezeNet represents a lightweight model optimized for efficiency with significantly fewer parameters, making it suitable for deployment-constrained environments.^53^ ResNet introduces residual learning, which has proven effective in stabilizing deep network training and improving performance across a wide range of vision tasks.^54^ EfficientNet, on the other hand, leverages a compound scaling strategy to achieve high accuracy with balanced computational cost.^55^ The inclusion of these three models allows us to assess the robustness of our fusion strategy under different architectural paradigms. For details of the network architecture and parameters, refer to the methods section.

The backbone networks are initially pre-trained on ImageNet,^56^ a widely used large-scale benchmark dataset in image classification that enables the acquisition of robust visual semantic representations exhibiting similarities to human visual cortex processing.^27^ Subsequently, these networks are fine-tuned on each IQA dataset to adapt specifically to the characteristics of IQA. The experiments use a five-fold cross-validation scheme across three authentic distortion IQA benchmark datasets: LIVE-Challenge (CLIVE),^57^ KonIQ-10k,^42^ and BID.^58^ These datasets are selected to balance content diversity, distortion types, and annotation quality, ensuring a thorough validation of the network’s generalizability and performance. For detailed information on dataset sizes, distortion types, and other experimental settings, please refer to the methods section.

Each path (paths 1–4) and the backbone are independently trained and tested while maintaining identical dataset splits, training protocols, and hyperparameters to ensure a fair comparison of predictive performance. As shown in Figures 6B and S8, the results suggest that incorporating features from path 2, path 3, and path 4 generally leads to improved prediction performance (measured by PLCC and SRCC) compared to using the backbone alone. While the differences in correlation are relatively small, path 2 shows a consistent advantage on the KonIQ-10k and BID datasets, potentially benefiting from the high-level semantic information it encodes. In addition, path 3 and path 4, which leverage intermediate and lower-layer features, perform comparatively well on CLIVE, indicating potential robustness to complex quality variations. These findings imply that both high-level and low-level features may provide complementary information for quality prediction.

The statistical significance tests confirm the effectiveness of the fusion strategy, with path 2, path 3, and path 4 providing consistently strong and reliable results across different datasets and backbones. This indicates that features extracted at different levels of the network contribute uniquely to understanding visual quality. Integrating features from different levels can enhance the network’s performance in predicting visual quality. Furthermore, we compare the prediction performance of the network with multi-layer feature fusion to other IQA methods. As shown in Table 1, EfficientNet with the multi-layer feature fusion strategy demonstrates superior visual quality prediction performance. This result indicates that the multi-layer feature fusion strategy not only validates the conclusions of the previous fMRI experiment in CNNs but also holds practical application value.

**Table 1:** Performance comparison of models on different IQA datasets

Model	CLIVE		KonIQ-10k		BID	
	SRCC	PLCC	SRCC	PLCC	SRCC	PLCC
DPCS^59^	0.8560	0.8730	0.9090	0.9140	–	–
CNNIQA^60^	0.6269	0.6008	0.6852	0.6837	0.6163	0.6144
WaDIQaM-NR^61^	0.6916	0.7304	0.7294	0.7538	0.6526	0.6359
SFA^62^	0.8037	0.8213	0.8882	0.8966	0.8202	0.8253
DB-CNN^15^	0.8443	0.8624	0.8780	0.8867	0.8450	0.8590
HyperIQA^63^	0.8546	0.8709	0.9075	0.9205	0.8544	0.8585
Ours: SqueezeNet path 4	0.6894	0.7286	0.8364	0.8589	0.7157	0.7252
Ours: ResNet path 4	0.8470	0.8712	0.9232	0.9371	0.8412	0.8529
Ours: EfficientNet path 4	**0**.**8731**	**0**.**8924**	**0**.**9306**	**0**.**9446**	**0**.**8611**	**0**.**8812**

The highest performances are highlighted in bold, while the second highest are indicated with underlining.

From the results, it can be observed that the multi-layer feature fusion strategy exhibits varying performance across the three networks and datasets. A detailed analysis of the reasons behind the differences can be found in the discussion.

To validate the generalizability of the multi-layer models across datasets with different sources, we evaluate the models on the CLIVE and BID datasets, which are pre-trained on KonIQ-10k. The KonIQ-10k dataset, being larger in scale compared to CLIVE and BID, is more suitable for pre-training purposes. As shown in Figures S10 and S11, the results demonstrate that incorporating features from path 2, path 3, and path 4 consistently improves prediction performance (measured by PLCC and SRCC) compared to the backbone alone. The statistical significance tests confirm the effectiveness of the fusion strategy in enhancing the generalizability across datasets with different sources. For detailed training procedures and hyperparameters, please refer to the methods section. The loss curve for each network on each dataset can be found in Figure S9.

## Discussion

We use fMRI to investigate the principles of visual quality perception and image-processing mechanisms in the human brain in a no-reference design with authentically distorted images. During scanning, participants’ QA and CC task response is recorded via button presses. Detailed button analysis appears in the section “supplemental button-press data” of supplemental notes. Results show that even the same participant may produce inconsistent judgments for identical stimuli. Experienced image-quality assessors exhibit higher behavioral consistency in rating tasks, as shown in Table S3. Despite this, fMRI-based visual quality decoding performance does not differ significantly between experienced and inexperienced participants, as shown in Figure S13 and Table S3. Thus, all fMRI analyses here pool data across both groups. This finding underscores the work’s necessity: subjective ratings are influenced by intra-individual variability, while physiological signals reflect visual distortion more objectively and directly. Exploring quality perception mechanisms via fMRI guides addressing limitations of traditional rating paradigms in quality assessment.

The results in the section “statistical analyses” indicate that the QA task, compared to the CC task, involves stronger and more extensive brain activation, suggesting that the quality-assessment task is more complex and demanding than semantic categorization. Although IQA is generally seen as a low-level task,^64^^,^^65^ and semantic recognition, like image classification, as a high-level task, our results show that quality assessment activates not only bilateral visual pathways (e.g., lingual, fusiform, cuneus, and inferior temporal gyrus) but also regions related to high-level cognitive functions, such as the inferior frontal gyrus (Figure 1A).

The visual associative pathways are essential for processing complex visual information, executing goal-directed tasks, and managing visual attention. This implies that IQA requires a more detailed analysis of fine-grained attributes such as detail, clarity, color accuracy, and contrast. The involvement of the inferior frontal gyrus suggests that quality assessment extends beyond a low-level task. It requires the inferior frontal gyrus for attention regulation, integration, and evaluation of information in complex situations and decision making.^66^^,^^67^

Analyzing the RDMs from various ROIs, we find that images with the same semantics cluster effectively, but images of the same quality do not elicit similar response patterns in any brain region. However, image quality does influence semantic representations within ROIs. Under low-quality conditions, primary visual regions show disorganized semantic encoding, resulting in less distinct clustering of semantically similar images, while this effect is reduced in higher-level visual regions. From these observations, we derive two conclusions.

First, visual quality affects the visual understanding process, mainly in low-level visual regions, supporting the classification of quality assessment as a low-level task in the field of computer vision.^64^^,^^65^ This influence primarily occurs during the low-level feature extraction phase, regardless of whether the brain is engaged in quality-assessment or content-classification tasks, explaining why response patterns in visual ROIs do not differ significantly between tasks. Quality assessment involves processing image semantics, and content classification is also affected by quality, indicating that these tasks are not entirely separate. Furthermore, quality assessment involves high-level cognitive regions working with visual associative pathways, highlighting that subjective quality ratings engage high-level cognitive functions, potentially introducing biases and missing deeper perceptual processes. This supports the need for using physiological signals like EEG under natural viewing conditions as objective indicators of human perception of quality, aligning with findings in other studies.^38^^,^^39^^,^^41^

Second, the strong impact of quality on semantic encoding in primary and secondary visual regions, which lessens in higher-level visual regions, suggests that the brain adapts to low-quality images, compensating for information loss along the visual pathway. This also explains a common phenomenon: we can often comprehend the semantic content of low-quality visual signals, even when these signals are distorted and have lost some information. This supports the idea that the ventral stream operates as a hierarchy of increasingly abstract processing stages.^68^^,^^69^^,^^70^ Observations show that under high-quality conditions, semantic information increases progressively from the lingual to the superior and middle occipital gyrus. In contrast, under low-quality conditions, this increase is not seen between the lingual and superior occipital gyrus but becomes significant between the superior and middle occipital gyrus, where quality effects diminish. This suggests that the superior occipital gyrus plays a key role in compensating for information loss in low-quality images.

In “building the response pattern prediction model between ROIs,” using SVR to establish mapping relationships between ROIs, we demonstrate that some inter-ROI mappings correlate with visual quality. In Figure 4A, it can be seen that when predicting the middle occipital gyrus and inferior occipital gyrus from the other ROIs, the predictive performance under low-quality conditions is significantly higher than under high-quality conditions. This difference may be due to the visual system’s specialized processing mechanism for low-quality information. Under this mechanism, the information processing and transfer methods between the mid-segment ROIs of the visual pathway are specifically adapted to low-quality information, hence the SVR model trained with low-quality data performs poorly on high-quality test data. This conclusion aligns with the findings in “representation similarity analysis.”

According to the FEP,^47^^,^^48^ the brain’s compensatory mechanisms aim to minimize the free energy between sensory inputs and predictions. When processing low-quality inputs, high-level visual regions engage more in updating internal models and using prior knowledge to predict possible scenes, even with incomplete or damaged information, thereby reducing prediction errors. Our findings not only support the FEP’s description of how the brain processes information but also specifically reveal how high-level visual regions deal with declines in sensory input quality through compensatory mechanisms. Before this, Zhai et al.^8^^,^^11^ and Gu et al.^8^^,^^11^ had already applied the FEP to VQA. According to these authors, psychovisual quality can be understood as a measure of the consistency between external visual input and the brain’s internal model.^8^^,^^11^ When the quality of the input image degrades, prediction errors increase, leading to higher free energy. Consequently, Zhai et al.^8^ achieved IQA by quantifying changes in free energy. Their metric performed exceptionally well across various types of image degradation, particularly excelling in conditions of blur and noise compared to other conventional metrics. Years later, our study employs fMRI technology to validate the physiological plausibility of the perspectives presented in these studies.^8^^,^^11^

The research highlights the critical role of the superior occipital gyrus in the ventral visual pathway, particularly in processing low-quality images. Figure 2E shows that superior occipital gyrus response amplitude increases as image quality degrades. Seed-based functional connectivity analysis reveals that the connectivity of the superior occipital gyrus with the occipital pole and inferior frontal gyrus adapts significantly to low-quality conditions (see Figure 2F). These regions collaborate to compensate for information loss in lower-quality images, maintaining visual perception and understanding. This analysis underscores the functional importance of the superior occipital gyrus and its interaction with other cortical regions in handling varying image qualities.

The enhanced connectivity between the superior occipital gyrus and the inferior frontal gyrus highlights the brain’s ability to integrate high-level cognitive functions with basic sensory processing. This connectivity suggests that when viewing low-quality images, the superior occipital gyrus not only intensifies basic visual processing but also engages the inferior frontal gyrus, which is involved in processing complex and ambiguous information as well as semantic processing and memory retrieval.^71^^,^^72^^,^^73^ This interaction reflects a compensatory approach, whereby the brain utilizes complex cognitive processes to interpret low-quality visual inputs, ensuring effective perception even under suboptimal conditions. This adaptability is vital for coherent perception, especially in tasks such as navigation and recognition, and offers valuable insights for designing natural and artificial visual systems.

For example, the human visual system’s adaptability to low-quality conditions distinguishes it from existing multimodal large language models (MLLMs), which follow a consistent inference process for each input. Research shows that MLLMs are generally sensitive to image perturbations like noise, blur, and distortions, significantly degrading performance.^36^ Training MLLMs with noisy images improves robustness to similar noise but often reduces performance on clean images due to overfitting noise features.^74^^,^^75^ This trade-off affects the model’s specificity and precision on clean data and complicates attention mechanisms. While MLLMs balance this by retaining generalizable knowledge through fine-tuning,^76^ the human visual system adapts to low-quality images without impairing its ability to process high-quality ones, and without specific training for low-quality conditions. Our findings on brain adaptation mechanisms offer valuable insights for enhancing MLLM’s robustness to noise.

These findings also align with cognitive load theory,^77^ showing that low-quality images increase activity and connectivity in high-level visual and cognitive regions, disrupting semantic encoding and raising cognitive load as the brain compensates for information loss. Poor visual quality adds extraneous cognitive load, causing learners to consume cognitive resources interpreting images, thus reducing resources for actual learning and complicating new information integration. This supports cognitive load theory, which posits that intrinsic cognitive load is linked to the complexity of learning materials and heavily occupies working memory, potentially reducing learning efficiency.^78^^,^^79^ This study highlights the impact of visual quality on cognitive load, offering insights for designing experiments on human learning processes.

Through fMRI studies, we found that different visual regions interact and collaborate in visual quality processing. Given the common practice in recent human fMRI research of using CNNs as computational models of the human visual system,^25^^,^^26^^,^^51^^,^^52^ we employ a multi-layer fusion strategy to mimic this process in CNNs pre-trained on object classification tasks, aiming to validate the fMRI findings in CNNs. Experimental results indicate that the multi-layer feature fusion strategy performs variably across the three networks and datasets. We analyze the reasons for this phenomenon as follows.

SqueezeNet, optimized for efficiency via 1 × 1 convolutions and fire modules, has limited fitting ability due to low representational capacity, leading to poor IQA performance. Our strategy improves its fitting power, significantly boosting results on all datasets. However, the improvement on CLIVE is less pronounced compared to other datasets, with paths 1–3 lacking statistical significance. This is primarily because CLIVE exhibits both a small dataset size and a wide variety of distortion types, which increase fitting difficulty and lead to distribution shifts across splits, causing unstable learning performance. Compared to paths 1–3, path 4 integrates features from all stages, providing more learnable modules and delivering more stable improvements for SqueezeNet under the most challenging conditions. In contrast, BID is small but has a single distortion type, while KonIQ-10k is large with diverse distortions. On both, our strategy yields stable performance gains for SqueezeNet.

ResNet uses residual connections to ease training in deep networks by addressing vanishing gradients. Its skip-connection structure also mirrors insect brain pathways at the micro level.^80^ Our strategy adds macro-level feature fusion, aligning well with ResNet’s architecture. ResNet50 balances fitting capacity and parameter count, showing strong baseline performance. With our strategy it achieves consistent, significant gains across all datasets and faster loss convergence on CLIVE and BID, as shown in Figure S9.

EfficientNet, with its compound scaling of depth, width, and resolution, offers strong fitting ability. On the large and diverse KonIQ-10k dataset, it achieves high baseline performance, leaving limited room for improvement with our fusion strategy. This explains the less significant gains observed. In contrast, the smaller CLIVE and BID datasets pose greater demands on feature extraction. Here, our strategy boosts EfficientNet’s performance and accelerates loss convergence on both the CLIVE and BID datasets, as shown in Figure S9.

In summary, the proposed strategy enhances feature extraction in ResNet and EfficientNet, leading to improved accuracy and faster convergence, especially on small datasets. However, its efficacy is limited when applied to SqueezeNet with a small parameter size; both the performance improvement and loss convergence speed brought by the strategy are less significant for SqueezeNet compared to ResNet and EfficientNet. Overall, the strategy is most effective for CNNs with moderate parameter counts on challenging, small-scale datasets but shows limited benefit for underpowered models or when baseline performance is already near optimal.

Given the limited research on visual quality in neuroscience, our experimental design is conservative. All participants view the same limited set of stimuli, making deep-learning approaches like those in BOLD5000^81^ or NSD^82^ unsuitable. Future work will categorize image degradation types, increase dataset size, and expand the dynamic range of visual quality. This will enable a deeper exploration of the brain’s cognitive responses to impaired visual signals and help identify physiological markers for no-reference quality-assessment tasks.

## Methods

### fMRI participants

Seventeen healthy right-handed university students participated in this study. Three participants were excluded for excessive motion, leaving 14 for analysis (seven males and seven females, ages 20–27, mean age 23.28, standard division 2.05). Among the 14 participants, four had extensive experience in VQA experiments. Excessive motion is defined a priori as >2 mm translation or >2° rotation. All participants completed a Snellen visual acuity test prior to the experiment and confirmed that they have normal or corrected-to-normal vision, defined as a Snellen acuity score of 20/20 at a distance of 6 m.^83^ All participants have no known neurological condition. The institutional review board at Shanghai Jiao Tong University approved the experimental protocol, and all participants gave written informed consent. After being informed about potential risks and screened by an institution’s physician, participants gave informed consent before participating. All data were processed anonymously.

### Stimuli

All images are manually selected from KonIQ-10k, an IQA database including images with authentic distortion and MOS for their visual quality.^42^ The KonIQ-10k contains 10,073 images selected from the large public multimedia database YFCC100m,^84^ covering a wide range of distortions in brightness, color, contrast, noise, sharpness, and other quality dimensions. The subjective scores in the KonIQ-10k dataset are collected through a crowdsourcing protocol, with each image rated by at least 120 unique participants. The ratings followed the standard 5-point absolute category rating scale: bad (1), poor (2), fair (3), good (4), and excellent (5). After quality control and filtering, the scores are averaged across participants, normalized to a [0–100] scale, and aligned with expert opinion to derive the MOS for each image. KonIQ-10k ensured content diversity by sampling 1 million images from YFCC100m based on machine-generated tags, which are assigned by a deep neural network and represented the top predicted categories for each image. When selecting stimulus images, visual quality is categorized based on the MOS provided by the dataset. Scores below 35 are classified as low quality, between 35 and 70 as neutral quality, and above 70 as high quality. All images are cropped to fit a 1,024×724-pixel frame. When classifying image content, we manually inspect each image to ensure that its semantic content is unambiguous and can be uniquely assigned to one of three categories: faces, objects, or scenes.

All stimuli are classified into nine categories according to content category (faces, objects, or scene) and image quality (bad, neutral, or excellent). These images are prepared for two tasks: IQA and content classification. Under each task condition, there are 16 images per category across nine categories, resulting in a total of 144 unique images. Each image is presented twice, leading to 288 trials per task for each participant.

### fMRI experiment procedure

The experimental process is divided into eight runs, each containing four blocks. Participants in each block perform one of the two tasks: quality assessment (bad, neutral, or excellent) or content classification (faces, objects, or scene), and each block contains 18 trials, as shown in Figure S2. A single continuous scanning run lasted 384 s and comprised four blocks, each lasting 96 s. Each block included an initial task instruction period (4 s), presentation of 18 images (3,500 ms for each image), with inter-stimulus intervals (ISIs) randomized between 500 and 4,500 ms to prevent anticipation effects on perception. The 3,500 ms image presentation time, as empirically justified, allows sufficient duration for participants to both perceive the image and perform semantic classification or quality-assessment tasks.^85^ Although the intervals are randomized, the total duration of each block is controlled to remain exactly 96 s.

The systematic counterbalancing of task block order across runs is implemented to ensure that the tasks are presented in a balanced manner across runs, effectively controlling for task order confounds. Specifically, in odd-numbered runs, the task block sequence followed a QA-CC-CC-QA pattern, while in even-numbered runs, the order is reversed to CC-QA-QA-CC. This systematic counterbalancing allows for a robust counterbalancing between the tasks, ensuring that the influence of task order is minimized.

The order of three quality levels and three content categories is also counterbalanced across runs within participants. The stimulus sequences are carefully designed to integrate category-level temporal optimization with item-level repetition control, ensuring both experimental rigor and statistical efficiency. For each task, optimized randomized sequences of nine stimulus categories are generated using FreeSurfer’s optseq2 tool. ISIs are jittered by randomly selecting durations from 0.5, 2.5, and 4.5 s to reduce temporal autocorrelation and improve event-related signal estimation. Within each category, individual stimuli are presented twice. To manage stimulus repetitions, each stimulus is duplicated, and a constrained randomization procedure employing a backtracking shuffle algorithm is applied. This algorithm enforced a minimum lag of one stimulus between repeated presentations, preventing immediate repetitions and mitigating potential habituation or carryover effects. The resulting item-level sequences are then mapped onto the category-level optseq2 sequence, preserving the optimized timing and ISI structure. The full stimulus sequences are segmented according to block lengths and allocated accordingly to runs and blocks, thereby achieving systematic counterbalancing of task order while maintaining a fixed stimulus presentation sequence across participants.

A fixation cross is presented for 12 s at the beginning and 30 s at the end of each run. Scan data from the last 30 s of each run are treated as the resting state for subsequent analyses. All participants complete eight runs. More information about the fMRI experiment including the instruction for participants is provided in the section “supplemental fMRI experimental setup” in supplemental methods.

### fMRI data acquisition

Visual stimulation is presented using the SINORAD SA-9939 Brain Functional Audiovisual Stimulation system, integrated with E-Prime, with a 40-inch LCD screen for visual stimulation. E-Prime initiated the stimulus task and waited for a synchronous trigger—generated by the MRI device at fMRI sequence onset, transmitted to the main control box—to execute the program, ensuring precise alignment with the brain’s visual and semantic processing timing. The distance between the LED screen and the participants’ eyes is 160 cm, in accordance with the International Telecommunication Union Recommendation ITU-R BT.500-14.^86^ The ambient lighting conditions are maintained constant throughout the experiment. The participants view the display through an angled mirror (45°) attached to the head coil. The stimulation system is synchronized with the MRI system to provide a time reference for the stimulation task and MRI imaging. The stimulus program is written using E-Prime 3.

All MRI data are obtained on a 3T Siemens Prisma scanner equipped with a 32-channel head coil at the Department of Radiology, Renji Hospital School of Medicine, Shanghai Jiao Tong University, Shanghai, China. A gradient echo-planar imaging sequence is employed with the following parameters: repetition time (TR) = 2,000 ms, echo time (TE) = 30 ms, flip angle = 90°, matrix size = 64 × 64, field of view = 192 mm, slice thickness = 2 mm, no inter-slice gap, and 70 axial slices covering the whole brain. The phase-encoding direction used is anterior-posterior. In addition, T1-weighted (T1w) 3D structural images are acquired by using an MPRAGE sequence (TR = 1,800 ms, TE = 2.28 ms, flip angle = 8°, voxel size = 1×1×1 mm), and T2-weighted 3D structural images are acquired by using a turbo spin echo sequence (TR = 9,560 ms, TE = 90 ms, flip angle = 150°, voxel size = 2×2×2 mm).

### fMRI data pre-processing

All MRI data are converted into brain imaging data structure using dcm2niix (v.1.0.20220720). Results included in this article come from pre-processing performed using fMRIPrep 23.1.0.^87^ which is based on Nipype 1.8.6.^88^

Each T1w image is corrected for intensity non-uniformity, skull stripped. Brain tissue segmentation of cerebrospinal fluid (CSF), white matter, and gray matter is performed on the brain-extracted T1w image. Brain surfaces are reconstructed using recon-all.^89^ Volume-based spatial normalization to one standard space (MNI152NLin2009cAsym) is performed, using brain-extracted versions of both T1w reference and the T1w template. Functional images are slice-time corrected, motion corrected, co-registered to the structural image, and normalized to MNI space. See the section “supplemental fMRI data pre-processing” of the supplemental methods for details of anatomical and functional data processing.

After pre-processing by fMRIPrep, the functional data are spatially smoothed with an isotropic 4-mm full-width-half-maximum (FWHM) Gaussian kernel only for univariate analysis and functional connectivity analysis. All analyses in the sections “representation similarity analysis,” “building the response pattern prediction model between ROIs,” and “predicting visual quality using response patterns of ROI pairs” are based on non-smoothed data.

Both smoothed and non-smoothed functional data are denoised using a standard pipeline^90^ that includes regression of potential confounding effects characterized by white matter time series (five CompCor noise components), CSF time series (five CompCor noise components), motion parameters and their first-order derivatives (12 factors),^91^ outlier scans (up to 89 factors),^92^ and linear trends (two factors) within each functional run, followed by band-pass frequency filtering of the BOLD time series^93^ between 0.008 Hz and 0.09 Hz. CompCor^94^^,^^95^ noise components within white matter and CSF are estimated by computing the average BOLD signal and the largest principal components orthogonal to the BOLD average, motion parameters, and outlier scans within each participant’s eroded segmentation masks. Motion parameters, first-order derivatives (12 factors), and outlier scans are confounds extracted in the fMRIPrep analysis stream. Based on the number of noise terms included in this denoising strategy, the effective degrees of freedom of the BOLD signal after denoising are estimated to range from 370.6 to 477.9 (average 434.1) across all participants.^96^

### Univariate analysis

The classical univariate statistical analysis is performed on SPM12.^97^ In the first-level analysis, we use the quality-assessment task and content-classification task as regressors of interest to explore brain mechanisms for quality-assessment tasks and use high quality and low quality as regressors of interest to find brain regions sensitive to image quality. A high-pass filter of 128 s and an AR(1) model are used for signal drift correction and serial correlations, respectively. The statistically significant thresholds for reported group-level analysis results are set at a voxel-level p<0.001 and FDR-corrected *p* < 0.05. The contrast matrix can be found in the section “supplemental univariate analysis” in supplemental notes.

### Seed-based functional connectivity analysis

Results of seed-based functional connectivity come from analyses performed using CONN^98^ and SPM.^97^ After pre-processing and denoising, psychophysiological interaction analyses are used to study the changes in functional connectivity across good, bad, and rest conditions. Seed regions include two automated anatomical labeling (AAL) ROIs.^99^ Separately for each pair of seed and target areas, a generalized psychophysiological interaction model (gPPI^100^^,^^101^) is defined with seed BOLD signals as physiological factors, boxcar signals characterizing each task condition convolved with an SPM canonical hemodynamic response function as psychological factors, and the product of the two as psychophysiological interaction terms. Functional connectivity changes across conditions are characterized by the multivariate regression coefficient of the psychophysiological interaction terms in each model.

Group-level analyses are performed using a GLM.^90^ For each voxel, a separate GLM is estimated, with first-level connectivity measures at this voxel as dependent variables (one independent sample per participant and one measurement per task or experimental condition, if applicable) and groups or other participant-level identifiers as independent variables. Voxel-level hypotheses are evaluated using multivariate parametric statistics with random effects across participants and sample covariance estimation across multiple measurements. Inferences are performed at the level of individual clusters (groups of contiguous voxels). Cluster-level inferences are based on parametric statistics from Gaussian random field theory.^90^^,^^102^ Results are thresholded using a combination of a cluster-forming p<0.01 voxel-level threshold, and a family-wise corrected p−FDR<0.05 cluster-size threshold.

### Single trial betas and patterns of each ROI

To extract single-trial fMRI response patterns for each ROI in a given run, we first convolve the onset times of each trial with the hemodynamic response function corresponding to each trial; we then conduct a GLM analysis to extract the beta weights for each voxel within that ROI for each trial. It is important to emphasize that the fMRI data used here are pre-processed and denoised using fMRIPrep but are not spatially smoothed. These voxel beta weights are used as the fMRI response patterns for that trial during the run.

We extract visually evoked responses from seven vision-related ROIs, including the calcarine, cuneus, lingual, superior occipital gyrus, middle occipital gyrus, inferior occipital gyrus, and fusiform gyrus. The boundaries of these ROIs are determined based on the AAL template,^99^ which provides a consistent and reproducible method of quantifying brain activity and structure across different individuals and studies.

To reduce the dimensionality of response patterns within each ROI and enhance their reliability, facilitating the establishment of mappings between ROIs, we select the 100 voxels with the highest reliability from each unilateral ROI. To calculate the reliability of each voxel within each ROI, we use a GLM to compute the beta weights corresponding to 18 conditions (derived from two task conditions, three image content categories, and three quality levels, thus 2×3×3) for each run. The data are split into odd and even halves by run, and we average the data across runs within each half. We correlate the beta weights from all conditions between the two halves for each voxel. Subsequently, the top 100 voxels exhibiting the highest correlations are selected from each unilateral ROI. Masks composed of these voxels are then applied to the beta weights corresponding to each image to derive the response patterns for the respective ROIs. For more detailed information, see Tarhan and Konkle.^103^ Additionally, the methodology has been employed in works such as those by Xu and Vaziri-Pashkam^27^ and Cadieu et al.,^104^ where Xu and Vaziri-Pashkam^27^ specifically selected 75 voxels demonstrating the highest correlation in each ROI.

We use the beta weights of these selected voxels to characterize the response pattern of the unilateral ROI to each image. The response patterns from the left and right sides of the same ROI are then concatenated to represent the response of that ROI to each image. We apply *Z*-score normalization to the averaged pattern for each condition in each ROI to remove amplitude differences between conditions and ROIs.

### Representation similarity analysis

To investigate how visual quality impacts response patterns within the visual pathway, we employ RSA^44^ to quantify the semantic information content across various ROIs with RSAtoolbox.^105^ Initially, semantic labels for faces, objects, and scenes are encoded as three one-hot vectors, i.e., (1, 0, 0), (0, 1, 0), and (0, 0, 1), with each image uniquely associated with one semantic label. The quality labels for bad, neutral, and excellent are encoded as (0, 1), (1, 1), and (1, 0), respectively, with each image uniquely associated with a quality label. Subsequently, we calculate the correlation dissimilarity between the ideal semantic labels of the images as the ideal semantic RDM and the Euclidean dissimilarity between the ideal quality labels as the ideal quality RDM. For each participant, the representations extracted as described above corresponding to the repeated presentations of the same image are averaged to serve as the response estimate for that image.

Similarly, we compute the dissimilarity between the response patterns of all image pairs within each ROI, independently forming each ROI’s RDM for each participant. The dissimilarity between response patterns is measured using the Pearson correlation coefficient, which is as reliable as Euclidean-like dissimilarities.^106^ The correlation distance depends not only on the differences between two patterns but also on shared overall activations, such that additional shared activity, which does not impair decoding, still reduces the correlation distance.

Subsequently, the similarity between the ideal semantic RDM and the RDM of each ROI for every participant is calculated, serving as a representation of the semantic information content within each ROI for each participant. Results on similarity obtained using the Spearman correlation coefficient are presented in Figure S5 and lead to conclusions consistent with those derived from the Pearson correlation. Similarly, the similarity between the ideal quality RDM and the RDM of each ROI for every participant is calculated, serving as a representation of the quality information content within each ROI for each participant.

In this study, we employ Welch’s ANOVA and the Games-Howell post hoc test to evaluate the effects of three levels of image quality on the semantic content associated with ROI. The use of Welch’s ANOVA is necessitated by the lack of homogeneity of variances among the groups. Welch’s ANOVA is utilized to detect any statistically significant differences across the image-quality groups, and, where significant differences are found, the Games-Howell test is applied for post hoc pairwise comparisons to identify specific group differences.

### Modeling the mapping relationship between response patterns of any two brain regions

This study utilizes an SVR model with a radial basis function (RBF) kernel to build the mapping between ROI response patterns. The model is configured with a regularization parameter (*C*) of 1 and an epsilon of 0.1, balancing the trade-off between fitting the training data and preventing overfitting while enhancing robustness to outliers. This configuration is chosen for its effectiveness in capturing non-linear relationships within the data. The models are independently trained for each dimension of the representational vectors, ensuring a robust method for modeling the complex mappings between the ROI response patterns. The SVR model’s input is the representational vector of ROI *A*, and the output is the representational vector of ROI *B*, both consistent with the representational vectors extracted in the section “single trial betas and patterns of each ROI,” each with a length of 200.

To mitigate the impact of shared signals between brain regions, we employ cross-prediction using data from two repeated measurements. Specifically, the response pattern of ROI *A* from the first measurement is used to predict the response pattern of ROI *B* from the second measurement and vice versa.

The linear correlation between the actual and predicted representational vectors of ROI *B* is calculated to assess the model’s predictive performance. Let y be the actual representational vector of ROI *B* and $\hat{y}$ be the predicted representational vector of ROI *B*. The linear correlation ρ can be expressed as

$$\rho =\frac{\text{Cov}\left(y,\hat{y}\right)}{\sqrt{\text{Var}\left(y\right)\cdot \text{Var}\left(\hat{y}\right)}}\text{,}$$

where $\text{Cov}\left(y,\hat{y}\right)$ is the covariance between y and $\hat{y},\text{and}\;\text{Var}\left(y\right)$ and $\text{Var}\left(\hat{y}\right)$ are the variances of y and $\hat{y}$, respectively.

In this study, each mapping relationship for each participant is trained independently. For each participant, the data under each quality condition are randomly divided into four folds and subjected to cross-validation. The reported performance is the average result from multiple rounds of cross-validation, where the data for each participant are randomly divided into four folds ten times, with each split being independently trained. It is important to note that we trained the model using data from only one quality condition (either high or low quality) and then tested it across all three quality conditions (high, neutral, and low). The test set for each of the three quality conditions corresponds to the same fold number. For example, if the model is trained using the first, third, and fourth folds of the high-quality data, the second fold is used for testing across all three conditions.

To examine whether visual quality significantly affects the distribution of model performance among participants, we conduct a Welch’s ANOVA analysis followed by Games-Howell post hoc tests. Mappings with *p* values less than 0.05 are marked in the figures.

To explore the theoretical performance ceiling of the predictive model, reflecting the inherent variability of the data itself, we calculate the noise ceiling for each participant and each ROI. The noise ceiling for a given participant p and ROI r is computed by evaluating the linear correlation between the representations of repeated measurements for each image. For each image i, we compute the linear correlation $\rho_{p,r,i}$ between the representations of repeated measurements. This can be expressed as

$$\rho_{p,r,i}=\frac{\text{Cov}\left(X_{p,r,i},X_{p,r,i}^{\text{repeat}}\right)}{\sqrt{\text{Var}\left(X_{p,r,i}\right)\cdot \text{Var}\left(X_{p,r,i}^{\text{repeat}}\right)}}\text{,}$$

where $X_{p,r,i}$ is the representation of the image i for participant p in ROI $r,\text{and}\;X_{p,r,i}^{\text{repeat}}$ is the repeated measurement representation for the same image. Next, the average correlation across all images for participant p and ROI r is calculated:

$${\bar{\rho }}_{p,r}=\frac{1}{N}\sum_{i=1}^{N}\rho_{p,r,i}\text{,}$$

where N is the total number of images.

In Figures 4, S6, and S7, the average noise ceiling represents the average of these correlations across all participants for each ROI is then taken as the noise ceiling for that ROI:

$${\bar{\rho }}_{r}=\frac{1}{M}\sum_{p=1}^{M}{\bar{\rho }}_{p,r}\text{,}$$

where M is the total number of participants.

### Predicting visual quality using response patterns of any two brain regions

To validate the feasibility of decoding visual quality information from fMRI data, considering the available data scale, SVR models with RBF kernels are chosen to regress visual quality scores from the representational vectors of various ROIs. The input to the predictive model for a single ROI consists of the representational vector for that ROI, while for combined ROIs the input vector is formed by concatenating the representational vectors of ROI *A* and ROI *B*. All representational vectors are consistent with those used in the previous RSA. The SVR models are configured with a regularization parameter (*C*) of 1 and an epsilon of 0.1. The actual quality scores of the images are quantized into discrete integers 3 for high quality, 2 for neutral quality, and 1 for low quality, while the output of the predictive model is a continuous floating-point number.

The performance of the predictive model is characterized using the SRCC and PLCC. The SRCC measures the strength and direction of the monotonic relationship between the predicted and actual quality scores and is defined mathematically as

$$\text{SRCC}=1-\frac{6\sum d_{i}^{2}}{n\left(n^{2}-1\right)}\text{,}$$

where $d_{i}$ is the difference between the ranks of the ith observation in the predicted and actual scores, and n is the number of observations. The PLCC, on the other hand, assesses the linear correlation between the predicted and actual quality scores, given by

$$\text{PLCC}=\frac{\sum \left(x_{i}-\bar{x}\right)\left(y_{i}-\bar{y}\right)}{\sqrt{\sum {\left(x_{i}-\bar{x}\right)}^{2}\sum {\left(y_{i}-\bar{y}\right)}^{2}}}\text{,}$$

where $x_{i}$ and $y_{i}$ are the predicted and actual scores, respectively, and $\bar{x}$ and $\bar{y}$ are their respective means. Both SRCC and PLCC are essential for evaluating the accuracy and reliability of quality decoding models, with SRCC focusing on rank correlation and PLCC on linear correlation.

The predictive models for each combination of ROIs for each participant are trained independently, with all models undergoing ten rounds of four-fold cross-validation. The predictive performance for each combination of ROIs for each participant is calculated as the average result across these multiple rounds of cross-validation.

### Implementation detail of brain-inspired multi-layer feature fusion strategy

Based on the conclusions from the fMRI experiments, we propose a multi-layer feature fusion strategy to enhance the visual quality prediction capability of CNNs by combining features from different stages. The effectiveness of this strategy is validated on various CNN backbones on various IQA datasets (CLIVE, KonIQ-10k, and BID).

We choose SqueezeNet, ResNet, and EfficientNet as backbones to evaluate the adaptability of our feature fusion method across architectures with different complexities. SqueezeNet offers lightweight efficiency, ResNet introduces effective residual learning, and EfficientNet balances accuracy and computational cost through compound scaling. This selection enables a comprehensive assessment of our method’s generalizability. The detailed architecture of the backbone networks used in this study is as follows.

(1) ResNet-50^54^: ResNet introduces residual learning via identity shortcut connections, which enable the network to learn residual functions relative to the input. The architecture of ResNet-50 consists of 50 layers, organized into four stages, each containing a series of residual blocks. The first stage includes three residual blocks, the second stage has four, the third stage contains six, and the fourth stage comprises three. Each residual block employs skip connections that bypass one or more layers and are added to the output, facilitating the flow of gradients during backpropagation and enhancing the learning process.
(2) SqueezeNet^53^: SqueezeNet is a lightweight convolutional neural network designed to achieve AlexNet-level accuracy with significantly fewer parameters and reduced model size. Its architecture is built around “fire modules,” which consist of a squeeze convolution layer using 1 × 1 filters followed by an expand layer that combines 1 × 1 and 3 × 3 convolutions. This design efficiently reduces the number of parameters while maintaining representational capacity, making SqueezeNet suitable for resource-constrained environments without compromising performance.
(3) EfficientNet-V2-M^55^: EfficientNet-V2-M is a convolutional neural network architecture that improves upon the original EfficientNet by optimizing both model accuracy and training speed. It employs a compound scaling method that uniformly scales network depth, width, and resolution, combined with progressive learning techniques. The architecture consists of mobile inverted bottleneck convolution (MBConv) and fused-MBConv blocks, enabling efficient feature extraction with reduced computational cost. EfficientNet-V2-M achieves a strong balance between accuracy and efficiency, making it suitable for a wide range of vision tasks.

By selecting these three architectures, we cover a broad spectrum of model complexity—from lightweight to deep and efficiently scaled networks. This diversity allows us to comprehensively evaluate the generalizability and robustness of our proposed feature fusion strategy across different backbone designs and computational budgets, thereby strengthening the validity of our approach.

The CLIVE, KonIQ-10k, and BID datasets are selected to validate the effectiveness of the multi-layer feature fusion strategy. These three IQA datasets consist of images with authentic natural distortions encompassing diverse distortion types and varying scales, making them well suited for comprehensive evaluation of the proposed strategy’s effectiveness and generalizability. The details of the datasets used in this study are as follows.

(1) CLIVE^57^: the LIVE In the Wild Image Quality Challenge database is a comprehensive IQA dataset comprising 1,162 authentically distorted images captured from various mobile devices. Unlike traditional datasets with synthetic distortions, it features real-world distortions such as compression artifacts, noise, blur, and color distortions. The dataset’s subjective quality scores are collected via a large-scale crowdsourcing study involving over 8,100 unique observers, with each image rated by approximately 175 participants, yielding over 350,000 opinion scores.
(2) KonIQ-10k^42^: KonIQ-10k is a large-scale no-reference IQA dataset designed to support the training and evaluation of deep-learning models. It comprises 10,073 images, each annotated with subjective MOS collected from approximately 120 independent raters per image, totaling over 1.2 million ratings. The images exhibit a wide variety of authentic distortions common in real-world scenarios, including compression artifacts, noise, and blur, reflecting diverse everyday capture conditions. Sourced from the YFCC100M dataset, KonIQ-10k ensures content diversity and naturalness.
(3) BID^58^: the BID (Blurred Image Database) is a specialized no-reference IQA dataset focusing on blur distortions. It contains 586 images exhibiting various types of blur, including out-of-focus, simple-motion, and complex-motion blur, captured with consumer-grade digital cameras to reflect realistic photographic conditions. Each image has been rated by multiple human observers to obtain subjective MOS, which serve as ground truth for training and evaluating no-reference IQA models. This dataset is particularly valuable for developing algorithms aimed at assessing image quality where blur is the primary degradation.

We primarily use pooling or fully connected (FC) layers as markers to divide the network into five stages. Pooling layers are selected because they typically signify the end of a block, merging information and passing it to the next block.^27^^,^^107^ In the absence of pooling or FC layers, the last layer of a block is chosen as the marker for stage division. Features from each stage during the information propagation process are extracted, adjusted to a uniform dimension through convolution layers, and fused to form multi-layer features, which replace the final layer’s features of the backbone. The features involved in multi-layer feature fusion across each path in each network are shown in Figure S12. These fused features are passed into a regressor to produce the final quality prediction score. The regressor of each backbone is designed to closely match the regressor structure used with its corresponding backbone in the ImageNet classification task, ensuring the final output dimension is 1.

All backbones are pre-trained on ImageNet.^56^ All networks are independently fine-tuned and tested on the aforementioned IQA datasets using five-fold cross-validation. The dataset split and all training parameters remain consistent across backbones and datasets. Each network is trained for 30 epochs using a batch size of 64 on each dataset. Input images are first resized to 384 pixels and then center-cropped to 320 pixels, maintaining the aspect ratio. The initial learning rate is set to 0.0001, which decays by a factor of 0.5 every 5 epochs to facilitate stable convergence. All experiments are implemented using the PyTorch framework on a server equipped with an NVIDIA RTX 4090.

## Resource availability

### Lead contact

Requests for further information and resources should be directed to and will be fulfilled by the lead contact, Guangtao Zhai (zhaiguangtao@sjtu.edu.cn).

### Materials availability

The authors declare that the raw fMRI data in BIDS format will be made available on https://openneuro.org/datasets/ds006483.

### Data and code availability

• The authors declare that the main data supporting the findings of this study, as well as the raw fMRI data in BIDS format, are available on https://openneuro.org/datasets/ds006483.^108^
• The codes that support the findings of this study are available at figshare.^109^

## Acknowledgments

This work is supported in part by the National Natural Science Foundation of China (no. 62225112), National Natural Science Foundation of China (no. 62271312), National Key R&D Program of China (no. 2024YFB3614600), National Natural Science Foundation of China (nos. 82171885 and 82302142), Shanghai Science and Technology Committee Project (Explorer Project Funding: grant no. 24TS1414800), the Leading Talent of Shanghai Municipal Health Commission no. 2022LJ023, and Eastern Talent Plan Leading Project (LJ2023127). The funders had no role in study design, data collection and analysis, decision to publish, or preparation of the paper.

## Author contributions

G.Z., X.M., Y. Zhou, Y.C., Y. Zhang, Y.H., X.H., Z.X., and X.W. contributed to the conceptualization and design of the experiment. G.Z., X.M., and Y. Zhou supervised the study and acquired funding. Y. Zhang and Y.H. created the fMRI task and stimuli, programmed the fMRI experiment, and analyzed (including writing code) the data. Y. Zhou, Y.H., X.H., Z.X., and X.W. contributed to the fMRI data collection, interpretation, and process supervision. Y. Zhang, Y.C., and Y.H. wrote the manuscript.

## Declaration of interests

The authors declare no competing interests.

## Declaration of generative AI and AI-assisted technologies in the writing process

During the preparation of this work, the authors used ChatGPT-4o in order to improve the readability and language of the manuscript. After using this tool/service, the authors reviewed and edited the content as needed and take full responsibility for the content of the published article.

## Supplemental information

Document S1. Figures S1–S14, Tables S1–S5, supplemental methods, and supplemental notes

Table S6. The SRCC of visual quality predicting models

Table S7. The PLCC of visual quality predicting models

Document S2. Article plus supplemental information

## References

[1] Ericsson (2024). Mobile traffic update. https://www.ericsson.com/en/reports-and-papers/mobility-report/dataforecasts/mobile-traffic-update.

[2] Shahid M., Rossholm A., Lövström B., Zepernick H.J. (2014). No-reference image and video quality assessment: a classification and review of recent approaches. EURASIP J. Image Video Process..

[3] Zhai G., Min X. (2020). Perceptual image quality assessment: a survey. Sci. China Inf. Sci..

[4] Chen Y., Wu K., Zhang Q. (2015). From QOS to QOE: A tutorial on video quality assessment. IEEE Commun. Surv. Tutorials.

[5] Wang Z., Bovik A.C., Sheikh H.R., Simoncelli E.P. (2004). Image quality assessment: from error visibility to structural similarity. IEEE Trans. Image Process..

[6] Sheikh H.R., Bovik A.C., Cormack L. (2005). No-reference quality assessment using natural scene statistics: Jpeg2000. IEEE Trans. Image Process..

[7] Mittal A., Soundararajan R., Bovik A.C. (2013). Making a “completely blind” image quality analyzer. IEEE Signal Process. Lett..

[8] Zhai G., Wu X., Yang X., Lin W., Zhang W. (2012). A psychovisual quality metric in free-energy principle. IEEE Trans. Image Process..

[9] Mantiuk R. (2007). High dynamic range imaging: towards the limits of the human visual perception. Forsch. Wiss. Rechnen.

[10] Mozhaeva A., Vlasuyk I., Potashnikov A., Mazin V., Streeter L. (2024). 2024 39th International Conference on Image and Vision Computing New Zealand (IVCNZ).

[11] Gu K., Zhai G., Yang X., Zhang W. (2015). Using free energy principle for blind image quality assessment. IEEE Trans. Multimedia.

[12] Mozhaeva A., Streeter L., Vlasuyk I., Potashnikov A. (2021). 2021 28th Conference of Open Innovations Association (FRUCT).

[13] Mazin V., Cree M.J., Streeter L., Nezhivleva K., Mozhaeva A. (2023). 2023 33rd Conference of Open Innovations Association (FRUCT).

[14] Mantiuk R., Krawczyk G., Myszkowski K., Seidel H.P. (2004). Perception-motivated high dynamic range video encoding. ACM Trans. Graph..

[15] Zhang W., Ma K., Yan J., Deng D., Wang Z. (2020). Blind image quality assessment using a deep bilinear convolutional neural network. IEEE Trans. Circuits Syst. Video Technol..

[16] Sun W., Duan H., Min X., Chen L., Zhai G. (2022). IEEE International Symposium on Broadband Multimedia Systems and Broadcasting (BMSB).

[17] Zhu H., Li L., Wu J., Dong W., Shi G. (2020). IEEE/CVF Conference on Computer Vision and Pattern Recognition (CVPR).

[18] Wu H., Zhang Z., Zhang E., Chen C., Liao L., Wang A., Xu K., Li C., Hou J., Zhai G. (2024). IEEE/CVF Conference on Computer Vision and Pattern Recognition (CVPR).

[19] Gauthier I., Tarr M.J., Anderson A.W., Skudlarski P., Gore J.C. (1999). Activation of the middle fusiform ‘face area’ increases with expertise in recognizing novel objects. Nat. Neurosci..

[20] Cichy R.M., Pantazis D., Oliva A. (2014). Resolving human object recognition in space and time. Nat. Neurosci..

[21] Yamins D.L.K., Hong H., Cadieu C.F., Solomon E.A., Seibert D., DiCarlo J.J. (2014). Performance-optimized hierarchical models predict neural responses in higher visual cortex. Proc. Natl. Acad. Sci. USA.

[22] Grill-Spector K., Weiner K.S. (2014). The functional architecture of the ventral temporal cortex and its role in categorization. Nat. Rev. Neurosci..

[23] Huth A.G., De Heer W.A., Griffiths T.L., Theunissen F.E., Gallant J.L. (2016). Natural speech reveals the semantic maps that tile human cerebral cortex. Nature.

[24] Huth A.G., Nishimoto S., Vu A.T., Gallant J.L. (2012). A continuous semantic space describes the representation of thousands of object and action categories across the human brain. Neuron.

[25] Cichy R.M., Khosla A., Pantazis D., Torralba A., Oliva A. (2016). Comparison of deep neural networks to spatio-temporal cortical dynamics of human visual object recognition reveals hierarchical correspondence. Sci. Rep..

[26] Eickenberg M., Gramfort A., Varoquaux G., Thirion B. (2017). Seeing it all: Convolutional network layers map the function of the human visual system. Neuroimage.

[27] Xu Y., Vaziri-Pashkam M. (2021). Limits to visual representational correspondence between convolutional neural networks and the human brain. Nat. Commun..

[28] Zhang Z., Wu W., Sun W., Tu D., Lu W., Min X., Chen Y., Zhai G. (2023). IEEE/CVF Conference on Computer Vision and Pattern Recognition (CVPR).

[29] Sun W., Min X., Lu W., Zhai G. (2022). ACM International Conference on Multimedia.

[30] Yang L., Duan H., Teng L., Zhu Y., Liu X., Hu M., Min X., Zhai G., Le Callet P. (2024). IEEE International Conference on Image Processing (ICIP).

[31] Sun W., Min X., Zhai G., Gu K., Duan H., Ma S. (2020). Mc360iqa: A multi-channel CNN for blind 360-degree image quality assessment. IEEE J. Sel. Top. Signal Process..

[32] Duan H., Zhai G., Min X., Zhu Y., Fang Y., Yang X. (2018). IEEE International Symposium on Circuits and Systems (ISCAS).

[33] Li C., Zhang Z., Wu H., Sun W., Min X., Liu X., Zhai G., Lin W. (2024). Agiqa-3k: An open database for AI-generated image quality assessment. IEEE Trans. Circuits Syst. Video Technol..

[34] Liu X., Min X., Zhai G., Li C., Kou T., Sun W., Wu H., Gao Y., Cao Y., Zhang Z. (2024). IEEE/CVF Conference on Computer Vision and Pattern Recognition (CVPR).

[35] Zhang Z., Li C., Sun W., Liu X., Min X., Zhai G. (2023). IEEE International Conference on Multimedia and Expo Workshops (ICMEW).

[36] Qiu J., Zhu Y., Shi X., Wenzel F., Tang Z., Zhao D., Li B., Li M. (2023). Benchmarking robustness of multimodal image-text models under distribution shift. Arxiv.

[37] Bosse S., Brunnström K., Arndt S., Martini M.G., Ramzan N., Engelke U. (2019). A common framework for the evaluation of psychophysiological visual quality assessment. Qual. User Exp.

[38] Arndt S., Antons J.N., Schleicher R., Möller S., Curio G. (2014). Using electroencephalography to measure perceived video quality. IEEE J. Sel. Top. Signal Process..

[39] Arndt S., Antons J.N., Schleicher R., Moller S., Scholler S., Curio G. (2011). IEEE International Symposium on Multimedia.

[40] Liu X., Tao X., Xu M., Zhan Y., Lu J. (2020). An EEG-based study on perception of video distortion under various content motion conditions. IEEE Trans. Multimedia.

[41] Hu S., Duan Y., Tao X., Li G.Y., Lu J., Liu G., Zheng Z., Pan C. (2024). Brain-inspired image perceptual quality assessment based on EEG: A QOE perspective. IEEE Trans. Pattern Anal. Mach. Intell..

[42] Hosu V., Lin H., Sziranyi T., Saupe D. (2020). Koniq-10k: An ecologically valid database for deep learning of blind image quality assessment. IEEE Trans. Image Process..

[43] Ogawa S., Lee T.M., Kay A.R., Tank D.W. (1990). Brain magnetic resonance imaging with contrast dependent on blood oxygenation. Proc. Natl. Acad. Sci. USA.

[44] Kriegeskorte N., Mur M., Bandettini P. (2008). Representational similarity analysis-connecting the branches of systems neuroscience. Front. Syst. Neurosci..

[45] Kriegeskorte N., Formisano E., Sorger B., Goebel R. (2007). Individual faces elicit distinct response patterns in human anterior temporal cortex. Proc. Natl. Acad. Sci. USA.

[46] Nestor A., Plaut D.C., Behrmann M. (2011). Unraveling the distributed neural code of facial identity through spatiotemporal pattern analysis. Proc. Natl. Acad. Sci. USA.

[47] Friston K., Kilner J., Harrison L. (2006). A free energy principle for the brain. J. Physiol. Paris.

[48] Friston K. (2010). The free-energy principle: a unified brain theory?. Nat. Rev. Neurosci..

[49] Liu Z., Lin Y., Cao Y., Hu H., Wei Y., Zhang Z., Lin S., Guo B. (2021). Proceedings of the IEEE/CVF International Conference on Computer Vision (ICCV).

[50] Gong Z., Bao G., Zhang Q., Wan Z., Miao D., Wang S., Zhu L., Wang C., Xu R., Hu L. (2024). Neuroclips: Towards high-fidelity and smooth fMRI-to-video reconstruction. Adv. Neural Inf. Process. Syst..

[51] Güçlü U., van Gerven M.A.J. (2017). Increasingly complex representations of natural movies across the dorsal stream are shared between subjects. Neuroimage.

[52] Khaligh-Razavi S.M., Kriegeskorte N. (2014). Deep supervised, but not unsupervised, models may explain it cortical representation. PLoS Comput. Biol..

[53] Iandola F.N., Han S., Moskewicz M.W., Ashraf K., Dally W.J., Keutzer K. (2016). Squeezenet: Alexnet-level accuracy with 50x fewer parameters and <0.5 mb model size. ArXiv.

[54] He K., Zhang X., Ren S., Sun J. (2016). IEEE/CVF Conference on Computer Vision and Pattern Recognition (CVPR).

[55] Tan M., Le Q. (2021). International Conference on Machine Learning (ICML).

[56] Deng J., Dong W., Socher R., Li L.J., Li K., Fei-Fei L. (2009). IEEE/CVF Conference on Computer Vision and Pattern Recognition (CVPR).

[57] Ghadiyaram D., Bovik A.C. (2016). Massive online crowdsourced study of subjective and objective picture quality. IEEE Trans. Image Process..

[58] Ciancio A., Targino da Costa A.L.N., da Silva E.A., Said A., Samadani R., Obrador P. (2010). No-reference blur assessment of digital pictures based on multifeature classifiers. IEEE Trans. Image Process..

[59] Chen F., Fu H., Yu H., Chu Y. (2023). Using HVS dual-pathway and contrast sensitivity to blindly assess image quality. Sensors.

[60] Kang L., Ye P., Li Y., Doermann D. (2014). IEEE/CVF Conference on Computer Vision and Pattern Recognition (CVPR).

[61] Bosse S., Maniry D., Müller K.R., Wiegand T., Samek W. (2017). Deep neural networks for no-reference and full-reference image quality assessment. IEEE Trans. Image Process..

[62] Li D., Jiang T., Lin W., Jiang M. (2019). Which has better visual quality: The clear blue sky or a blurry animal?. IEEE Trans. Multimedia.

[63] Su S., Yan Q., Zhu Y., Zhang C., Ge X., Sun J., Zhang Y. (2020). IEEE/CVF Conference on Computer Vision and Pattern Recognition (CVPR).

[64] Zhang Z., Wu H., Zhang E., Zhai G., Lin W. (2024). Q-bench ++: A benchmark for multi-modal foundation models on low-level vision from single images to pairs. IEEE Trans. Pattern Anal. Mach. Intell..

[65] Zhang L., Zhang L., Mou X., Zhang D. (2011). Fsim: A feature similarity index for image quality assessment. IEEE Trans. Image Process..

[66] Tops M., Boksem M.A.S. (2011). A potential role of the inferior frontal gyrus and anterior insula in cognitive control, brain rhythms, and event-related potentials. Front. Psychol..

[67] Hartwigsen G., Neef N.E., Camilleri J.A., Margulies D.S., Eickhoff S.B. (2019). Functional segregation of the right inferior frontal gyrus: evidence from coactivation-based parcellation. Cereb. Cortex.

[68] Hong H., Yamins D.L.K., Majaj N.J., DiCarlo J.J. (2016). Explicit information for category-orthogonal object properties increases along the ventral stream. Nat. Neurosci..

[69] DiCarlo J.J., Zoccolan D., Rust N.C. (2012). How does the brain solve visual object recognition?. Neuron.

[70] Felleman D.J., Van Essen D.C. (1991). Distributed hierarchical processing in the primate cerebral cortex. Cereb. Cortex.

[71] Greenberg D.L., Rice H.J., Cooper J.J., Cabeza R., Rubin D.C., LaBar K.S. (2005). Co-activation of the amygdala, hippocampus and inferior frontal gyrus during autobiographical memory retrieval. Neuropsychologia.

[72] Fletcher P.C., Henson R.N. (2001). Frontal lobes and human memory: insights from functional neuroimaging. Brain.

[73] Wagner A.D., Paré-Blagoev E.J., Clark J., Poldrack R.A. (2001). Recovering meaning: left prefrontal cortex guides controlled semantic retrieval. Neuron.

[74] Rahate A., Mandaokar S., Chandel P., Walambe R., Ramanna S., Kotecha K. (2023). Employing multimodal co-learning to evaluate the robustness of sensor fusion for industry 5.0 tasks. Soft Comput..

[75] Guo R., Wei J., Sun L., Yu B., Chang G., Liu D., Zhang S., Yao Z., Xu M., Bu L. (2023). A survey on image-text multimodal models. arXiv.

[76] Long Z., Killick G., McCreadie R., Camarasa G.A. (2024). IEEE International Conference on Acoustics, Speech and Signal Processing (ICASSP).

[77] Sweller J. (1988). Cognitive load during problem solving: Effects on learning. Cogn. Sci..

[78] De Jong T. (2010). Cognitive load theory, educational research, and instructional design: Some food for thought. Instr. Sci..

[79] Chen O., Paas F., Sweller J. (2023). A cognitive load theory approach to defining and measuring task complexity through element interactivity. Educ. Psychol. Rev..

[80] Winding M., Pedigo B.D., Barnes C.L., Patsolic H.G., Park Y., Kazimiers T., Fushiki A., Andrade I.V., Khandelwal A., Valdes-Aleman J. (2023). The connectome of an insect brain. Science.

[81] Chang N., Pyles J.A., Marcus A., Gupta A., Tarr M.J., Aminoff E.M. (2019). Bold5000, a public fMRI dataset while viewing 5000 visual images. Sci. Data.

[82] Allen E.J., St-Yves G., Wu Y., Breedlove J.L., Prince J.S., Dowdle L.T., Nau M., Caron B., Pestilli F., Charest I. (2022). A massive 7t fMRI dataset to bridge cognitive neuroscience and artificial intelligence. Nat. Neurosci..

[83] Holladay J.T. (2004). Visual acuity measurements. J. Cataract Refract. Surg..

[84] Thomee B., Shamma D.A., Friedland G., Elizalde B., Ni K., Poland D., Borth D., Li L.J. (2016). Yfcc100m: The new data in multimedia research. Commun. ACM.

[85] Gao Y., Min X., Zhai G. (2025). Exploring image quality assessment from a new perspective: Pupil size. arXiv.

[86] International Telecommunication Union (ITU) (2012). https://www.itu.int/dms_pubrec/itu-r/rec/bt/R-REC-BT.500-14-201206-I!!PDF-E.pdf.

[87] Esteban O., Markiewicz C.J., Blair R.W., Moodie C.A., Isik A.I., Erramuzpe A., Kent J.D., Goncalves M., DuPre E., Snyder M. (2019). fMRIPrep: a robust preprocessing pipeline for functional MRI. Nat. Methods.

[88] Gorgolewski K., Burns C.D., Madison C., Clark D., Halchenko Y.O., Waskom M.L., Ghosh S.S. (2011). Nipype: a flexible, lightweight and extensible neuroimaging data processing framework in python. Front. Neuroinform..

[89] Dale A.M., Fischl B., Sereno M.I. (1999). Cortical surface-based analysis: I. segmentation and surface reconstruction. Neuroimage.

[90] Nieto-Castanon A. (2020).

[91] Friston K.J., Williams S., Howard R., Frackowiak R.S., Turner R. (1996). Movement-related effects in fMRI time-series. Magn. Reson. Med..

[92] Power J.D., Mitra A., Laumann T.O., Snyder A.Z., Schlaggar B.L., Petersen S.E. (2014). Methods to detect, characterize, and remove motion artifact in resting state fMRI. Neuroimage.

[93] Hallquist M.N., Hwang K., Luna B. (2013). The nuisance of nuisance regression: spectral misspecification in a common approach to resting-state fMRI preprocessing reintroduces noise and obscures functional connectivity. Neuroimage.

[94] Behzadi Y., Restom K., Liau J., Liu T.T. (2007). A component based noise correction method (compcor) for bold and perfusion based fmri. Neuroimage.

[95] Chai X.J., Castañón A.N., Ongür D., Whitfield-Gabrieli S. (2012). Anticorrelations in resting state networks without global signal regression. Neuroimage.

[96] Nieto-Castanon A. (2022). Preparing fMRI data for statistical analysis. arXiv.

[97] Penny W.D., Friston K.J., Ashburner J.T., Kiebel S.J., Nichols T.E. (2011).

[98] Whitfield-Gabrieli S., Nieto-Castanon A. (2012). Conn: a functional connectivity toolbox for correlated and anticorrelated brain networks. Brain Connect..

[99] Tzourio-Mazoyer N., Landeau B., Papathanassiou D., Crivello F., Etard O., Delcroix N., Mazoyer B., Joliot M. (2002). Automated anatomical labeling of activations in SPM using a macroscopic anatomical parcellation of the MNI MRI single-subject brain. Neuroimage.

[100] Friston K.J., Buechel C., Fink G.R., Morris J., Rolls E., Dolan R.J. (1997). Psychophysiological and modulatory interactions in neuroimaging. Neuroimage.

[101] McLaren D.G., Ries M.L., Xu G., Johnson S.C. (2012). A generalized form of context-dependent psychophysiological interactions (gPPI): a comparison to standard approaches. Neuroimage.

[102] Worsley K.J., Marrett S., Neelin P., Vandal A.C., Friston K.J., Evans A.C. (1996). A unified statistical approach for determining significant signals in images of cerebral activation. Hum. Brain Mapp..

[103] Tarhan L., Konkle T. (2020). Reliability-based voxel selection. Neuroimage.

[104] Cadieu C.F., Hong H., Yamins D.L.K., Pinto N., Ardila D., Solomon E.A., Majaj N.J., DiCarlo J.J. (2014). Deep neural networks rival the representation of primate it cortex for core visual object recognition. PLoS Comput. Biol..

[105] Nili H., Wingfield C., Walther A., Su L., Marslen-Wilson W., Kriegeskorte N. (2014). A toolbox for representational similarity analysis. PLoS Comput. Biol..

[106] Walther A., Nili H., Ejaz N., Alink A., Kriegeskorte N., Diedrichsen J. (2016). Reliability of dissimilarity measures for multi-voxel pattern analysis. Neuroimage.

[107] O’Connell T.P., Chun M.M. (2018). Predicting eye movement patterns from fmri responses to natural scenes. Nat. Commun..

[108] Yiming, Z., Yitong, C., Ying, H., Xu, H., Zhenhui, X., Xingrui, W., Yan, Z., Xiongkuo, M., and Guangtao, Z. (2025). Neural mechanisms of visual quality perception and adaptability in visual pathway. OpenNeuro [dataset]. doi: 10.18112/openneuro.ds006483.v1.0.0

[109] Yiming, Z., Yitong, C., Ying, H., Xu, H., Zhenhui, X., Xingrui, W., Yan, Z., Xiongkuo, M., and Guangtao, Z. (2025). fMRI for VQA code. figshare. 10.6084/m9.figshare.29609510.v1.

