# Supplementary material for: Neural mechanisms of visual quality perception and adaptability in the visual pathway
Source: Patterns (N Y). 2025 Oct 1;6(12):101368. doi: 10.1016/j.patter.2025.101368 (PMC12745989; doi:10.1016/j.patter.2025.101368)
Supplement: Document S1. Figures S1–S14, Tables S1–S5, supplemental methods, and supplemental notes [file mmc1.pdf]

Patterns, Volume *6*

Supplemental information

Neural mechanisms of visual quality perception and adaptability in the visual pathway

Yiming Zhang, Yitong Chen, Ying Hu, Xu Han, Zhenhui Xie, Xingrui Wang, Yan Zhou, Xiongkuo Min, and Guangtao Zhai

1 Supplemental figures

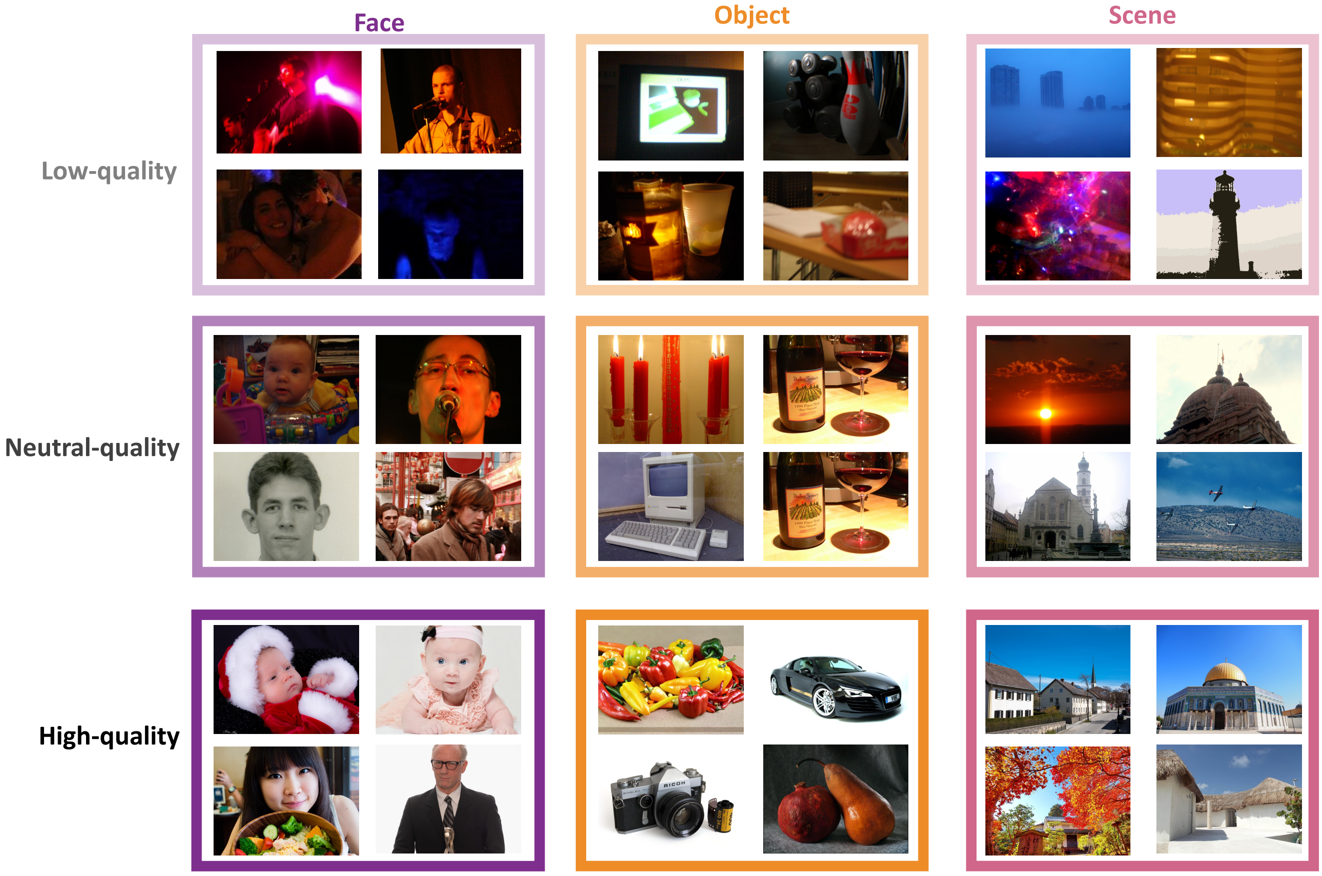

Figure S1: Visual stimulus examples from the fMRI experiments in this study.

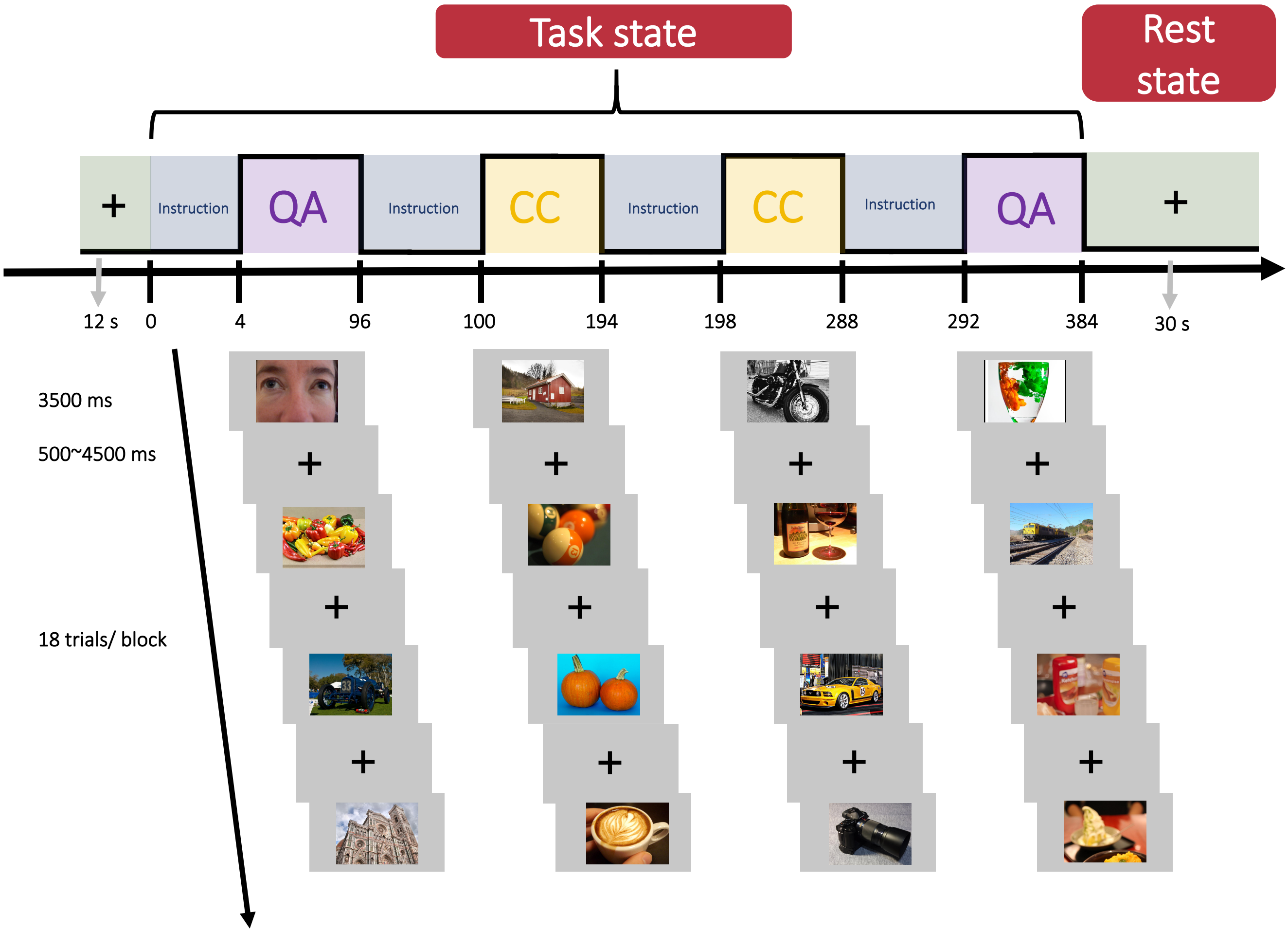

Figure S2: fMRI Experimental Design. Each run consists of four blocks, during which participants engage in either QA or CC tasks, inputting their responses via button press. Each block contains 18 trials, with each trial presenting an image for 3500 ms. During this period, participants are required to observe the image and complete the assigned task. The sequence of image presentation is randomized according to content category and quality level. The ISI is also randomized, ranging from 500 ms to 4500 ms. Each run begins with a 12-second fixation and ends with a 30-second fixation period, and the fMRI data from the final 30-second fixation are used as resting-state data for subsequent analysis. This is a schematic of the stimulus sequence for one of the runs. Each run consists of four blocks, during which participants engage in either QA or CC tasks, inputting their responses via button press. The counterbalancing of task block order across runs was implemented to ensure that the tasks were presented in a balanced manner across runs. Specifically, in odd numbered runs, the task block sequence followed a QA-CC-CC-QA pattern, while in even numbered runs, the order was reversed to CC-QA-QA-CC.

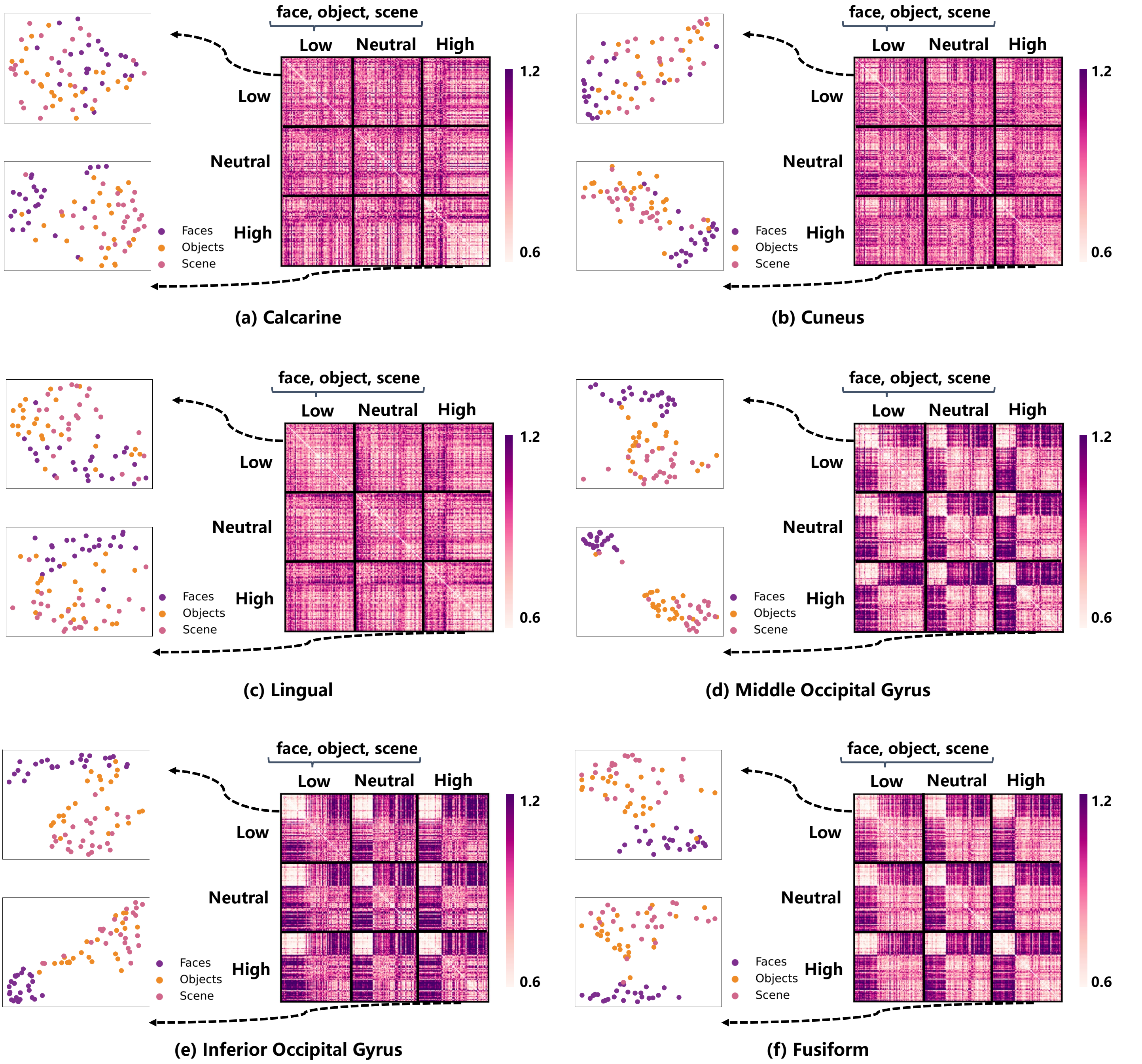

Figure S3: RDM and t-SNE in (a)calcarine, (b)cuneus, (c)lingual, (d)middle occipital gyrus, (e)inferior occipital gyrus, (f)fusiform gyrus On the right, the representation dissimilarity matrix (RDM) is displayed, organized by quality levels (low, neutral, high) and within each quality level by semantic category (face, object, scene). All presentational dissimilarity matrices in this approach are constructed by calculating the pairwise correlation distances between fMRI response patterns across all trials. On the left, the results of dimensionality reduction via the t-SNE algorithm for RDMs under low- and high-quality conditions are visualized, with image content categories used as labels.

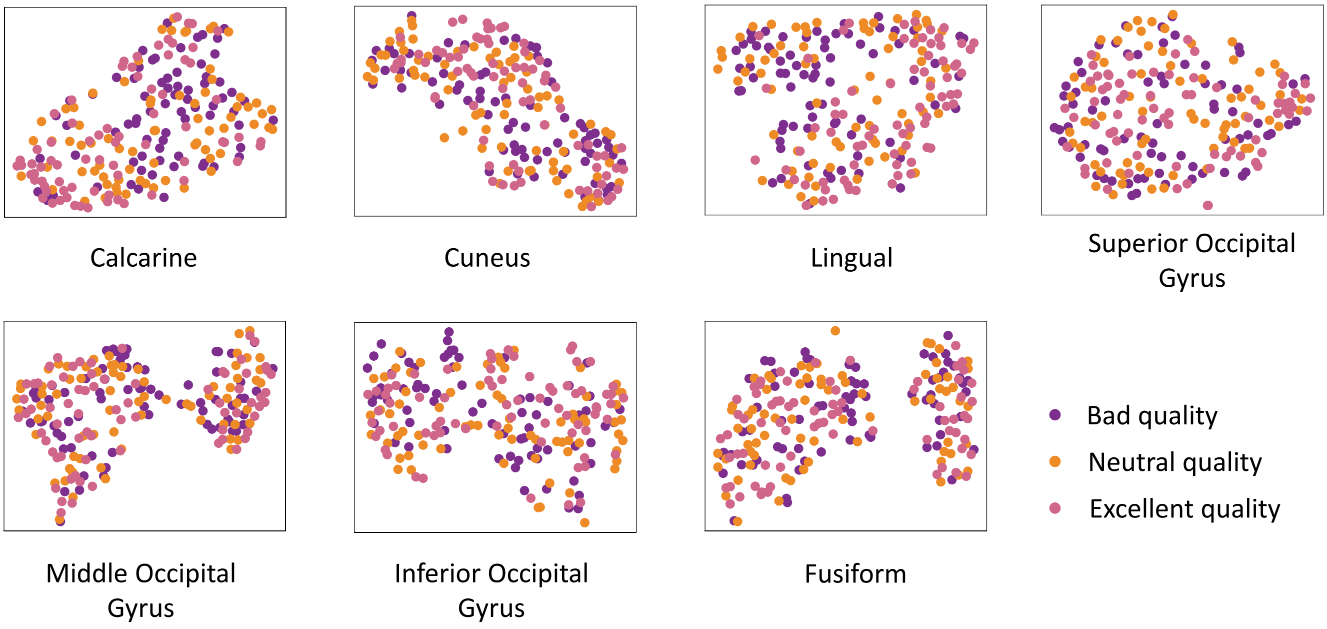

Figure S4: The results of dimensionality reduction via the t-SNE algorithm for RDMs in Fig. 3a (main text) and Figure S3 are visualized, with image percetion quality condition used as labels.

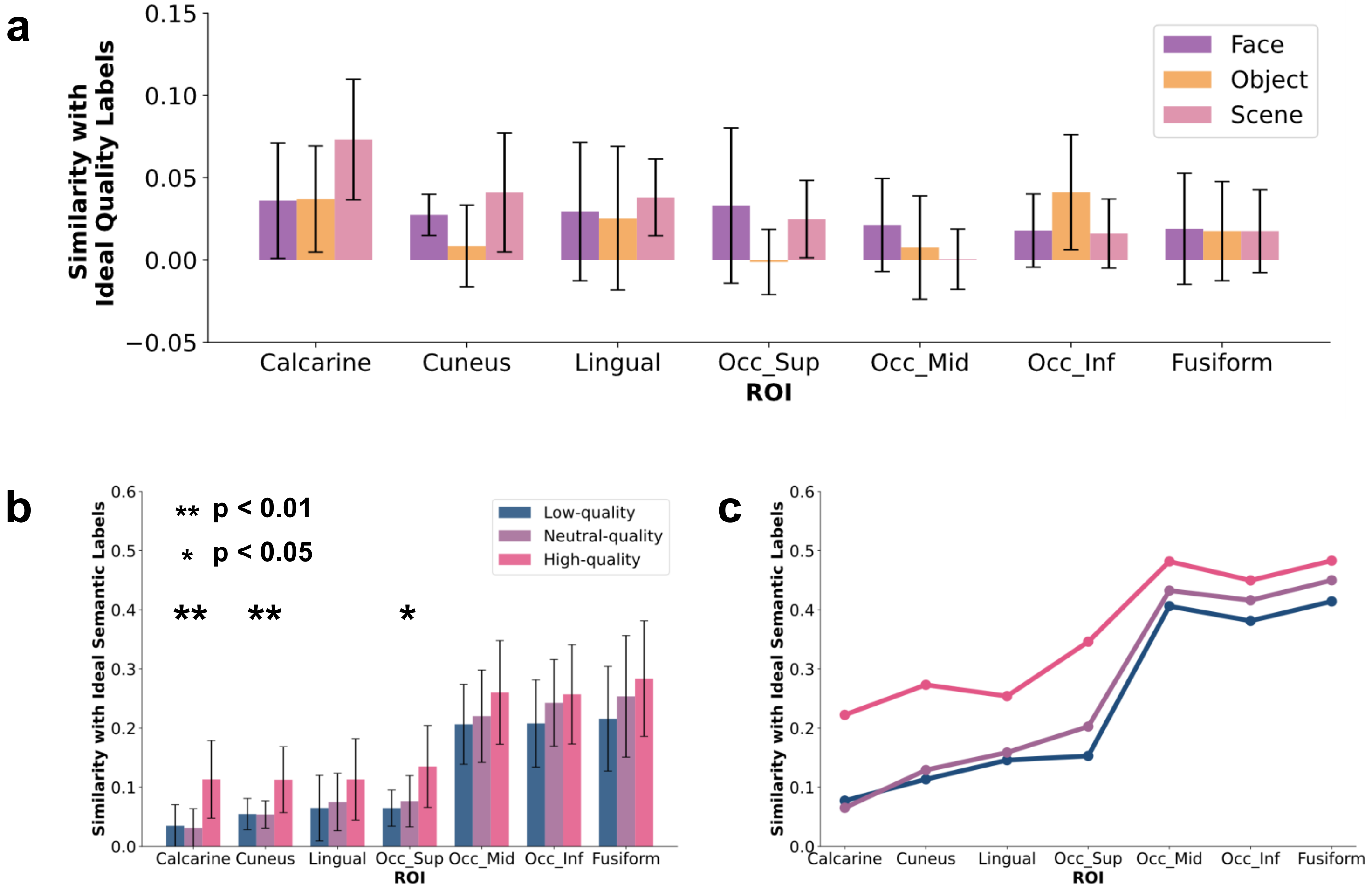

Figure S5: **(a)** The distribution of quality information content within each ROI across participants. No single ROI contains a rich and explicitly encoded representation of visual quality information. Results on similarity obtained using the Spearman correlation coefficient lead to conclusions consistent with those derived from the Pearson correlation. **(b)** The distribution of semantic information content within each ROI across low, neutral, and high-quality conditions among participants. Results on similarity obtained using the Spearman correlation coefficient lead to conclusions consistent with those derived from the Pearson correlation. Significant differences in the distribution of semantic information content across different quality conditions within each ROI are denoted with asterisks: * indicates $p<0.05$, and no asterisk indicates no significant difference in semantic information content across quality conditions within that ROI. **(c)** The average similarity between the RDMs of all participants and the standard semantic RDM is reported, reflecting the overall consistency in representing semantic content across the sample.

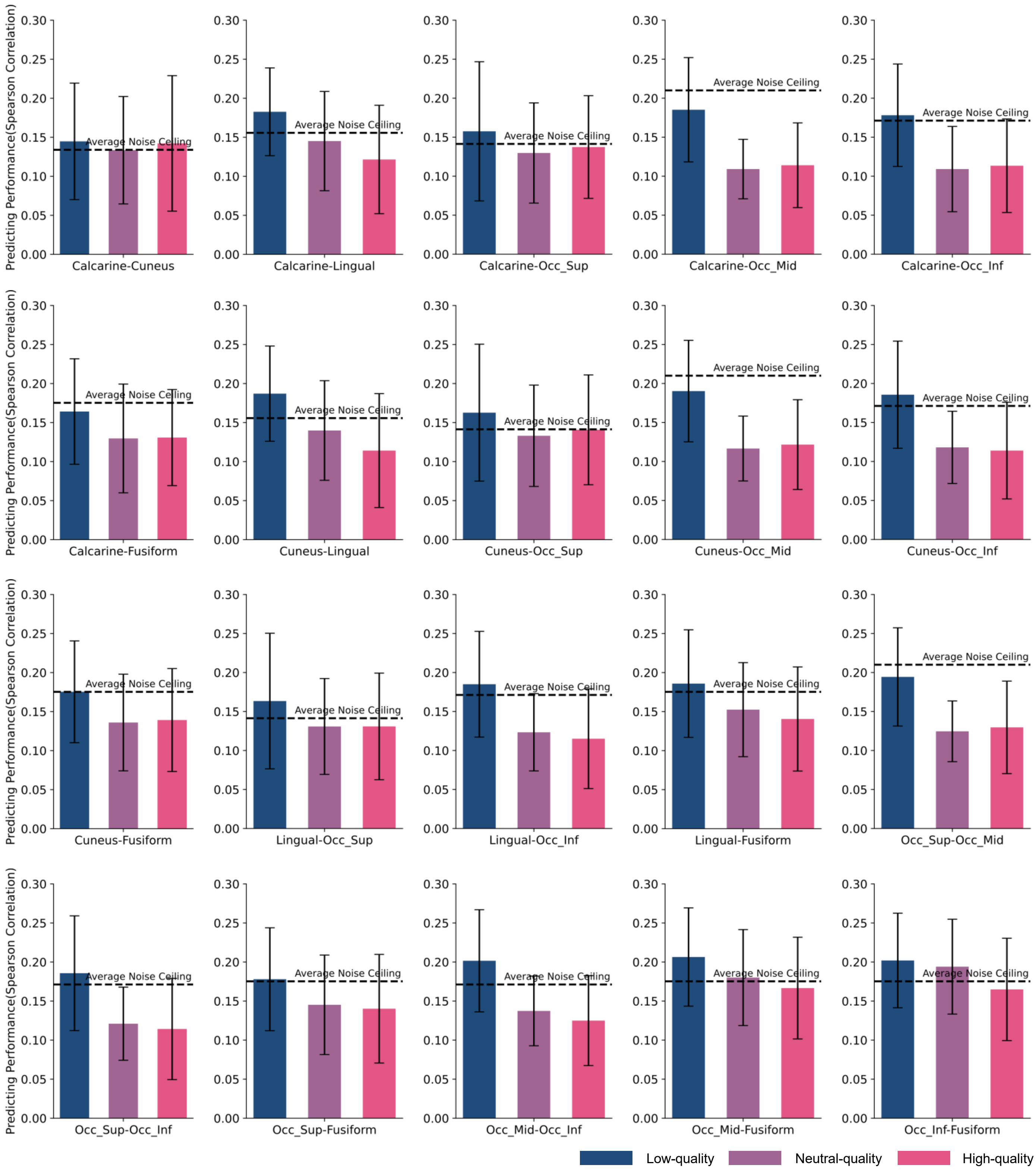

Figure S6: The prediction performance of the system for various ROI pairs, such as ‘A-B’, where B is predicted from A. The systems were trained exclusively on data from low-quality conditions and tested under high, neutral, and low-quality conditions. The performance means and standard deviations across 14 participants are presented.

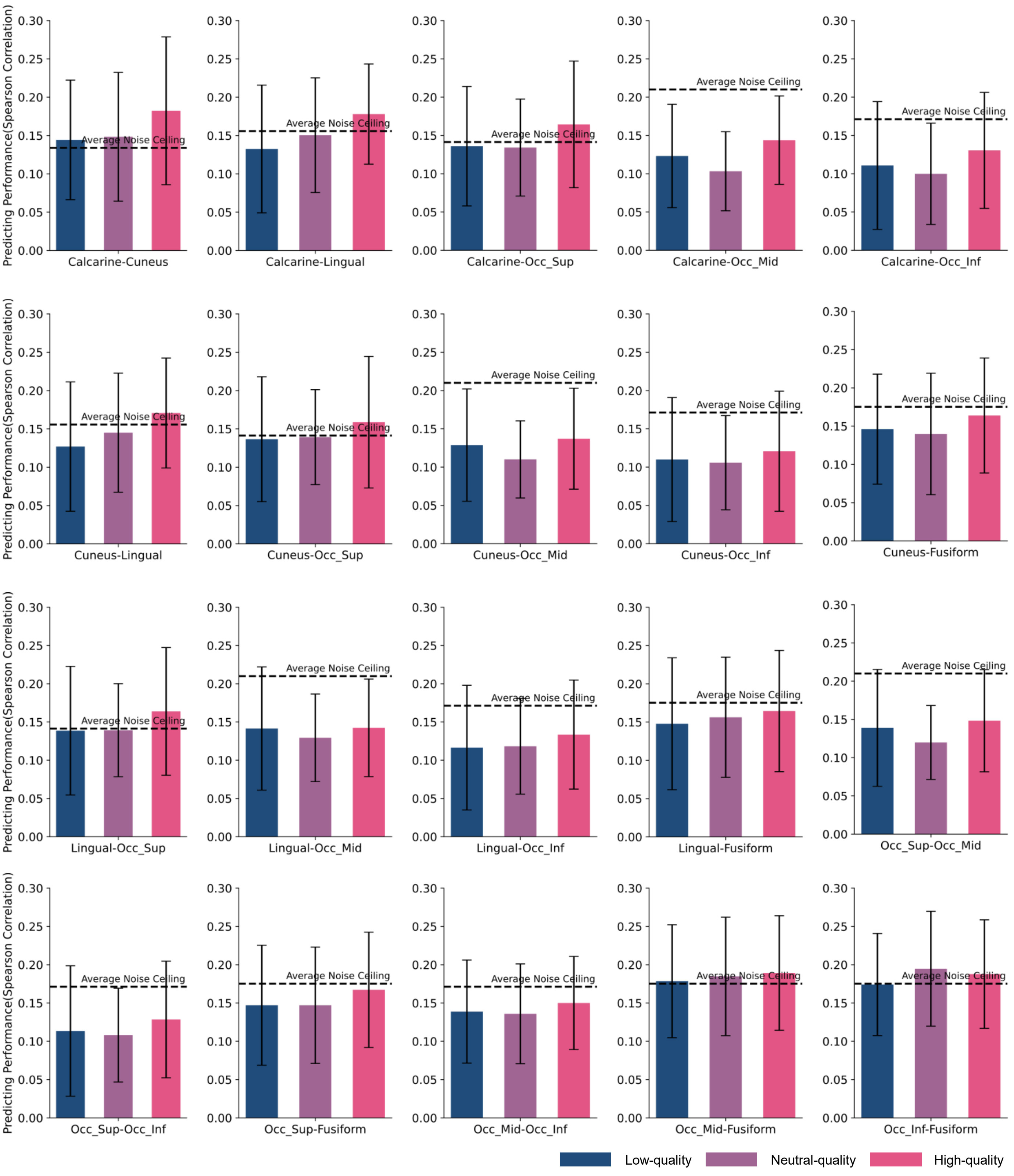

Figure S7: The prediction performance of the system for various ROI pairs, such as ‘A-B’, where B is predicted from A. The systems were trained exclusively on data from high-quality conditions and tested under high, neutral, and low-quality conditions. The performance means and standard deviations across 14 participants are presented.

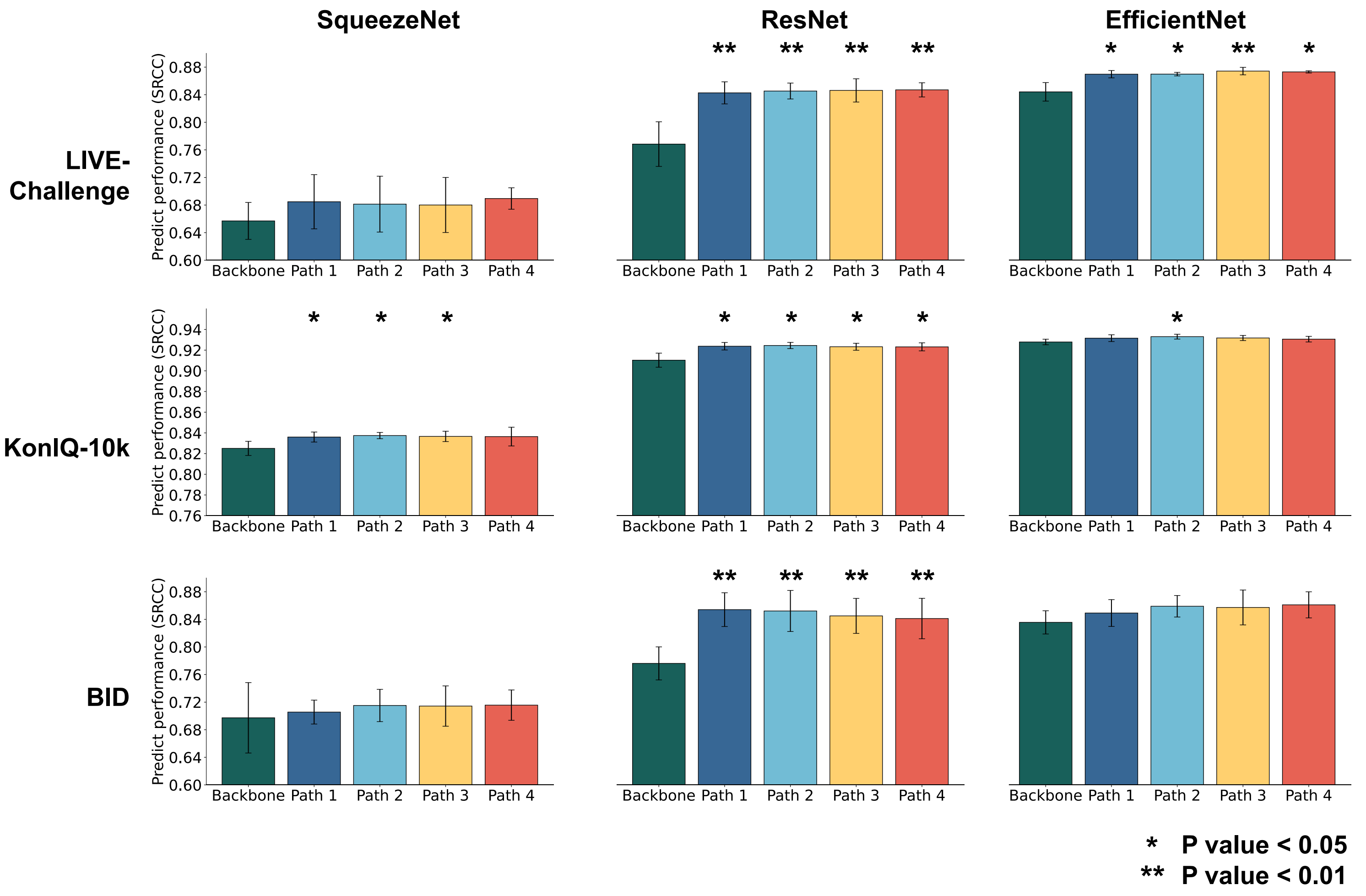

Figure S8: Performance comparison of the backbone and multi-path configurations (Path 1-4) across three benchmark datasets: LIVE-Challenge, Koniq-10k, and BID, using different backbone architectures (SqueezeNet, ResNet, and EfficientNet). The Spearman correlation coefficient (SRCC) values demonstrate the consistent improvement achieved by integrating features from Path 1-4, with significant performance gains over the backbone alone. Statistical significance is marked as * (p<0.05) and ** (p<0.01). The error bars represent the variance from 5-fold cross-validation. Path 2, Path 3, and Path 4 exhibit particularly strong performance across datasets, indicating the effectiveness of multi-scale feature integration in visual quality prediction.

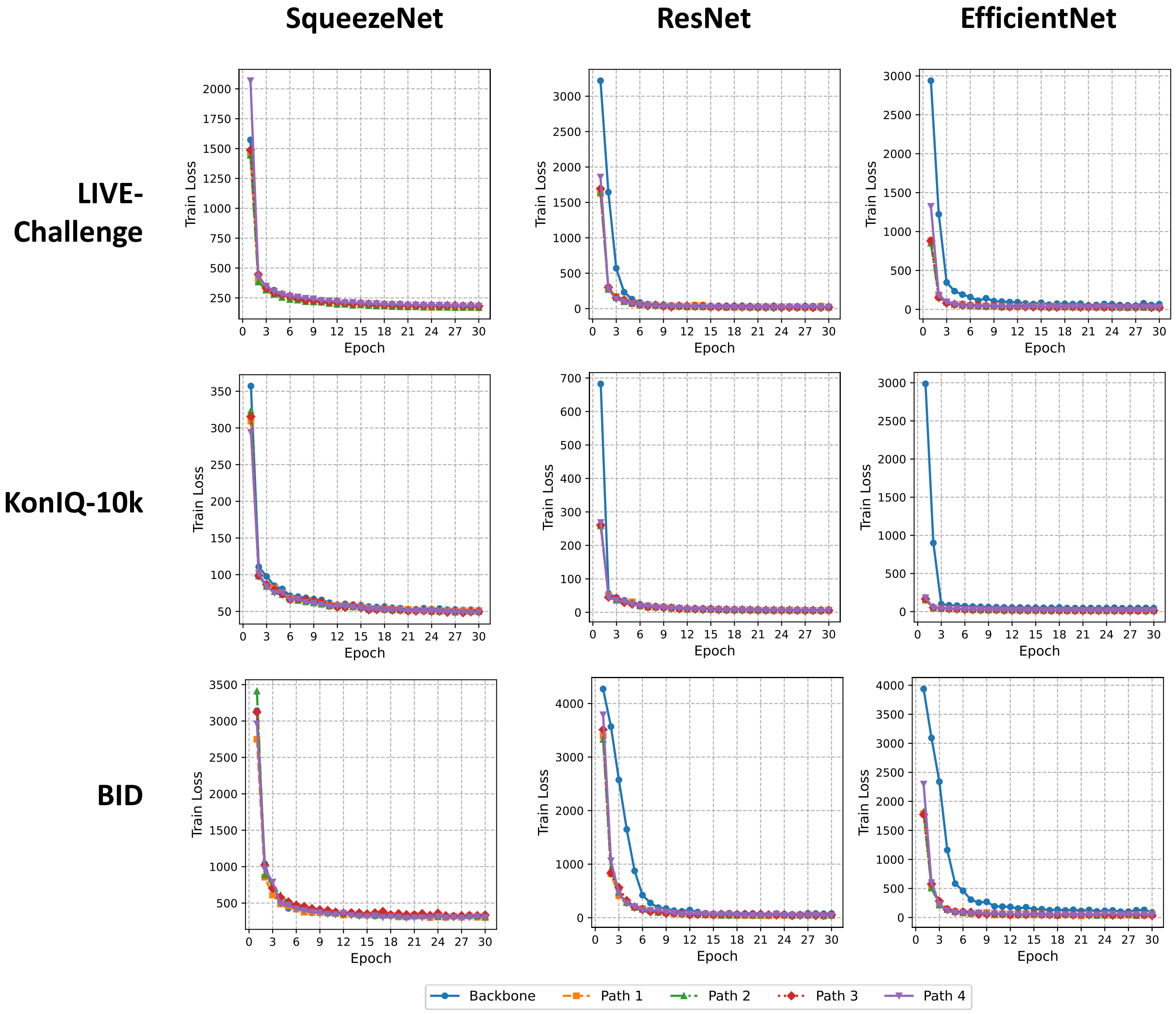

Figure S9: The loss curve for each network on each dataset

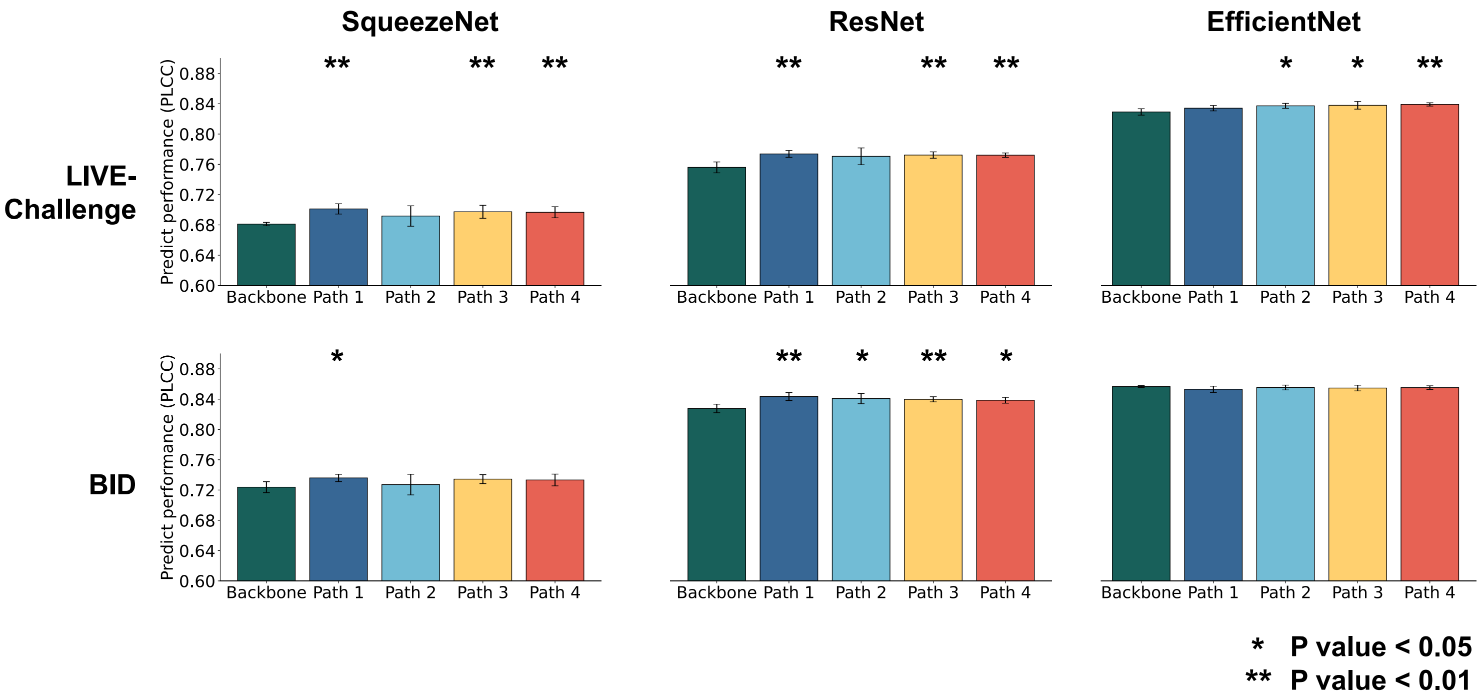

Figure S10: Cross-datasets performance comparison of the backbone and multi-path configurations (Path 1-4) on the LIVE-Challenge and BID datasets. All networks were pre-trained on the Koniq-10k dataset. The results illustrate the effectiveness of different path configurations in enhancing model performance across diverse image quality assessment tasks. The Pearson's Linear Correlation Coefficient (PLCC) values demonstrate the consistent improvement achieved by integrating features from Path 1-4, with significant performance gains over the backbone alone. Statistical significance is marked as * ($p<0.05$) and ** ($p<0.01$). The error bars represent the variance resulting from the 5-fold partitioning of the Koniq-10k dataset, where the models are pre-trained on the respective training sets and subsequently tested on the full test sets of LIVE-Challenge and BID five times. Path 2, Path 3, and Path 4 exhibit particularly strong performance across datasets, indicating the effectiveness of multi-scale feature integration in visual quality prediction.

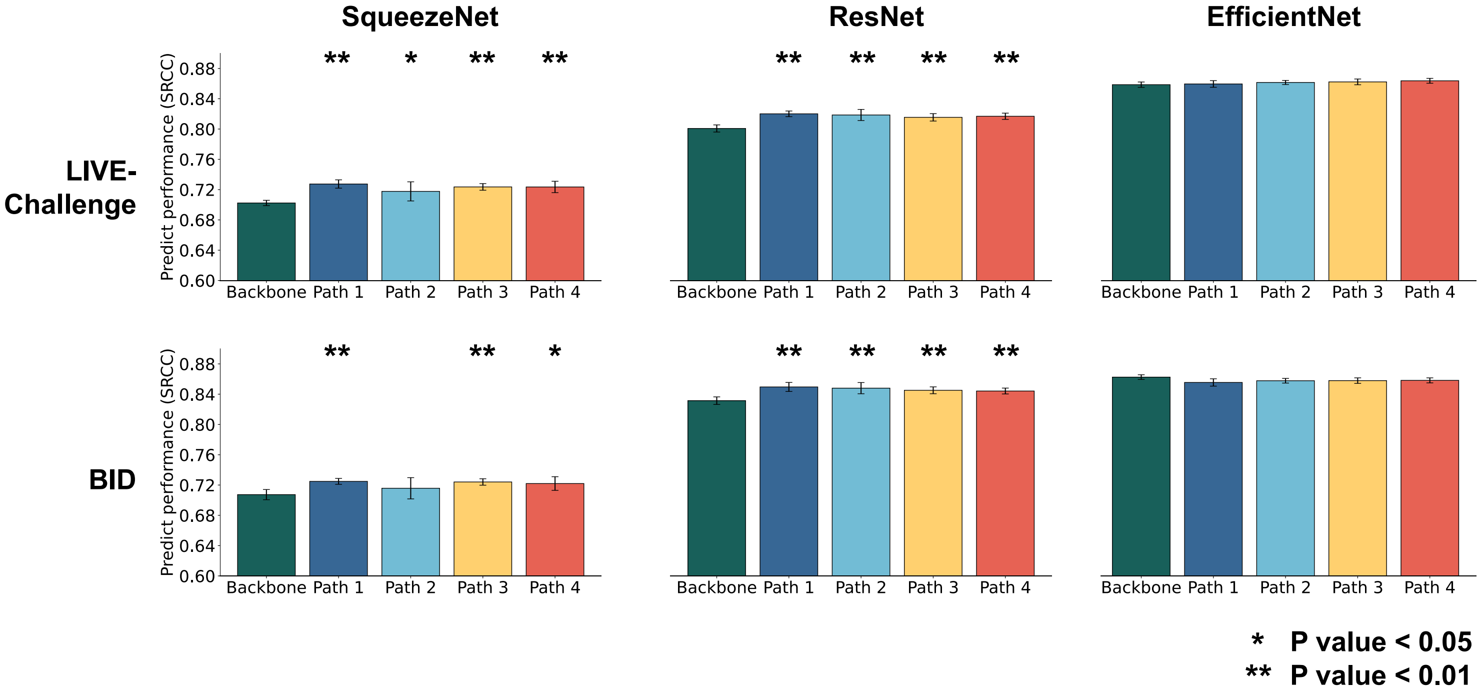

Figure S11: Cross-datasets performance comparison of the backbone and multi-path configurations (Path 1-4) on the LIVE-Challenge and BID datasets. All networks were pre-trained on the Koniq-10k dataset. The results illustrate the effectiveness of different path configurations in enhancing model performance across diverse image quality assessment tasks. The Spearman correlation coefficient (SRCC) values demonstrate the consistent improvement achieved by integrating features from Path 1-4, with significant performance gains over the backbone alone. Statistical significance is marked as * ($p<0.05$) and ** ($p<0.01$). The error bars represent the variance resulting from the 5-fold partitioning of the Koniq-10k dataset, where the models are pre-trained on the respective training sets and subsequently tested on the full test sets of LIVE-Challenge and BID five times. Path 2, Path 3, and Path 4 exhibit particularly strong performance across datasets, indicating the effectiveness of multi-scale feature integration in visual quality prediction.

Squeezenet = nn.Sequential(*list(models.squeezenet1_0(weights='DEFAULT').children()))[0]

	Squeezenet[0][2] MaxPool2d	Squeezenet[0][4] ['expand3x3_activation'] ReLU	Squeezenet[0][7] ['expand3x3_activation'] ReLU	Squeezenet[0][9] ['expand3x3_activation'] ReLU	Squeezenet[0][12] ['expand3x3_activation'] ReLU
Path 1				√	√
Path 2			√	√	√
Path 3		√	√	√	√
Path 4	√	√	√	√	√

Resnet50 = nn.Sequential(*list(models.resnet50(weights='DEFAULT').children()))

	Resnet[3] MaxPool2d	Resnet[4][2] ['relu'] ReLU	Resnet[5][3] ['relu'] ReLU	Resnet[6][5] ['relu'] ReLU	Resnet[7][2] ['relu'] ReLU
Path 1				√	√
Path 2			√	√	√
Path 3		√	√	√	√
Path 4	√	√	√	√	√

Efficientnet = nn.Sequential(*list(models.efficientnet_v2_m(weights='DEFAULT').children()))[0]

	Efficientnet[2][4] [‘stochastic_depth’] StochasticDepth	Efficientnet[3][4] [‘stochastic_depth’] StochasticDepth	Efficientnet[5][13] [‘stochastic_depth’] StochasticDepth	Efficientnet[6][17] [‘stochastic_depth’] StochasticDepth	Efficientnet[7][4] [‘stochastic_depth’] StochasticDepth
Path 1				√	√
Path 2			√	√	√
Path 3		√	√	√	√
Path 4	√	√	√	√	√

Figure S12: The features involved in multi-layer feature fusion across each path in the neural network. The experiments were conducted using PyTorch, with all backbone architectures utilizing the default structures provided by PyTorch.

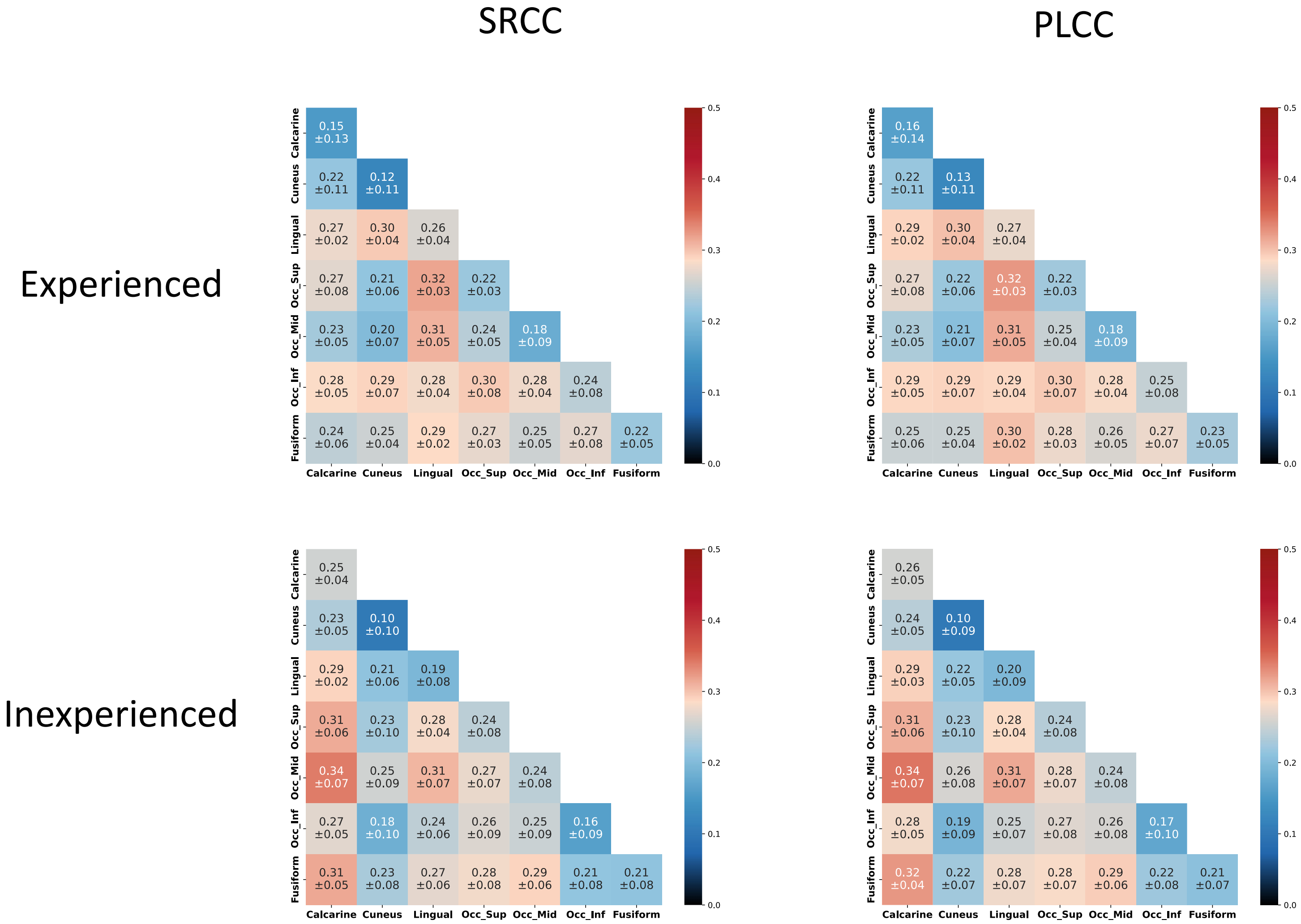

Figure S13: Single-subject quality information decoding results include the mean and variance among experienced and inexperienced subjects of the SRCC and PLCC between the regression-predicted quality scores from the combined representation vectors of the ROIs (as indicated on the x-axis and y-axis) and the ground truth. Higher SRCC and PLCC values indicate greater consistency between the predicted quality information and the quality labels.

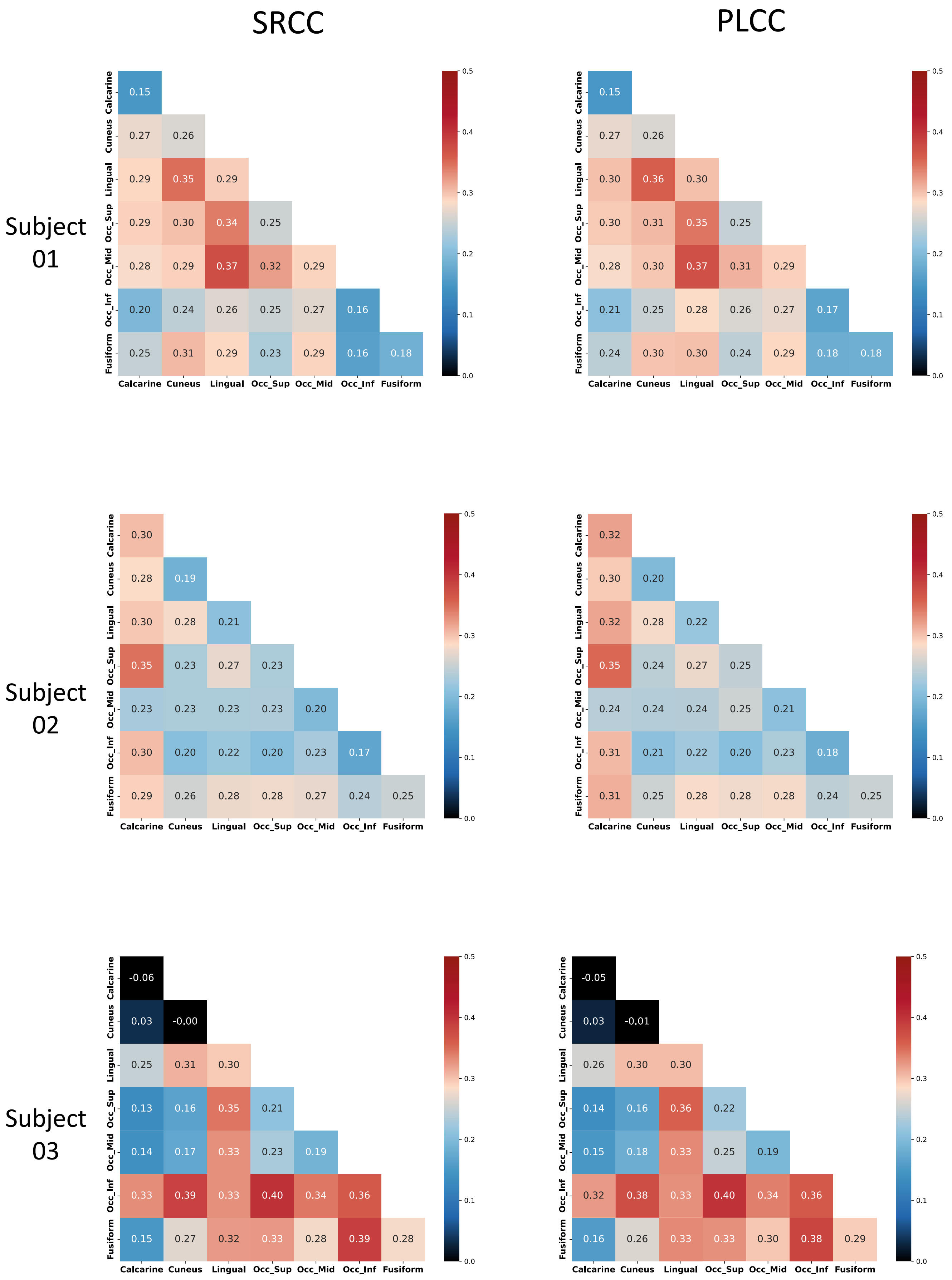
SRCC
PLCC
Subject 01
Subject 02
Subject 03
Calcarine
Cuneus
Lingual
Occ_Sup
Occ_Mid
Occ_Inf
Fusiform
0.15
0.27 0.26
0.29 0.35 0.29
0.29 0.30 0.34 0.25
0.28 0.29 0.37 0.32 0.29
0.20 0.24 0.26 0.25 0.27 0.16
0.25 0.31 0.29 0.23 0.29 0.16 0.18
0.15
0.27 0.26
0.30 0.36 0.30
0.30 0.31 0.35 0.25
0.28 0.30 0.37 0.31 0.29
0.21 0.25 0.28 0.26 0.27 0.17
0.24 0.30 0.30 0.24 0.29 0.18 0.18
0.30
0.28 0.19
0.30 0.28 0.21
0.35 0.23 0.27 0.23
0.23 0.23 0.23 0.23 0.20
0.30 0.20 0.22 0.20 0.23 0.17
0.29 0.26 0.28 0.28 0.27 0.24 0.25
0.32
0.30 0.20
0.32 0.28 0.22
0.35 0.24 0.27 0.25
0.24 0.24 0.24 0.25 0.21
0.31 0.21 0.22 0.20 0.23 0.18
0.31 0.25 0.28 0.28 0.28 0.24 0.25
-0.06
0.03 -0.00
0.25 0.31 0.30
0.13 0.16 0.35 0.21
0.14 0.17 0.33 0.23 0.19
0.33 0.39 0.33 0.40 0.34 0.36
0.15 0.27 0.32 0.33 0.28 0.39 0.28
-0.05
0.03 -0.01
0.26 0.30 0.30
0.14 0.16 0.36 0.22
0.15 0.18 0.33 0.25 0.19
0.32 0.38 0.33 0.40 0.34 0.36
0.16 0.26 0.33 0.33 0.30 0.38 0.29
0.0
0.1
0.2
0.3
0.4
0.5

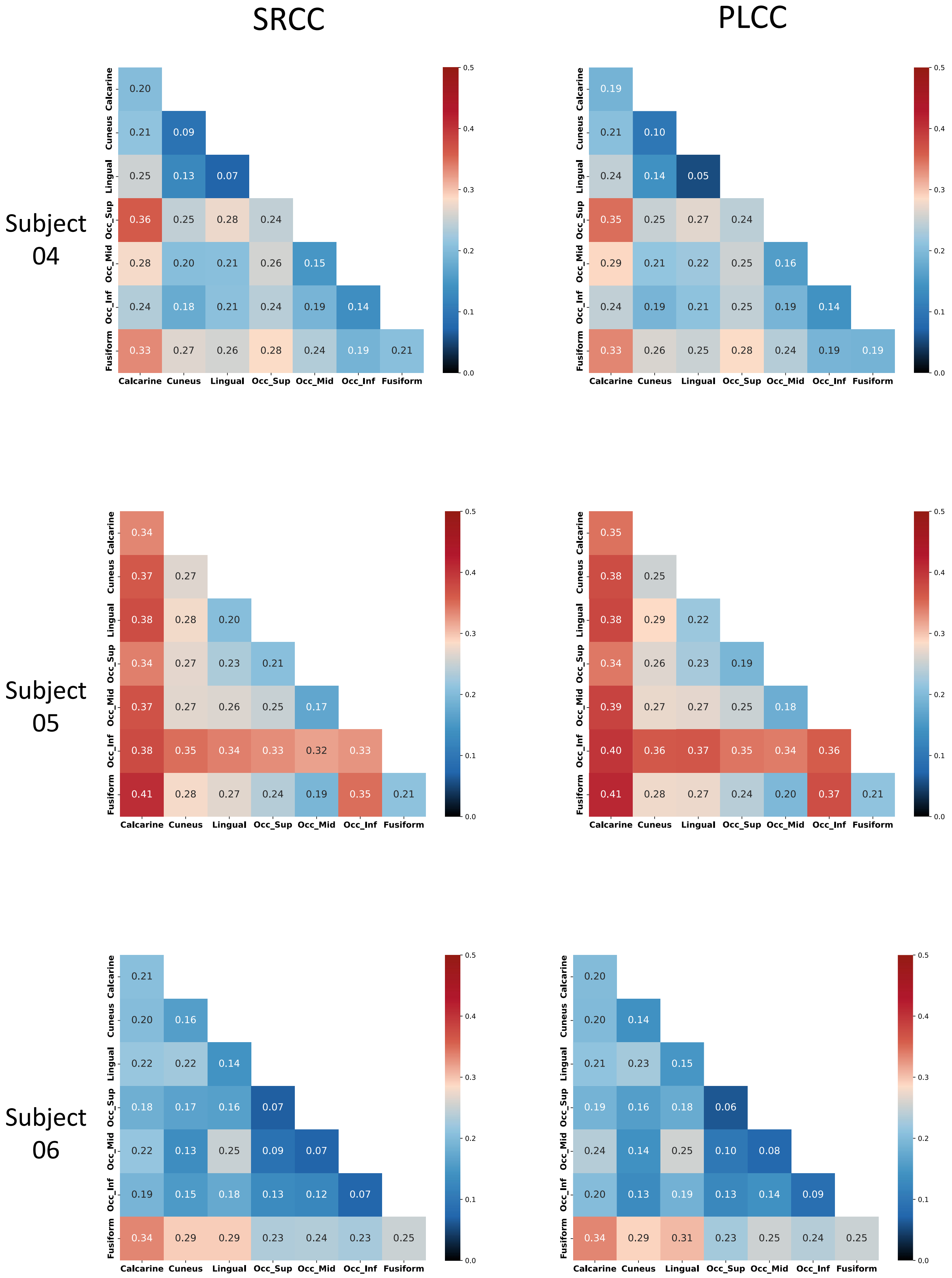
SRCC
PLCC
Subject 04
Subject 05
Subject 06
Calcarine
Cuneus
Lingual
Occ_Sup
Occ_Mid
Occ_Inf
Fusiform
0.0
0.1
0.2
0.3
0.4
0.5
0.20
0.21 0.09
0.25 0.13 0.07
0.36 0.25 0.28 0.24
0.28 0.20 0.21 0.26 0.15
0.24 0.18 0.21 0.24 0.19 0.14
0.33 0.27 0.26 0.28 0.24 0.19 0.21
0.19
0.21 0.10
0.24 0.14 0.05
0.35 0.25 0.27 0.24
0.29 0.21 0.22 0.25 0.16
0.24 0.19 0.21 0.25 0.19 0.14
0.33 0.26 0.25 0.28 0.24 0.19 0.19
0.34
0.37 0.27
0.38 0.28 0.20
0.34 0.27 0.23 0.21
0.37 0.27 0.26 0.25 0.17
0.38 0.35 0.34 0.33 0.32 0.33
0.41 0.28 0.27 0.24 0.19 0.35 0.21
0.35
0.38 0.25
0.38 0.29 0.22
0.34 0.26 0.23 0.19
0.39 0.27 0.27 0.25 0.18
0.40 0.36 0.37 0.35 0.34 0.36
0.41 0.28 0.27 0.24 0.20 0.37 0.21
0.21
0.20 0.16
0.22 0.22 0.14
0.18 0.17 0.16 0.07
0.22 0.13 0.25 0.09 0.07
0.19 0.15 0.18 0.13 0.12 0.07
0.34 0.29 0.29 0.23 0.24 0.23 0.25
0.20
0.20 0.14
0.21 0.23 0.15
0.19 0.16 0.18 0.06
0.24 0.14 0.25 0.10 0.08
0.20 0.13 0.19 0.13 0.14 0.09
0.34 0.29 0.31 0.23 0.25 0.24 0.25

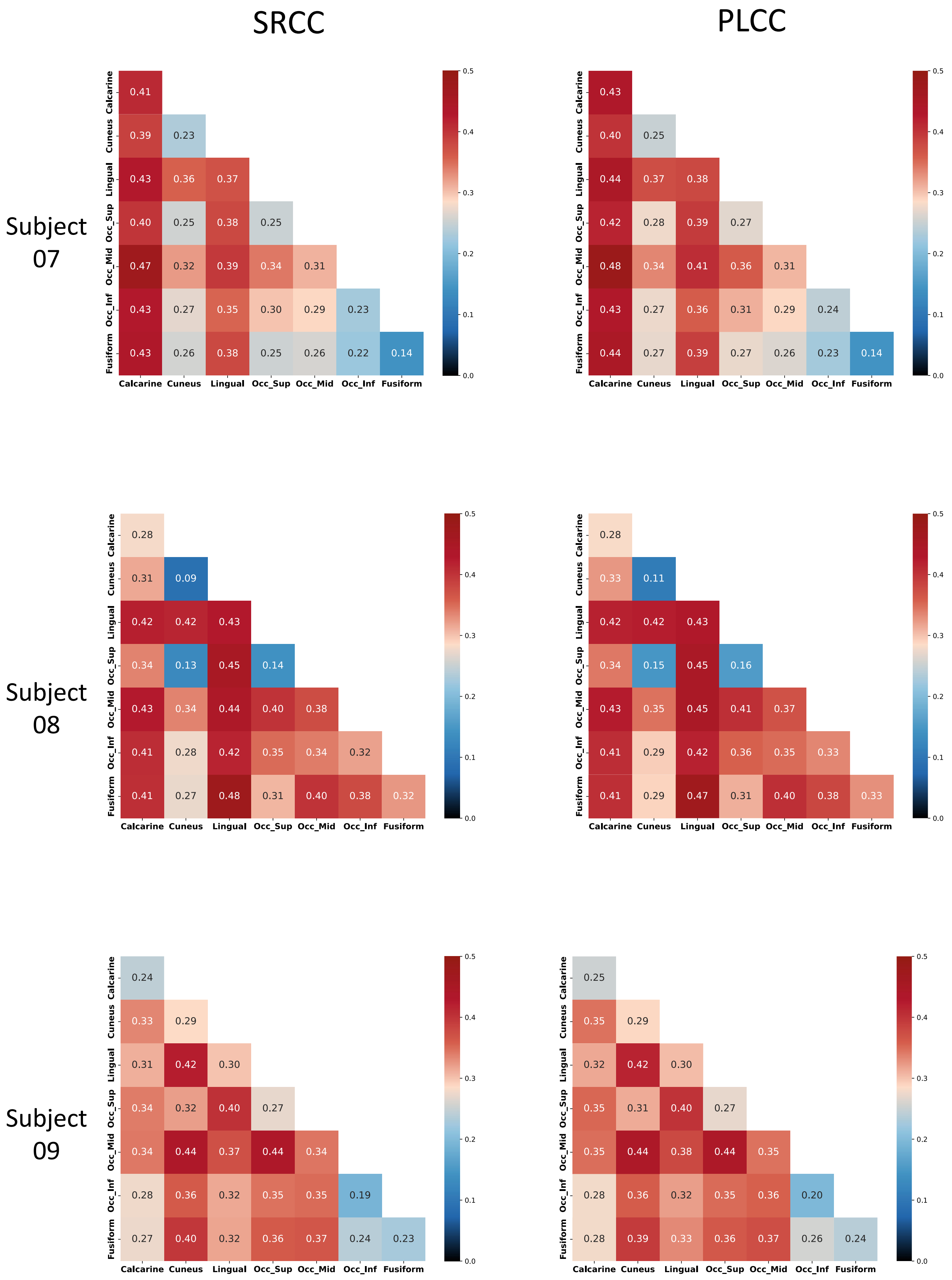
SRCC
PLCC
Subject 07
Subject 08
Subject 09
Calcarine
Cuneus
Lingual
Occ_Sup
Occ_Mid
Occ_Inf
Fusiform
0.41
0.39 0.23
0.43 0.36 0.37
0.40 0.25 0.38 0.25
0.47 0.32 0.39 0.34 0.31
0.43 0.27 0.35 0.30 0.29 0.23
0.43 0.26 0.38 0.25 0.26 0.22 0.14
0.43
0.40 0.25
0.44 0.37 0.38
0.42 0.28 0.39 0.27
0.48 0.34 0.41 0.36 0.31
0.43 0.27 0.36 0.31 0.29 0.24
0.44 0.27 0.39 0.27 0.26 0.23 0.14
0.28
0.31 0.09
0.42 0.42 0.43
0.34 0.13 0.45 0.14
0.43 0.34 0.44 0.40 0.38
0.41 0.28 0.42 0.35 0.34 0.32
0.41 0.27 0.48 0.31 0.40 0.38 0.32
0.28
0.33 0.11
0.42 0.42 0.43
0.34 0.15 0.45 0.16
0.43 0.35 0.45 0.41 0.37
0.41 0.29 0.42 0.36 0.35 0.33
0.41 0.29 0.47 0.31 0.40 0.38 0.33
0.24
0.33 0.29
0.31 0.42 0.30
0.34 0.32 0.40 0.27
0.34 0.44 0.37 0.44 0.34
0.28 0.36 0.32 0.35 0.35 0.19
0.27 0.40 0.32 0.36 0.37 0.24 0.23
0.25
0.35 0.29
0.32 0.42 0.30
0.35 0.31 0.40 0.27
0.35 0.44 0.38 0.44 0.35
0.28 0.36 0.32 0.35 0.36 0.20
0.28 0.39 0.33 0.36 0.37 0.26 0.24
0.0 0.1 0.2 0.3 0.4 0.5

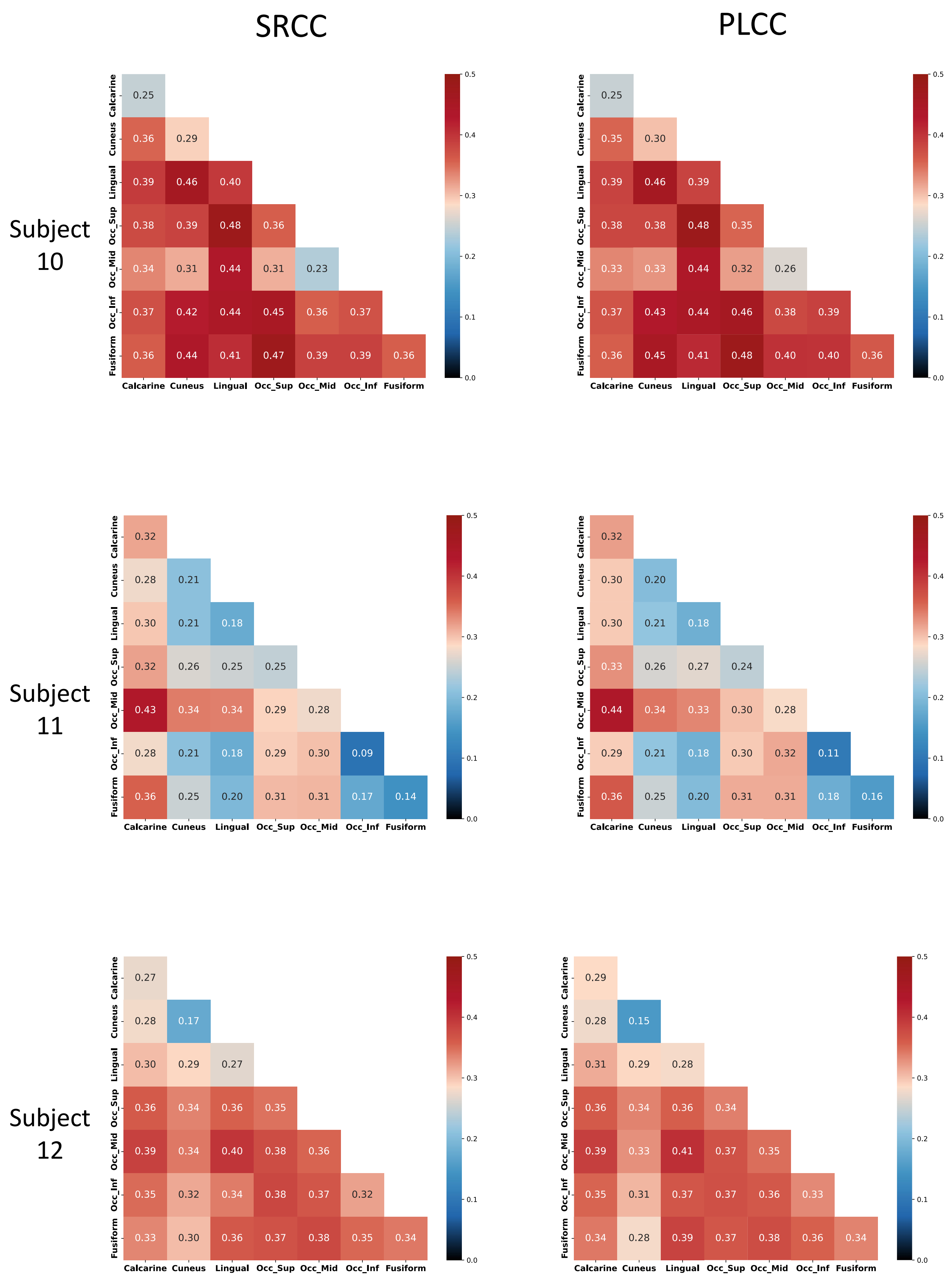
SRCC
PLCC
Subject 10
Subject 11
Subject 12
Calcarine
Cuneus
Lingual
Occ_Sup
Occ_Mid
Occ_Inf
Fusiform
0.25
0.36 0.29
0.39 0.46 0.40
0.38 0.39 0.48 0.36
0.34 0.31 0.44 0.31 0.23
0.37 0.42 0.44 0.45 0.36 0.37
0.36 0.44 0.41 0.47 0.39 0.39 0.36
0.25
0.35 0.30
0.39 0.46 0.39
0.38 0.38 0.48 0.35
0.33 0.33 0.44 0.32 0.26
0.37 0.43 0.44 0.46 0.38 0.39
0.36 0.45 0.41 0.48 0.40 0.40 0.36
0.32
0.28 0.21
0.30 0.21 0.18
0.32 0.26 0.25 0.25
0.43 0.34 0.34 0.29 0.28
0.28 0.21 0.18 0.29 0.30 0.09
0.36 0.25 0.20 0.31 0.31 0.17 0.14
0.32
0.30 0.20
0.30 0.21 0.18
0.33 0.26 0.27 0.24
0.44 0.34 0.33 0.30 0.28
0.29 0.21 0.18 0.30 0.32 0.11
0.36 0.25 0.20 0.31 0.31 0.18 0.16
0.27
0.28 0.17
0.30 0.29 0.27
0.36 0.34 0.36 0.35
0.39 0.34 0.40 0.38 0.36
0.35 0.32 0.34 0.38 0.37 0.32
0.33 0.30 0.36 0.37 0.38 0.35 0.34
0.29
0.28 0.15
0.31 0.29 0.28
0.36 0.34 0.36 0.34
0.39 0.33 0.41 0.37 0.35
0.35 0.31 0.37 0.37 0.36 0.33
0.34 0.28 0.39 0.37 0.38 0.36 0.34
0.0
0.1
0.2
0.3
0.4
0.5

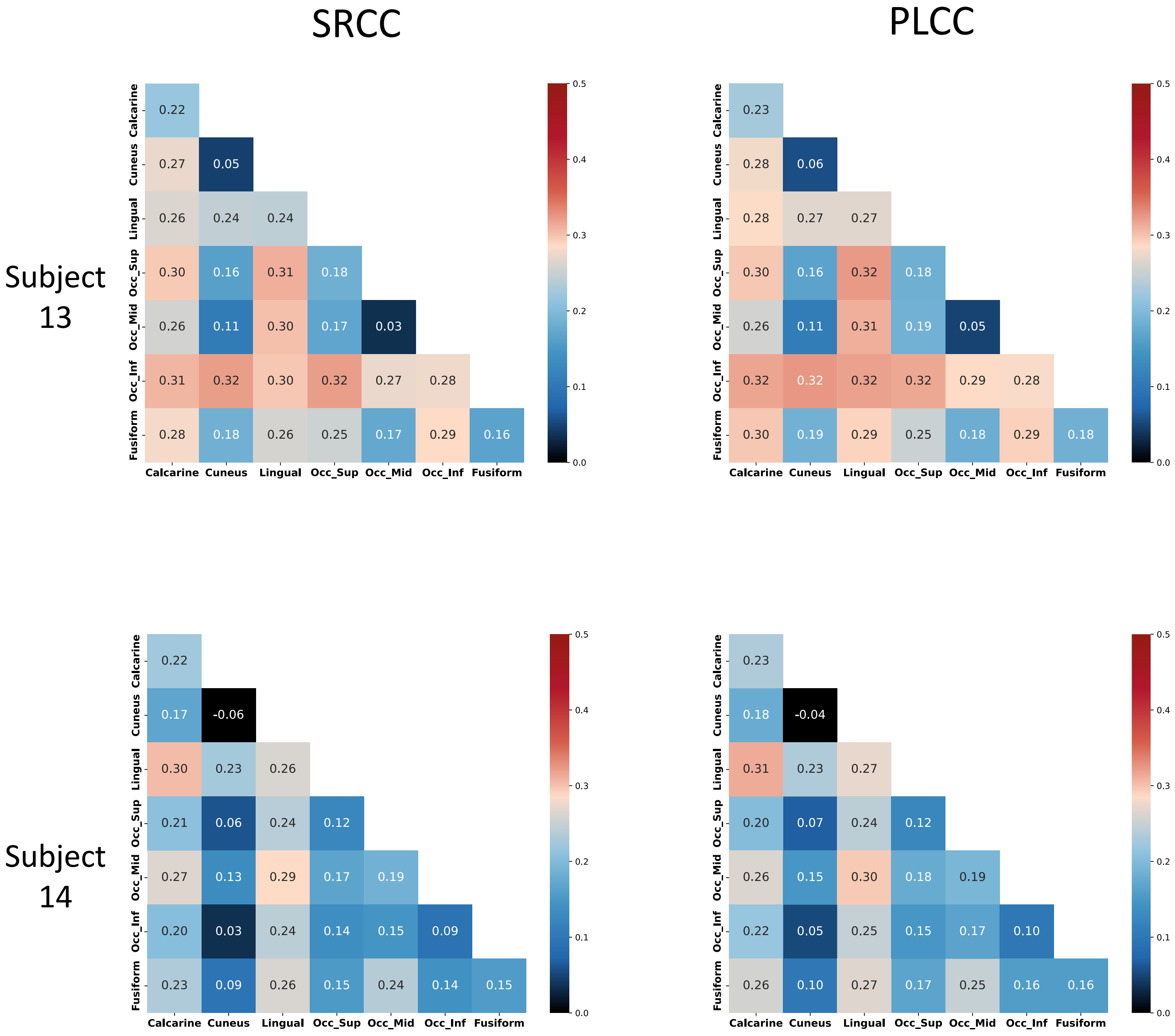

Figure S14: Quality information decoding results of each subject. The SRCC and PLCC between the regression-predicted quality scores from the combined representation vectors of the ROIs (as indicated on the x-axis and y-axis) and the ground truth are shown. Higher SRCC and PLCC values indicate greater consistency between the predicted quality information and the quality labels.

2 Supplemental tables

Table S1: Regions activated and inhibited under the quality assessment task in contrast to content recognition. Results of univariate analyses from SPM ($p{<}0.001$, FDR corrected at voxel level ($p{<}0.05$)) shown below include cluster sizes, peak level t-scores, and the MNI coordinates of each peak and their corresponding atlas labels based on Neuromorphometrics and Brodmann's area (BA) from whole-brain analysis. We also report the main structures contained in large clusters covering multiple brain regions.

Contrast	Cluster size	Main structure in cluster	Peak MNI coordinates	Brain region	Hemisphere	BA	t-score
QA>CC	401	Middle Occipital Gyrus, Calcarine, Cuneus, Inferior Occipital Gyrus	(34, -92, -6)	Inferior Occipital Gyrus	R	–	8.85
			(28,-96,10)	Middle Occipital Gyrus	R	–	7.99
			(28, -90, 2)	Middle Occipital Gyrus	R	–	7.45
	457	Middle Occipital Gyrus, Calcarine, Cuneus, Inferior Occipital Gyrus	(-22, -102, 4)	Middle Occipital Gyrus	L	18	7.52
			(-44, -81, -10)	Inferior Occipital Gyrus	L	19	6.63
			(-36, -96, -2)	Middle Occipital Gyrus	L	18	6.12
	24	–	(54, 14, 26)	Inferior Frontal Gyrus	R	–	5.54
	61	–	(38, -78, -12)	Inferior Occipital Gyrus	R	19	5.47
	48	–	(38,-2,12)	Lingual	L	–	5.18
	21	–	(-36, -66, -12)	Fusiform	L	–	4.82
	36	–	(28, -76, 34)	Superior Occipital Gyrus	R	19	4.78
	31	–	(40, -60, 12)	Fusiform	R	–	4.60

Table S2: Regions activated and inhibited under the high-quality condition in contrast to low-quality condition. Results of univariate analyses from SPM (p<0.001, FDR corrected at voxel level (p<0.05)) shown below include cluster sizes, peak level t-scores, and the MNI coordinates of each peak and their corresponding atlas labels based on Neuromorphometrics and Brodmann's area (BA) from whole-brain analysis. For large clusters covering multiple brain regions, we also report the main structures contained.

Contrast	Cluster size	Main structure in cluster	Peak MNI coordinates	Brain region	Hemisphere	BA	t-score
Excellent> Bad	1946	Postcentral Gyrus, Precuneus, Superior Parietal Lobule	(-40,-24,44)	Postcentral Gyrus	L	4	15.65
			(-36,-24,58)	Precentral Gyrus		–	11.48
			(-38,-22,66)			3	10.28
	139	Postcentral Gyrus, Insula, Superior Temporal Gyrus	(-46,-22,22)	Insula	L	–	9.51
			(-56,-26,22)	Postcentral Gyrus		–	5.90
			(-50,-20,8)	Superior Temporal Gyrus		–	5.22
	445	Cuneus, Middle Occipital Gyrus, Lingual Gyrus, BA17, Inferior Occipital Gyrus, BA18	(30,-98,-6)	Middle Occipital Gyrus	R	18	9.25
			(14,-94,-10)	Lingual Gyrus		–	7.41
			(26,-92,-14)	Inferior Occipital Gyrus		–	5.22
	540	Cuneus, Middle Occipital Gyrus, Lingual Gyrus, BA17, Inferior Occipital Gyrus, BA18	(-24,-96,-10)	Lingual Gyrus	L	–	8.20
			(-12,-100,-10)	Lingual Gyrus		18	7.76
			(-24,-100,-10)	Middle Occipital Gyrus		–	7.68
	154	Paracentral Lobule, Parietal Lobe, Precuneus, Cingulate Gyrus	(-14,-38,52)	Paracentral Lobule	L	5	6.81
			(-6,-30,42)	Cingulate Gyrus		31	5.69
			(-4,-22,46)	Paracentral Lobule		31	5.53
	158	–	(62,-42,36)	Inferior Parietal Lobule	R	40	7.18
	96	–	(-64,-38,34)	Inferior Parietal Lobule	L	40	6.71
	20	–	(-44,4,-6)	Insula	L	–	5.99
	29	–	(14,-32,40)	Cingulate Gyrus	R	31	5.95
Bad> Excellent	2826	Postcentral Gyrus, Superior Parietal Lobule, Middle Frontal Gyrus, Precentral Gyrus, BA4, 3, 6, 40, 19	(34,-26,62)	Precentral Gyrus	R	4	15.76
			(50,-14,52)			–	14.85
			(40,-20,62)			4	14.05
	679	Middle Occipital Gyrus, BA37, BA39, Middle Temporal Gyrus, Fusiform, Inferior Temporal Gyrus	(-48,-84,2)	Middle Occipital Gyrus	L	–	12.62
			(-46,-76,4)			19	9.60
			(-44,-76,-4)			–	8.29
	918	Middle Occipital Gyrus, BA37, BA19, Inferior Temporal Gyrus, Fusiform Gyrus, Middle Temporal Gyrus	(42,-74,-4)	Inferior Temporal Gyrus	R	–	11.53
			(50,-72,0)	Middle Occipital Gyrus		37	9.11
			(52,-72,12)	Middle Temporal Gyrus		–	8.23
	300	Inferior Frontal Gyrus, Middle Frontal Gyrus, BA8, BA9	(44,4,28)	Inferior Frontal Gyrus	R	–	8.18
			(54,8,24)	Inferior Frontal Gyrus		–	7.71
			(54,18,36)	Middle Frontal Gyrus		–	5.99
	153	Superior Parietal Lobule, Inferior Parietal Lobule	(-34,-64,54)	Superior Parietal Lobule	L	7	7.72
			(-36,-54,52)	Inferior Parietal Lobule		–	5.22
	203	Middle Frontal Gyrus , Inferior Frontal Gyrus	(-52,6,28)	Inferior Frontal Gyrus	L	–	7.36
			(-50,6,44)	Middle Frontal Gyrus		–	5.30
	164	Middle Occipital Gyrus, Superior Occipital Gyrus BA19	(-24,-88,26)	Cuneus	L	–	6.06
			(-26,-76,20)			–	5.93
			(-20,-90,16)			–	4.82
	164	–	(-24,-88,26)	Cuneus	R	7	6.06
	34	–	(40,-2,12)	Insula	R	–	6.41
	67	–	(52,30,22)	Middle Frontal Gyrus	R	46	6.13
	56	–	(36,-12,16)	Insula	R	13	6.05

Table S3: Repeatability of behavioral experimental results in the CC and QA tasks.

**Subject**	**QA Task Repeatability Rate**			**CC Task Repeatability Rate**	**All Repeatability Rate**
	**Repeatability**	**SRCC**	**PLCC**		
Subject 01	0.91	0.93	0.93	0.98	0.94
Subject 02	0.92	0.94	0.94	0.92	0.92
Subject 03	0.91	0.93	0.93	0.98	0.94
Subject 04	0.68	0.76	0.76	0.97	0.83
Subject 11	0.78	0.81	0.80	0.94	0.86
Subject 12	0.86	0.89	0.89	0.96	0.91
Subject 13	0.82	0.79	0.77	0.93	0.88
Subject 14	0.77	0.82	0.80	0.91	0.85

Table S4: The power analysis of the statistical results regarding the semantic content in the calcarine, cuneus, and superior occipital gyrus (SOG).

**ROI**	**Levene Test**	**Welch-ANOVA**		**ANOVA Test Power**	**Mento Carlo Simulation Power**
	p-value	p-value	η^2		
Calcarine	0.028	0.002	0.386	0.996	0.961
Cuneus	0.002	0.008	0.331	0.982	0.872
SOG	0.192	0.011	0.269	0.934	0.828

Table S5: The power analysis of the statistical results regarding the test results of high, neutral, and low-quality data on SVR models trained using low-quality response patterns. The SVR models between "ROI A - ROI B" pairs are built to predict the response patterns of ROI B using the response patterns ROI A. SOG, MOG, IOG refer to superior occipital gyrus, middle occipital gyrus and inferior occipital gyrus, respectively.

**ROI**	**Levene Test**	**Welch-ANOVA**		**ANOVA**	**Mento Carlo**
	p-value	p-value	η^2	**Test Power**	**Simulation Power**
Calcarine-MOG	0.135	0.014	0.242	0.894	0.812
Calcarine-IOG	0.987	0.019	0.209	0.825	0.775
Cuneus-Lingual	0.639	0.027	0.170	0.716	0.729
Cuneus-MOG	0.925	0.008	0.251	0.908	0.867
Cuneus-IOG	0.856	0.014	0.235	0.880	0.819
Lingual-MOG	0.016	0.005	0.280	0.946	0.895
Lingual-IOG	0.778	0.027	0.196	0.792	0.724
SOG-MOG	0.293	0.014	0.216	0.843	0.803
SOG-IOG	0.682	0.030	0.203	0.811	0.709
MOG-IOG	0.727	0.012	0.246	0.901	0.823

3 Supplemental methods

3.1 Supplemental fMRI experimental setup

First, all participants complete a Snellen visual acuity test prior to the experiment and confirm that they have normal or corrected-to-normal vision, defined as a Snellen acuity score of 20/20 at a distance of 6 meters.

Then, all participants were provided with instructions and a demo run. The structure of the demo run's stimulus presentation mirrored that of the formal experiment. However, during the image presentations, reference labels corresponding to the current task were displayed at the bottom of each image. Specifically, during the Content Classification task, semantic reference labels were shown, and during the Quality Assessment task, quality reference labels were displayed. The purpose of the demo run was to establish a unified quality assessment standard for all participants and to familiarize them with the experimental procedure, thereby reducing potential anxiety and unexpected issues during the formal experiment. Importantly, all stimuli presented in the demo run were not used in the formal experiment.

Visual stimulation is presented using the SINORAD SA-9939 Brain Functional Audiovisual Stimulation System. This system is equipped with two button feedback devices, one for each hand, with two buttons on each device. During the experiment, participants are instructed to press the buttons with their thumbs. In the Quality Assessment task, the left button on the left hand represents low quality, the right button on the left hand represents neutral quality, and the left button on the right hand represents excellent quality. In the Content Classification task, the left button on the left hand represents 'face', the right button on the left hand represents 'object', and the left button on the right hand represents 'scene'

The instructions for participant include:

"The upcoming experiment will be divided into eight consecutive scans. During the scans, please remain relaxed and try to avoid any movement, especially of the head. Prior to each scan, we will provide a verbal cue. At the beginning and end of each scan, a cross will appear at the center of the screen. When you see the cross, please try to keep your body and head stable, remain relaxed, and avoid any thoughts or spontaneous movements. After the first cross disappears, the words 'Quality Assessment' or 'Content Classification' will appear. Please perform the corresponding task when the images are presented. During the task, please try to focus your attention and ensure a timely response to each image presented. Avoid distractions during the task, refrain from making any unnecessary movements, and keep your eyes fixed on the images displayed on the screen. When the 'Quality Assessment' label appears, provide your subjective visual quality rating for each image and press the button in your hand: the left button on your left hand represents low quality, the right button on your left hand represents neutral quality, and the left button on your right hand represents excellent quality. When the 'Content Classification' label appears, please categorize each image semantically by pressing the corresponding button: the left button on your left hand represents 'face', the right button on your left hand represents 'object', and the left button on your right hand represents 'scene'. Please press the button during image presentation with your thumbs. If you miss an image, do not worry; simply complete the task for the current image when the next image is presented. The task will remain unchanged until new instructions appear. If you feel any discomfort during the experiment, you can speak or use hand or leg gestures, and we will stop the experiment immediately. Thank you for your cooperation."

3.2 Supplemental fMRI data preprocessing

All MRI data were converted into Brain Imaging Data Structure (BIDS) using dcm2niix (version v1.0.20220720). Results included in this manuscript come from preprocessing performed using *fMRIPrep* 23.1.0[1,2] which is based on *Nipype* 1.8.6[3,4].

3.2.1 Preprocessing of B0 inhomogeneity mappings

A total of 1 fieldmaps were found available within the input BIDS structure for this particular participant. A *B0* nonuniformity map (or *fieldmap*) was estimated from the phase-drift map(s) measure with two consecutive GRE (gradient-recalled echo) acquisitions. The corresponding phase-map(s) were phase-unwrapped with `prelude` (FSL None).

3.2.2 Anatomical data preprocessing

A total of 1 T1-weighted (T1w) images were found within the input BIDS dataset.The T1-weighted (T1w) image was corrected for intensity non-uniformity (INU) with `N4BiasFieldCorrection`[5], distributed with ANTs (version unknown)[6] RRID:SCR_004757, and used as T1w-reference throughout the workflow. The T1w-reference was then skull-stripped with a *Nipype* implementation of the `antsBrainExtraction.sh` workflow (from ANTs), using OASIS30ANTs as target template. Brain tissue segmentation of cerebrospinal fluid (CSF), white-matter (WM) and gray-matter (GM) was performed on the brain-extracted T1w using `fast` FSL (version unknown), RRID:SCR_002823,[7]. Brain surfaces were reconstructed using `recon-all` FreeSurfer 7.3.2, RRID:SCR_001847,[8], and the brain mask estimated previously was refined with a custom variation of the method to reconcile ANTs-derived and FreeSurfer-derived segmentations of the cortical gray-matter of Mindboggle RRID:SCR_002438,[9]. Volume-based spatial normalization to one standard space (MNI152NLin2009cAsym) was performed through nonlinear registration with `antsRegistration` (ANTs (version unknown)), using brain-extracted versions of both T1w reference and the T1w template. The following templates was were selected for spatial normalization and accessed with *TemplateFlow* 23.0.0,[10]: *ICBM 152 Nonlinear Asymmetrical template version 2009c* [Fonov et al.[11], RRID:SCR_008796; TemplateFlow ID: MNI152NLin2009cAsym].

3.2.3 Functional data preprocessing

For each of the 8 BOLD runs found per participant (across all tasks and sessions), the following preprocessing was performed. First, a reference volume and its skull-stripped version were generated using a custom methodology of *fMRIPrep*. Head-motion parameters with respect to the BOLD reference (transformation matrices, and six corresponding rotation and translation parameters) are estimated before any spatiotemporal filtering using `mcflirt` FSL ,[12]. The estimated *fieldmap* was then aligned with rigid-registration to the target EPI (echo-planar imaging) reference run. The field coefficients were mapped on to the reference EPI using the transform. BOLD runs were slice-time corrected to 0.961s (0.5 of slice acquisition range 0s-1.92s) using `3dTshift` from AFNI[13] RRID:SCR_005927. The BOLD reference was then co-registered to the T1w reference using `bbregister` (FreeSurfer) which implements boundary-based registration[14]. Co-registration was configured with six degrees of freedom. Several confounding time-series were calculated based on the *preprocessed BOLD*: framewise displacement (FD), DVARS and three region-wise global signals. FD was computed using two formulations following Power (absolute sum of relative motions, Power et al.[15]) and Jenkinson (relative root mean square displacement between affines, Jenkinson et al.[12]). FD and DVARS are calculated for each functional run, both using their implementations in *Nipype* following the definitions by[15]. The three global signals are extracted within the CSF, the WM, and the whole-brain masks. Additionally, a set of physiological regressors were extracted to allow for component-based noise correction *CompCor*,[16]. Principal components are estimated after high-pass filtering the *preprocessed BOLD* time-series (using a discrete cosine filter with 128s cut-off) for the two *CompCor* variants: temporal (tCompCor) and anatomical (aCompCor). tCompCor components are then calculated from the top 2% variable voxels within the brain mask. For aCompCor, three probabilistic masks (CSF, WM and combined CSF+WM) are generated in anatomical space. The implementation differs from that of Behzadi et al. in that instead of eroding the masks by 2 pixels on BOLD space, a mask of pixels that likely contain a volume fraction of GM is subtracted from the aCompCor masks. This

mask is obtained by dilating a GM mask extracted from the FreeSurfer's *aseg* segmentation, and it ensures components are not extracted from voxels containing a minimal fraction of GM. Finally, these masks are resampled into BOLD space and binarized by thresholding at 0.99 (as in the original implementation). Components are also calculated separately within the WM and CSF masks. For each CompCor decomposition, the *k* components with the largest singular values are retained, such that the retained components' time series are sufficient to explain 50 percent of variance across the nuisance mask (CSF, WM, combined, or temporal). The remaining components are dropped from consideration. The head-motion estimates calculated in the correction step were also placed within the corresponding confounds file. The confound time series derived from head motion estimates and global signals were expanded with the inclusion of temporal derivatives and quadratic terms for each[17]. Frames that exceeded a threshold of 0.5 mm FD or 1.5 standardized DVARS were annotated as motion outliers. Additional nuisance time-series are calculated by means of principal components analysis of the signal found within a thin band (*crown*) of voxels around the edge of the brain, as proposed by[18]. The BOLD time-series were resampled into standard space, generating a *preprocessed BOLD run in MNI152NLin2009cAsym space*. First, a reference volume and its skull-stripped version were generated using a custom methodology of *fMRIPrep*. All resamplings can be performed with *a single interpolation step* by composing all the pertinent transformations (i.e. head-motion transform matrices, susceptibility distortion correction when available, and co-registrations to anatomical and output spaces). Gridded (volumetric) resamplings were performed using `antsApplyTransforms` (ANTs), configured with Lanczos interpolation to minimize the smoothing effects of other kernels[19]. Non-gridded (surface) resamplings were performed using `mri_vol2surf` (FreeSurfer).

4 Supplemental notes

4.1 Supplemental of univariate analysis

For the contrast between two task conditions, QA-CC, in the first-level analysis, we modeled the Quality Assessment, Content Classification, and resting-state corresponding beta maps for eight runs using SPM, with the block start time points and durations. The contrast metric for each run is structured as (QA, CC, resting-state). Given that each run includes QA, CC, and resting-state conditions with equal weighting across runs, the full contrast metric is derived by repeating the vector (0.125, -0.125, 0) eight times, corresponding to the eight runs. The contrast matrix for QA-CC is:

$$(0.125, -0.125, 0, 0.125, -0.125, 0, 0.125, -0.125, 0, 0.125, -0.125, 0, \\ 0.125, -0.125, 0, 0.125, -0.125, 0, 0.125, -0.125, 0, 0.125, -0.125, 0)$$

For the contrast between the three quality conditions in the first-level analysis, we modeled the beta maps corresponding to bad quality, neutral quality, and excellent quality for eight runs using SPM, with the event start time points and durations. The contrast metric for each run is structured as (bad quality, neutral quality, excellent quality). Each run includes bad quality, neutral quality, excellent quality conditions with equal weighting across runs, the full contrast metric is derived by repeating the corresponding vector eight times, corresponding to the eight runs. The contrast matrix for the contrast of excellent quality - bad quality is:

$$(-0.125, 0, 0.125, -0.125, 0, 0.125, -0.125, 0, 0.125, -0.125, 0, 0.125, \\ -0.125, 0, 0.125, -0.125, 0, 0.125, -0.125, 0, 0.125, -0.125, 0, 0.125)$$

The contrast matrix for the contrast of excellent quality - neutral quality is:

$$(0, -0.125, 0.125, 0, -0.125, 0.125, 0, -0.125, 0.125, 0, -0.125, 0.125, \\ 0, -0.125, 0.125, 0, -0.125, 0.125, 0, -0.125, 0.125, 0, -0.125, 0.125)$$

The contrast matrix for the contrast of neutral quality - bad quality is:

$$(-0.125, 0.125, 0, -0.125, 0.125, 0, -0.125, 0.125, 0, -0.125, 0.125, 0, \\ -0.125, 0.125, 0, -0.125, 0.125, 0, -0.125, 0.125, 0, -0.125, 0.125, 0)$$

Since we performed denoising on the fMRI signals prior to the univariate analysis, potential confounding effects characterized by white matter time-series (5 CompCor noise components), CSF time-series (5 CompCor noise components), motion parameters and their first-order derivatives (12 factors), outlier scans (up to 89 factors), and linear trends (2 factors) within each functional run were already accounted for. Therefore, in the first-level analysis, we did not include any other covariates beyond the conditions of interest for contrast.

4.2 Supplemental Button-press data

4.2.1 Analysis of button-press data

During the experiment, participants are instructed to press designated buttons to indicate their task responses. We monitor their feedback in real-time from the control room, and if response accuracy falls below an acceptable level, the scanning run is immediately halted and repeated. This protocol is necessary given the high cost of fMRI acquisition, as evaluating response accuracy only after the session could lead to substantial data loss.

Unfortunately, due to instability in the response logging system, a portion of the button-press data was not successfully recorded. However, the corresponding fMRI data meet all technical quality standards, and

no anomalies were observed during real-time monitoring. As a result, we retain complete behavioral data for eight participants, as shown in Table. S3. The results indicate that participants' judgments varied upon repeated presentations of the same image. Moreover, the repeatability in the Content Classification (CC) task was significantly higher than in the Quality Assessment (QA) task, with repeat rates exceeding 90% for both. Since the QA and CC tasks were interleaved within each run, the lower repeatability observed in the QA task cannot be attributed to participants' lack of effort or engagement. This phenomenon underscores the necessity of the present study: subjective scores collected via rating scales are strongly influenced by individual variability, such that even the same participant exhibits fluctuations in quality judgments for identical images.

It is important to emphasize that we recognize a distinction between physiological signals and participants' explicit behavioral responses. Subjective judgments of visual quality can be influenced by individual experience and may become biased as the experiment progresses—an acknowledged limitation in the field of quality assessment. Therefore, one of our aims is to investigate the neural mechanisms underlying visual quality perception using fMRI. We further posit that physiological signals may offer a more objective and direct reflection of the impact of quality degradation, a view also supported by previous EEG-based studies on visual quality. Accordingly, participants' button responses primarily serve to ensure engagement and sustained attention throughout the experiment.

In accordance with ITU-R BT.500-14 guidelines, our subjective quality assessment experiment employed a mixed cohort of both experienced subjects and inexperienced subjects. The experimental group comprised four experienced subjects (Subjects 01, 02, 03 and 13) alongside inexperienced subjects without professional background. As evidenced in Table S3, the experienced subjects demonstrated higher key-press response consistency during quality rating tasks.

4.2.2 The relationship with fMRI analysis

To mitigate potential perceptual biases, all participants completed a standardized demo session prior to fMRI scanning, incorporating anchor stimuli for individual bias calibration, as shown in the "Detailed Description of the fMRI Experimental Setup" section of SI.

We further examined whether the decoding performance of visual quality information from fMRI data differed between experienced and inexperienced participants. Given that experienced participants exhibited higher response consistency during the quality assessment (QA) task, this comparison also serves to evaluate the potential influence of behavioral response reliability on fMRI results. Specifically, we compared quality decoding performance between experienced participants with available response data (Subjects 01, 02, 03, and 13) and inexperienced participants (Subjects 04, 11, 12, and 14) using a two-tailed t-test (results shown in Figure. S13).

The analysis revealed no statistically significant differences in decoding performance between the two groups across all tested brain region combinations (two-tailed t-test, alpha = 0.05). This indicates that although experienced participants demonstrated superior behavioral consistency in the QA task, their fMRI-derived quality representations did not significantly differ from those of inexperienced participants—justifying our decision to pool data across both groups in the main analyses.

Moreover, these findings provide further evidence that neurophysiological measures (e.g., EEG, fMRI) for visual quality assessment may offer a more objective and direct reflection of quality degradation effects compared to traditional behavioral paradigms. This perspective aligns with prior EEG-based studies on visual quality perception[20,21].

4.3 Supplemental Statistical power analysis

4.3.1 Power analysis for RSA results

For the semantic information in the calcarine, cuneus, and superior occipital gyrus (SOG) across the three quality conditions (bad, neutral, excellent), we set a significance threshold of alpha = 0.05, with 14 participants and 3 conditions, and performed a power analysis. However, traditional power analyses are often based on a standard one-way ANOVA design (assuming homogeneity of variance). Welch ANOVA involves adjusted degrees of freedom (based on sample size and within-group variance), making the power analysis more complex. Therefore, we first conducted a Monte Carlo simulation-based power analysis by generating simulated data based on the original data distribution, simulating the Welch ANOVA test, and calculating the probability of obtaining a p-value greater than 0.05. The simulated probability was used as the reproducibility probability for significant results, yielding the statistical power. The number of simulations for each brain region was 5000.

In additional, for samples with homogeneity of variance, the power analysis of the traditional ANOVA can serve as an approximation to the Welch ANOVA power analysis. Therefore, we first conducted Levene's test for homogeneity of variance, providing the Levene test statistic and p-value, as well as the partial eta-squared effect size np2 and power values from the traditional ANOVA power analysis, as supplementary references. This analysis was performed using the *FTestAnovaPower* function in the *statsmodels* Python library.

The results are shown in the Table S4. The power analysis corresponding to traditional ANOVA and Monte Carlo simulation shows that our three brain regions achieve a statistical power greater than 0.8 in both methods. According to[22], a partial eta-squared greater than 0.14 indicates a significant between-group difference. The results of Welch ANOVA reveal that the partial eta-squared values for all three brain regions exceed 0.14. Thanks to the relatively large effect sizes, the sample size in this study (n = 14) provides robust support for our findings."

4.3.2 Power analysis for the results in "Building the response pattern prediction model between ROIs" section in the "Results"

We perform a power analysis on the statistical results in "Building the response pattern prediction model between ROIs" section in the "Results". In this section, we note that, "Low-quality training datasets are used to train the SVR model, mapping representation vectors between ROIs A and B. A model adapte to low-quality images would perform best on low-quality tests. As shown in Fig. 4a, the predictive performance of the model predicting the middle occipital gyrus and the inferior occipital gyrus from the other ROIs, as well as the model predicting the lingual from the cuneus, is significantly correlated with quality conditions, with significantly better performance on low-quality tests than on neutral or high-quality ones."

We applied the same methodology as described above, performing statistical power analysis on the prediction performance of the brain region pairs that exhibited significant effects in Fig. 4a, using both traditional ANOVA-based power analysis and Monte Carlo simulation. A significance threshold of alpha = 0.05 was set, with 14 participants and 3 conditions (bad quality, neutral quality, and excellent quality). Similarly, we present the results of the Levene test for homogeneity of variance, the p-values from Welch ANOVA, partial eta-squared effect sizes np2, and the power values obtained from both traditional ANOVA power analysis and Monte Carlo simulation-based power analysis. The number of simulations for each brain region pair was set to 5000. The results are shown in the Table S5 below. The power analysis corresponding to traditional ANOVA and Monte Carlo simulation shows that six brain region pairs achieve a statistical power greater than 0.8 in both methods and the rest of four also achieve a statistical power greater than 0.7 in both methods. The results of Welch ANOVA reveal that the partial eta-squared values for all brain region pairs exceed 0.14. Thanks to the relatively large effect sizes, the sample size in this study (n = 14) provides robust support for our findings.

Supplemental References

1. Esteban, O., Markiewicz, C., Blair, R.W., Moodie, C., Isik, A.I., Erramuzpe Aliaga, A., Kent, J., Goncalves, M., DuPre, E., Snyder, M. et al. (2019). fMRIPrep: a robust preprocessing pipeline for functional MRI. Nat. Methods *16*, 111–116.

2. Esteban, O., Blair, R., Markiewicz, C.J., Berleant, S.L., Moodie, C., Ma, F., Isik, A.I., Erramuzpe, A., Kent, M., James D. andGoncalves, DuPre, E., Sitek, K.R., Gomez, D.E.P., Lurie, D.J., Ye, Z., Poldrack, R.A., and Gorgolewski, K.J. (2018). fmriprep. `https://doi.org/10.5281/zenodo.852659`.

3. Gorgolewski, K., Burns, C.D., Madison, C., Clark, D., Halchenko, Y.O., Waskom, M.L., and Ghosh, S. (2011). Nipype: a flexible, lightweight and extensible neuroimaging data processing framework in python. Front. Neuroinform. *5*, 13.

4. Gorgolewski, K.J., and Esteban (2018). Nipype. `https://doi.org/10.5281/zenodo.596855`.

5. Tustison, N.J., Avants, B.B., Cook, P.A., Zheng, Y., Egan, A., Yushkevich, P.A., and Gee, J.C. (2010). N4itk: Improved n3 bias correction. IEEE Trans. Med. Imaging *29*, 1310–1320.

6. Avants, B., Epstein, C., Grossman, M., and Gee, J. (2008). Symmetric diffeomorphic image registration with cross-correlation: Evaluating automated labeling of elderly and neurodegenerative brain. Med. Image Anal. *12*, 26–41.

7. Zhang, Y., Brady, M., and Smith, S. (2001). Segmentation of brain MR images through a hidden markov random field model and the expectation-maximization algorithm. IEEE Trans. Med. Imaging *20*, 45–57.

8. Dale, A.M., Fischl, B., and Sereno, M.I. (1999). Cortical surface-based analysis: I. segmentation and surface reconstruction. NeuroImage *9*, 179–194.

9. Klein, A., Ghosh, S.S., Bao, F.S., Giard, J., Häme, Y., Stavsky, E., Lee, N., Rossa, B., Reuter, M., Neto, E.C. et al. (2017). Mindboggling morphometry of human brains. PLOS Comput. Biol. *13*, e1005350.

10. Ciric, R., Thompson, W.H., Lorenz, R., Goncalves, M., MacNicol, E., Markiewicz, C.J., Halchenko, Y.O., Ghosh, S.S., Gorgolewski, K.J., Poldrack, R.A., and Esteban, O. (2022). TemplateFlow: FAIR-sharing of multi-scale, multi-species brain models. Nat. Methods *19*, 1568–1571.

11. Fonov, V., Evans, A., McKinstry, R., Almli, C., and Collins, D. (2009). Unbiased nonlinear average age-appropriate brain templates from birth to adulthood. NeuroImage *47, Supplement 1*, S102.

12. Jenkinson, M., Bannister, P., Brady, M., and Smith, S. (2002). Improved optimization for the robust and accurate linear registration and motion correction of brain images. NeuroImage *17*, 825–841.

13. Cox, R.W., and Hyde, J.S. (1997). Software tools for analysis and visualization of fmri data. NMR Biomed. *10*, 171–178.

14. Greve, D.N., and Fischl, B. (2009). Accurate and robust brain image alignment using boundary-based registration. NeuroImage *48*, 63–72.

15. Power, J.D., Mitra, A., Laumann, T.O., Snyder, A.Z., Schlaggar, B.L., and Petersen, S.E. (2014). Methods to detect, characterize, and remove motion artifact in resting state fmri. NeuroImage *84*, 320–341.

16. Behzadi, Y., Restom, K., Liau, J., and Liu, T.T. (2007). A component based noise correction method (CompCor) for BOLD and perfusion based fmri. NeuroImage *37*, 90–101.

17. Satterthwaite, T.D., Elliott, M.A., Gerraty, R.T., Ruparel, K., Loughead, J., Calkins, M.E., Eickhoff, S.B., Hakonarson, H., Gur, R.C., Gur, R.E., and Wolf, D.H. (2013). An improved framework for confound regression and filtering for control of motion artifact in the preprocessing of resting-state functional connectivity data. NeuroImage *64*, 240–256.

18. Patriat, R., Reynolds, R.C., and Birn, R.M. (2017). An improved model of motion-related signal changes in fMRI. NeuroImage *144, Part A*, 74–82.

19. Lanczos, C. (1964). Evaluation of noisy data. J. Soc. Ind. Appl. Math. Ser. B Numer. Anal. *1*, 76–85.

20. Hu, S., Duan, Y., Tao, X., Li, G.Y., Lu, J., Liu, G., Zheng, Z., and Pan, C. (2024). Brain-inspired image perceptual quality assessment based on eeg: A qoe perspective. IEEE Trans. Pattern Anal. Mach. Intell. *46*, 8424–8441.

21. Arndt, S., Antons, J.N., Schleicher, R., Möller, S., and Curio, G. (2014). Using electroencephalography to measure perceived video quality. IEEE J. Sel. Top. Signal Process. *8*, 366–376.

22. Cohen, J. (1988). Statistical Power Analysis for the Behavioral Sciences. 2nd ed.. Lawrence Erlbaum Associates.
